# New Thieno[3,2-*d*]pyrimidin-4(3*H*)-one Schiff Bases as Selective Antileishmanial Agents

**DOI:** 10.3390/life16060979

**Published:** 2026-06-10

**Authors:** Neriman Mor, Barış Yıldız, Baycan Mor, Feyzi Sinan Tokalı

**Affiliations:** 1 Department of Medical Parasitology, Faculty of Medicine, Kafkas University, 36100 Kars, Türkiye; 2 Life Sciences and Technologies Application and Research Center, Kafkas University, 36000 Kars, Türkiye; baris.yldz@outlook.com; 3 Department of Molecular Biology and Genetics, Faculty of Sciences and Letters, Kafkas University, 36100 Kars, Türkiye; baycanmor@hotmail.com; 4 Department of Material and Material Processing Technologies, Kars Vocational School, Kafkas University, 36100 Kars, Türkiye

**Keywords:** leishmaniasis, thieno[3,2-*d*]pyrimidin-4(3*H*)-one, *L. major*, Schiff bases, antileishmania

## Abstract

The present study aimed to design, synthesize, and evaluate a new series of thieno[3,2-*d*]pyrimidin-4(3*H*)-one-based Schiff bases as potential antileishmanial agents against *Leishmania major* (*L. major*). A series of twenty thieno[3,2-*d*]pyrimidine Schiff base derivatives were synthesized and characterized using FTIR, NMR, and HRMS techniques. Their antipromastigote activities were evaluated in vitro against *L. major*, while cytotoxic effects were assessed on HUVECs to determine selectivity indices. The most active compound was further investigated using molecular docking against several *L. major* proteins. Among the tested compounds, compound **12**, bearing a 2-hydroxy-5-bromophenyl moiety, exhibited the most potent activity against *L. major* promastigotes with an IC_50_ value of 13.7 µM, along with a favorable selectivity index (SI = 17.5), outperforming the reference drug miltefosine (IC_50_ = 31 µM and SI = 0.2). Docking studies demonstrated that compound **12** showed the strongest binding affinity toward phosphodiesterase B1, supported by a docking score of −9.042 kcal/mol and an MM-GBSA value of −67.21 kcal/mol. This study highlights thieno[3,2-*d*]pyrimidin-4(3*H*)-one as a promising scaffold in the context of in vitro antileishmanial screening and suggests the role of ortho-phenolic substitution in enhancing activity and selectivity. Compound **12** emerges as a promising lead, warranting further optimization and biological evaluation in future studies.

## 1. Introduction

Leishmaniasis is one of the neglected tropical diseases caused by obligate intracellular protozoa of the genus *Leishmania*, transmitted via sandflies as vectors. Clinically manifesting in cutaneous, mucocutaneous, and visceral forms, the disease constitutes a significant public health problem in endemic regions. Millions of people worldwide are reported to be infected, and hundreds of millions more live at risk of infection [1,2]. The prevalence of the disease is further exacerbated by factors such as socioeconomic conditions, migration, climate change, and immunosuppression. It poses a significant economic and social burden on healthcare systems, not only in terms of mortality and morbidity, but also due to the need for long-term treatment, the risk of relapse, and serious side effects [3].

*L. major*, one of the causative agents of cutaneous leishmaniasis, has a wide geographical distribution, primarily in the Middle East, the Mediterranean basin, Africa, and Central Asia. *L. major* infections typically present with ulcerative skin lesions, leading to permanent scarring and significant aesthetic problems [4,5]. Although the disease is not life-threatening, research into treatment is of great importance, especially in endemic regions, as it significantly reduces the quality of life of individuals and causes social stigmatization [6,7]. Current treatment options for leishmaniasis, such as pentavalent antimony compounds, amphotericin B, and miltefosine, have significant limitations including toxicity, the need for parenteral administration, long treatment durations, and increasing drug resistance [8]. Miltefosine, while an important alternative due to its oral applicability, highlights the need for newer and safer antileishmanial agents due to its gastrointestinal side effects, teratogenicity risk, and resistance development [9]. In this context, the development of compounds with novel chemical frameworks, high antileishmanial efficacy, and low toxicity to host cells is a key focus of current research.

**Figure 1:**
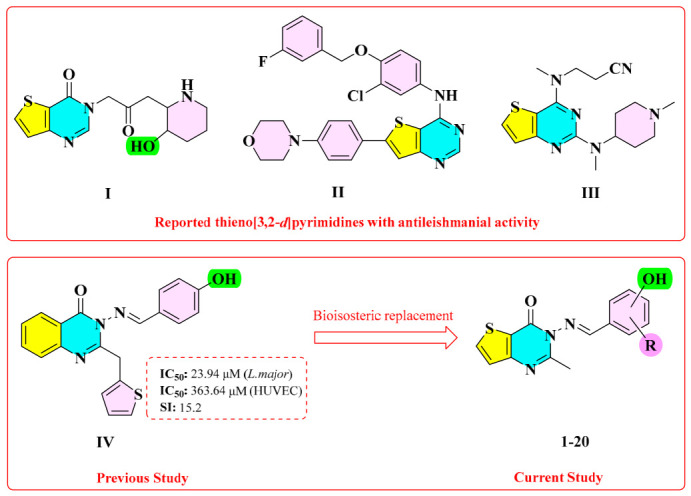
The design strategy for compounds **1–20**.

Nitrogen-containing fused heterocyclic rings are considered privileged structures in drug design and discovery, exhibiting a broad spectrum of biological activity. Among these structural classes, thieno[3,2-*d*]pyrimidine derivatives offer a promising molecular platform in the design of biologically active molecules [10,11,12,13]. However, although some antiparasitic derivatives of this skeleton have been reported (I–III), their potential against *Leishmania* species has been addressed in only a limited number of studies to date (Figure 1) [14,15,16]. In contrast, quinazoline derivatives, considered bioisosteres of thieno[3,2-*d*]pyrimidines, are a class of compounds frequently investigated in the literature due to their antileishmanial activity [17]. Indeed, a recent study conducted by our research group reported that various quinazoline derivatives exhibited significant antileishmanial activity against *L. major* [18]. In line with these findings, the aim was to develop novel compounds with a thieno[3,2-*d*]pyrimidine core, based on structural and biological inferences obtained from the quinazoline skeleton, within a rational design strategy.

In a previous study by our research group, a series of Schiff bases with quinazolin-4(3*H*)-one backbone were synthesized and their antileishmanial activity against *L. major* was evaluated. In that study, the quinazolin-4(3*H*)-one core was kept constant and derivatization was carried out using various substituted aromatic aldehydes to reveal the structure–activity relationship. The results showed that the derivative containing a phenolic OH group (IV) exhibited higher antileishmanial activity compared to other compounds in the series (Figure 1) [18]. This finding indicated that phenolic functional groups can positively influence biological activity through hydrogen bond interactions with parasitic targets. Based on these data, in order to increase both biological activity and selectivity, the thieno[3,2-*d*]pyrimidine-4(3*H*)-one backbone, which is considered its bioisostere, was preferred instead of the quinazolin-4(3*H*)-one core used in the previous study. Furthermore, considering the significant contribution of the phenolic structure to the activity in the previous study, in this study the thieno[3,2-*d*]pyrimidine-4(3*H*)-one core was derived with phenolic aldehydes to form Schiff bases, thus designing a new series of compounds.

This study focuses on the thieno[3,2-*d*]pyrimidine-4(3*H*)-one scaffold, whose antipromastigote potential against *L. major* has been investigated to a limited extent in the literature. The antipromastigote activities of the synthesized compounds were evaluated against *L. major*, their toxicity to host cells was investigated using the HUVEC line, and the results were compared with the reference drug miltefosine. In addition, molecular docking studies were performed against selected proteins isolated from *L. major* in order to predict the possible mechanism of action of the most active compound.

## 2. Materials and Methods

### 2.1. Chemistry

All chemicals used in this study were obtained from various commercial suppliers. Melting points of the synthesized compounds were measured using a WRS-2A Microprocessor Melting-point Apparatus (Hangzhou, China) and are reported without correction. ^1^H NMR spectra were acquired using a Bruker 400 MHz spectrometer, while ^13^C NMR spectra were obtained on a Bruker 100 MHz instrument (Bruker Switzerland AG, Faelanden, Switzerland). Chemical shifts are reported in δ (ppm) relative to tetramethylsilane (TMS, δ 0.00 singlet) using deuterated dimethyl sulfoxide (DMSO-*d*_6_) as the solvent. FTIR spectra of compounds were recorded using an Alpha-P Bruker FTIR spectrophotometer (Bruker Switzerland AG, Faelanden, Switzerland). HRMS spectra were recorded on an Agilent 6530 True Mass Q-TOF LC/MS system (Santa Clara, CA, USA).

#### 2.1.1. Synthesis of Compound **TPM**

**Figure 2:**
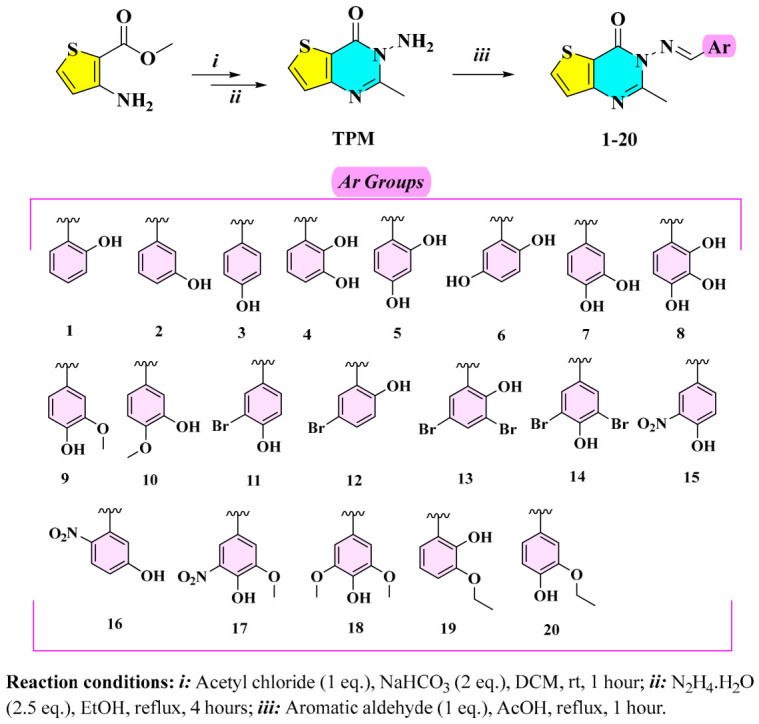
The synthetic route for the preparation of compounds **1–20**.

Methyl 3-aminothiophene-2-carboxylate (10 mmol) was first dissolved in 20 mL of dichloromethane, and sodium bicarbonate (20 mmol) was added to the solution. The mixture was cooled in an ice bath, and a solution of acetyl chloride (10 mmol) in 10 mL of dichloromethane was introduced dropwise. The reaction was stirred at room temperature for one hour to ensure complete acylation. Afterward, the mixture was filtered to remove solids, and the solvent was evaporated under reduced pressure. The resulting crude intermediate was dissolved in 20 mL of absolute ethanol, followed by the addition of hydrazinium hydroxide (25 mmol), and the reaction mixture was refluxed for four hours. Upon cooling to room temperature, the precipitated product was collected by filtration, and the final compound was purified through recrystallization from ethanol (Figure 2) [13].

3-Amino-2-methylthieno[3,2-*d*]pyrimidin-4(3*H*)-one **(TPM)** [13]

White crystals, yield: %90. FTIR (cm^−1^): ν_max_ 3250, 3131, 3071, 1646, 1586, 1203. ^1^H NMR (400 MHz, CDCl_3_) δ 7.75 (d, *J* = 5.2 Hz, 1H, ArH), 7.23 (d, *J* = 5.2 Hz, 1H, ArH), 4.99 (s, 2H, NH_2_), 2.71 (s, 3H, CH_3_). ^13^C NMR (100 MHz, CDCl_3_) δ 157.8, 156.8, 155.9, 134.5, 124.9, 120.2, 22.0. HRMS-Q-TOF (*m*/*z*) calcd for C_7_H_7_N_3_OS: 182.0388 [M + H]^+^, found: 182.0372.

#### 2.1.2. Synthesis of the Target Compounds (**1–20**)

To a solution of TPM (10 mmol) in glacial acetic acid (6 mL), the aromatic aldehyde (10 mmol) was added and the solution was refluxed for one hour. The solvent was removed under reduced pressure and the crude product was recrystallized from ethanol (Figure 2) [19].

(*E*)-3-((2-Hydroxybenzylidene)amino)-2-methylthieno[3,2-*d*]pyrimidin-4(3*H*)-one **(1)**

White solid, yield: %92. FTIR (cm^−1^): ν_max_ 3165, 3078, 1667, 1603, 1267. ^1^H NMR (400 MHz, DMSO) δ 10.57 (s, 1H, OH), 9.05 (s, 1H, N = CH), 8.16 (d, *J* = 5.3 Hz, 1H, ArH), 7.89 (d, *J* = 7.7 Hz, 1H, ArH), 7.45 (t, *J* = 6.9 Hz, 1H, ArH), 7.34 (d, *J* = 5.3 Hz, 1H, ArH), 7.00–6.94 (m, 2H, ArH), 2.48 (s, 3H, CH_3_). ^13^C NMR (101 MHz, DMSO) δ 167.9, 159.3, 155.8, 155.3, 154.7, 136.4, 135.2, 128.9, 125.5, 121.4, 120.3, 118.7, 117.4, 22.8. HRMS-Q-TOF (*m*/*z*) calcd for C_14_H_11_N_3_O_2_S: 286.0650 [M + H]^+^, found: 286.0645.

(*E*)-3-((3-Hydroxybenzylidene)amino)-2-methylthieno[3,2-*d*]pyrimidin-4(3*H*)-one **(2)**

White solid, yield: %90. FTIR (cm^−1^): ν_max_ 3094, 1682, 1609, 1276. ^1^H NMR (400 MHz, DMSO) δ 9.91 (s, 1H, OH), 8.81 (s, 1H, N=CH), 8.14 (d, *J* = 5.3 Hz, 1H, ArH), 7.41–7.27 (m, 4H, ArH), 7.02 (d, *J* = 7.5 Hz, 1H, ArH), 2.47 (s, 3H, CH_3_). ^13^C NMR (101 MHz, DMSO) δ 170.7, 158.5, 155.8, 155.3, 154.6, 136.4, 134.0, 130.9, 125.5, 121.5, 121.2, 120.83, 114.9, 22.7. HRMS-Q-TOF (*m*/*z*) calcd for C_14_H_11_N_3_O_2_S: 286.0650 [M + H]^+^, found: 286.0655.

(*E*)-3-((4-Hydroxybenzylidene)amino)-2-methylthieno[3,2-*d*]pyrimidin-4(3*H*)-one **(3)**

White solid, yield: %94. FTIR (cm^−1^): ν_max_ 3226, 3097, 1683, 1606, 1274. ^1^H NMR (400 MHz, DMSO) δ 10.38 (s, 1H, OH), 8.71 (s, 1H, N=CH), 8.17 (d, *J* = 5.3 Hz, 1H, ArH), 7.80 (d, *J* = 8.6 Hz, 2H, ArH), 7.36 (d, *J* = 5.3 Hz, 1H, ArH), 6.94 (d, *J* = 8.6 Hz, 2H, ArH), 2.48 (s, 3H, CH_3_). ^13^C NMR (100 MHz, DMSO) δ 169.7, 161.9, 155.2, 154.7, 154.1, 135.5, 131.1, 124.8, 123.1, 120.8, 116.0, 22.0. HRMS-Q-TOF (*m*/*z*) calcd for C_14_H_11_N_3_O_2_S: 286.0650 [M + H]^+^, found: 286.0627.

(*E*)-3-((2,3-Dihydroxybenzylidene)amino)-2-methylthieno[3,2-*d*]pyrimidin-4(3*H*)-one **(4)**

Beige solid, yield: %87. FTIR (cm^−1^): ν_max_ 3145, 3047, 1685, 1599, 1261. ^1^H NMR (400 MHz, DMSO) δ 9.88 (s, 1H, OH), 9.75 (s, 1H, OH), 9.06 (s, 1H, N=CH), 8.14 (d, *J* = 5.2 Hz, 1H, ArH), 7.33–7.30 (m, 2H, ArH), 7.01 (d, *J* = 7.8 Hz, 1H, ArH), 6.79 (t, *J* = 7.8 Hz, 1H, ArH), 2.48 (s, 3H, CH_3_). ^13^C NMR (101 MHz, DMSO) δ 169.0, 155.8, 155.2, 154.7, 148.4, 146.7, 136.3, 125.5, 121.5, 120.2, 120.1, 119.5, 119.0, 22.8. HRMS-Q-TOF (*m*/*z*) calcd for C_14_H_11_N_3_O_3_S: 302.0599 [M + H]^+^, found: 302.0592.

(*E*)-3-((2,4-Dihydroxybenzylidene)amino)-2-methylthieno[3,2-*d*]pyrimidin-4(3*H*)-one **(5)**

White solid, yield: %95. FTIR (cm^−1^): ν_max_ 3071, 1674, 1604, 1274. ^1^H NMR (400 MHz, DMSO) δ 10.55 (s, 1H, OH), 10.37 (s, 1H, OH), 8.81 (s, 1H, N=CH), 8.13 (d, *J* = 5.3 Hz, 1H, ArH), 7.67 (d, *J* = 8.4 Hz, 1H, ArH), 7.31 (d, *J* = 5.3 Hz, 1H, ArH), 6.43–6.40 (m, 2H, ArH), 2.45 (s, 3H, CH_3_). ^13^C NMR (101 MHz, DMSO) δ 168.6, 164.1, 161.5, 155.8, 155.3, 154.9, 136.1, 131.5, 125.5, 121.4, 110.3, 109.3, 103.1, 22.7. HRMS-Q-TOF (*m*/*z*) calcd for C_14_H_11_N_3_O_3_S: 302.0599 [M + H]^+^, found: 302.0593.

(*E*)-3-((2,5-Dihydroxybenzylidene)amino)-2-methylthieno[3,2-*d*]pyrimidin-4(3*H*)-one **(6)**

Yellow solid, yield: %89. FTIR (cm^−1^): ν_max_ 3076, 1671, 1607, 1291. ^1^H NMR (400 MHz, DMSO) δ 9.86 (s, 1H, OH), 9.16 (s, 1H, OH), 8.97 (s, 1H, N=CH), 8.16 (d, *J* = 5.3 Hz, 1H, ArH), 7.34 (d, *J* = 5.3 Hz, 1H, ArH), 7.29 (d, *J* = 2.7 Hz, 1H, ArH), 6.92–6.81 (m, 2H, ArH), 2.47 (s, 3H, CH_3_). ^13^C NMR (101 MHz, DMSO) δ 167.6, 155.8, 155.3, 154.8, 152.5, 150.7, 136.4, 125.5, 123.3, 121.5, 118.7, 118.4, 112.7, 22.8. HRMS-Q-TOF (*m*/*z*) calcd for C_14_H_11_N_3_O_3_S: 302.0599 [M + H]^+^, found: 302.0591.

(*E*)-3-((3,4-Dihydroxybenzylidene)amino)-2-methylthieno[3,2-*d*]pyrimidin-4(3*H*)-one **(7)**

White solid, yield: %93. FTIR (cm^−1^): ν_max_ 3319, 3082, 1665, 1603, 1279. ^1^H NMR (400 MHz, DMSO) δ 9.90 (s, 1H, OH), 9.50 (s, 1H, OH), 8.60 (s, 1H, N=CH), 8.11 (d, *J* = 5.3 Hz, 1H, ArH), 7.42 (s, 1H, ArH), 7.30 (d, *J* = 5.3 Hz, 1H, ArH), 7.17 (d, *J* = 8.1 Hz, 1H, ArH), 6.88 (d, *J* = 8.0 Hz, 1H, ArH), 2.44 (s, 3H, CH_3_). ^13^C NMR (101 MHz, DMSO) δ 170.6, 155.8, 155.3, 154.8, 151.3, 146.6, 136.1, 125.4, 124.1, 124.0, 121.5, 116.4, 114.7, 22.7. HRMS-Q-TOF (*m*/*z*) calcd for C_14_H_11_N_3_O_3_S: 302.0599 [M + H]^+^, found: 302.0596.

(*E*)-3-((2,3,4-Trihydroxybenzylidene)amino)-2-methylthieno[3,2-*d*]pyrimidin-4(3*H*)-one **(8)**

Yellow solid, yield: %85. FTIR (cm^−1^): ν_max_ 3532, 3072, 1669, 1275. ^1^H NMR (400 MHz, DMSO) δ 10.24 (s, 1H, OH), 10.06 (s, 1H, OH), 8.82 (s, 1H, N=CH), 8.74 (s, 1H, OH), 8.13 (d, *J* = 5.3 Hz, 1H, ArH), 7.32 (d, *J* = 5.3 Hz, 1H, ArH), 7.12 (d, *J* = 8.6 Hz, 1H, ArH), 6.50 (d, *J* = 8.5 Hz, 1H, ArH), 2.46 (s, 3H, CH_3_). ^13^C NMR (101 MHz, DMSO) δ 170.8, 155.8, 155.2, 154.9, 152.0, 149.8, 136.2, 133.5, 125.5, 122.5, 121.4, 110.8, 109.1, 22.8. HRMS-Q-TOF (*m*/*z*) calcd for C_14_H_11_N_3_O_4_S: 318.0549 [M + H]^+^, found: 318.0543.

(*E*)-3-((4-Hydroxy-3-methoxybenzylidene)amino)-2-methylthieno[3,2-*d*]pyrimidin-4(3*H*)-one **(9)**

White solid, yield: %87. FTIR (cm^−1^): ν_max_ 3531, 3066, 1670, 1262. ^1^H NMR (400 MHz, DMSO) δ 10.05 (s, 1H, OH), 8.67 (s, 1H, N=CH), 8.15 (d, *J* = 5.3 Hz, 1H, ArH), 7.52 (s, 1H, ArH), 7.34–7.32 (m, 2H, ArH), 6.93 (d, *J* = 8.1 Hz, 1H, ArH), 3.84 (s, 3H, OCH_3_), 2.47 (s, 3H, CH_3_). ^13^C NMR (101 MHz, DMSO) δ 170.5, 155.8, 155.3, 154.8, 152.2, 148.8, 136.2, 125.5, 125.4, 124.1, 121.4, 116.3, 111.2, 56.30 22.7. HRMS-Q-TOF (*m*/*z*) calcd for C_15_H_13_N_3_O_3_S: 316.0756 [M + H]^+^, found: 316.0750.

(*E*)-3-((3-Hydroxy-4-methoxybenzylidene)amino)-2-methylthieno[3,2-*d*]pyrimidin-4(3*H*)-one **(10)**

White solid, yield: %90. FTIR (cm^−1^): ν_max_ 3194, 3077, 1672, 1602, 1270. ^1^H NMR (400 MHz, DMSO) δ 9.54 (s, 1H, OH), 8.68 (s, 1H, N=CH), 8.15 (d, *J* = 5.3 Hz, 1H, ArH), 7.44 (s, 1H, ArH), 7.33 (d, *J* = 5.3 Hz, 1H, ArH), 7.29 (d, *J* = 8.4 Hz, 1H, ArH), 7.06 (d, *J* = 8.4 Hz, 1H, ArH), 3.84 (s, 3H, OCH_3_), 2.46 (s, 3H, CH_3_). ^13^C NMR (101 MHz, DMSO) δ 170.5, 155.8, 155.3, 154.7, 152.8, 147.7, 136.3, 125.5, 123.9, 121.5, 114.0, 112.4, 56.4, 22.7. HRMS-Q-TOF (*m*/*z*) calcd for C_15_H_13_N_3_O_3_S: 316.0756 [M + H]^+^, found: 316.0746.

(*E*)-3-((3-Bromo-4-hydroxybenzylidene)amino)-2-methylthieno[3,2-*d*]pyrimidin-4(3*H*)-one **(11)**

White solid, yield: %88. FTIR (cm^−1^): ν_max_ 3092, 1647, 1592, 1291. ^1^H NMR (400 MHz, DMSO) δ 11.24 (s, 1H, OH), 8.73 (s, 1H, N=CH), 8.15 (d, *J* = 5.2 Hz, 1H, ArH), 8.07 (s, 1H, ArH), 7.78 (d, *J* = 8.5 Hz, 1H, ArH), 7.33 (d, *J* = 5.2 Hz, 1H, ArH), 7.10 (d, *J* = 8.4 Hz, 1H, ArH), 2.47 (s, 3H, CH_3_). ^13^C NMR (101 MHz, DMSO) δ 169.1, 159.0, 155.8, 155.3, 154.7, 136.4, 134.2, 130.7, 125.5, 125.3, 121.4, 117.3, 110.7, 22.7. HRMS-Q-TOF (*m*/*z*) calcd for C_14_H_10_BrN_3_O_2_S: 363.9755 [M + H]^+^, found: 363.9747.

(*E*)-3-((5-Bromo-2-hydroxybenzylidene)amino)-2-methylthieno[3,2-*d*]pyrimidin-4(3*H*)-one **(12)**

White solid, yield: %93. FTIR (cm^−1^): ν_max_ 3266, 3076, 1657, 1598, 1285. ^1^H NMR (400 MHz, DMSO) δ 10.85 (s, 1H, OH), 9.05 (s, 1H, N=CH), 8.15 (d, *J* = 5.2 Hz, 1H, ArH), 8.02 (d, *J* = 2.4 Hz, 1H, ArH), 7.57 (dd, *J* = 8.8, 2.4 Hz, 1H, ArH), 7.33 (d, *J* = 5.2 Hz, 1H, ArH), 6.96 (d, *J* = 8.8 Hz, 1H, ArH), 2.49 (s, 3H, CH_3_). ^13^C NMR (101 MHz, DMSO) δ 165.4, 158.4, 155.8, 155.4, 154.7, 137.3, 136.5, 129.9, 125.5, 121.4, 120.9, 119.8, 111.5, 22.8. HRMS-Q-TOF (*m*/*z*) calcd for C_14_H_10_BrN_3_O_2_S: 363.9755 [M + H]^+^, found: 363.9748.

(*E*)-3-((3,5-Dibromo-2-hydroxybenzylidene)amino)-2-methylthieno[3,2-*d*]pyrimidin-4(3*H*)-one **(13)**

Yellow solid, yield: %86. FTIR (cm^−1^): ν_max_ 3212, 3069, 1668, 1597, 1278. ^1^H NMR (400 MHz, DMSO) δ 11.22 (s, 1H, OH), 9.13 (s, 1H, N=CH), 8.19 (d, *J* = 5.2 Hz, 1H, ArH), 8.00 (s, 1H, ArH), 7.97 (s, 1H, ArH), 7.34 (d, *J* = 5.2 Hz, 1H, ArH), 2.52 (s, 3H, CH_3_). ^13^C NMR (101 MHz, DMSO) δ 169.0, 155.8, 155.2, 155.2, 154.4, 139.3, 136.9, 133.0, 125.6, 121.3, 121.2, 113.3, 111.9, 22.8. HRMS-Q-TOF (*m*/*z*) calcd for C_14_H_9_Br_2_N_3_O_2_S: 441.8860 [M + H]^+^, found: 443.8832 (^81^Br).

(*E*)-3-((3,5-Dibromo-4-hydroxybenzylidene)amino)-2-methylthieno[3,2-*d*]pyrimidin-4(3*H*)-one **(14)**

White solid, yield: %94. FTIR (cm^−1^): ν_max_ 3306, 3097, 1671, 1292. ^1^H NMR (400 MHz, DMSO) δ 8.78 (s, 1H, N=CH), 8.16 (d, *J* = 5.3 Hz, 1H, ArH), 8.11 (s, 2H, ArH), 7.33 (d, *J* = 5.3 Hz, 1H, ArH), 2.48 (s, 3H, CH_3_). ^13^C NMR (101 MHz, DMSO) δ 167.6, 155.8, 155.4, 155.3, 154.6, 136.6, 133.3, 126.9, 125.5, 121.3, 112.8, 22.7. HRMS-Q-TOF (*m*/*z*) calcd for C_14_H_9_Br_2_N_3_O_2_S: 441.8860 [M + H]^+^, found: 443.8840 (^81^Br).

(*E*)-3-((4-Hydroxy-3-nitrobenzylidene)amino)-2-methylthieno[3,2-*d*]pyrimidin-4(3*H*)-one **(15)**

Yellow solid, yield: %87. FTIR (cm^−1^): ν_max_ 3261, 3097, 1665, 1603, 1534, 1374, 1250. ^1^H NMR (400 MHz, DMSO) δ 11.98 (s, 1H, OH), 8.89 (s, 1H, N=CH), 8.42 (s, 1H, ArH), 8.17 (d, *J* = 4.3 Hz, 1H, ArH), 8.12 (d, *J* = 8.7 Hz, 1H, ArH), 7.34 (d, *J* = 4.8 Hz, 1H, ArH), 7.28 (d, *J* = 8.5 Hz, 1H, ArH), 2.48 (s, 3H, CH_3_). ^13^C NMR (101 MHz, DMSO) δ 168.5, 156.2, 155.8, 155.3, 154.6, 138.0, 136.6, 134.8, 127.4, 125.5, 124.0, 121.4, 120.6, 22.7. HRMS-Q-TOF (*m*/*z*) calcd for C_14_H_10_N_4_O_4_S: 331.0501 [M + H]^+^, found: 331.0499.

(*E*)-3-((5-Hydroxy-2-nitrobenzylidene)amino)-2-methylthieno[3,2-*d*]pyrimidin-4(3*H*)-one **(16)**

Yellow solid, yield: %91. FTIR (cm^−1^): ν_max_ 3099, 1663, 1607, 1569, 1369, 1306. ^1^H NMR (400 MHz, DMSO) δ 11.37 (s, 1H, OH), 9.41 (s, 1H, N=CH), 8.18–8.13 (m, 2H, ArH), 7.43 (d, *J* = 2.4 Hz, 1H, ArH), 7.34 (d, *J* = 5.2 Hz, 1H, ArH), 7.12 (dd, *J* = 9.0, 2.5 Hz, 1H, ArH), 2.51 (s, 3H, CH_3_). ^13^C NMR (101 MHz, DMSO) δ 167.9, 163.4, 155.8, 155.1, 154.6, 140.7, 136.7, 130.9, 128.8, 125.5, 121.6, 119.4, 115.9, 22.7. HRMS-Q-TOF (*m*/*z*) calcd for C_14_H_10_N_4_O_4_S: 331.0501 [M + H]^+^, found: 331.0499.

(*E*)-3-((4-Hydroxy-3-methoxy-5-nitrobenzylidene)amino)-2-methylthieno[3,2-*d*]pyrimidin-4(3*H*)-one **(17)**

Yellow solid, yield: %88. FTIR (cm^−1^): ν_max_ 3230, 3089, 1664, 1612, 1541, 1352, 1267. ^1^H NMR (400 MHz, DMSO) δ 8.86 (s, 1H, N=CH), 8.16 (d, *J* = 5.2 Hz, 1H, ArH), 7.99 (s, 1H, ArH), 7.78 (s, 1H, ArH), 7.32 (d, *J* = 5.2 Hz, 1H, ArH), 3.96 (s, 3H, OCH_3_), 2.50 (s, 3H, CH_3_). ^13^C NMR (101 MHz, DMSO) δ 168.6, 155.8, 155.3, 154.6, 150.7, 147.1, 137.7, 136.5, 125.5, 123.1, 121.3, 119.8, 113.4, 57.4, 22.7. HRMS-Q-TOF (*m*/*z*) calcd for C_15_H_12_N_4_O_5_S: 361.0607 [M + H]^+^, found: 361.0609.

(*E*)-3-((4-Hydroxy-3,5-dimethoxybenzylidene)amino)-2-methylthieno[3,2-*d*]pyrimidin-4(3*H*)-one **(18)**

White solid, yield: %92. FTIR (cm^−1^): ν_max_ 3110, 1669, 1608, 1249. ^1^H NMR (400 MHz, DMSO) δ 9.43 (s, 1H, OH), 8.66 (s, 1H, N=CH), 8.14 (d, *J* = 5.1 Hz, 1H, ArH), 7.32 (d, *J* = 5.1 Hz, 1H, ArH), 7.23 (s, 2H, ArH), 3.82 (s, 6H, 2xOCH_3_), 2.47 (s, 3H, CH_3_). ^13^C NMR (101 MHz, DMSO) δ 170.6, 155.8, 155.3, 154.8, 148.8, 141.2, 136.2, 125.5, 122.8, 121.4, 107.2, 56.7, 22.7. HRMS-Q-TOF (*m*/*z*) calcd for C_16_H_15_N_3_O_4_S: 346.0862 [M + H]^+^, found: 346.0861.

(*E*)-3-((3-Ethoxy-2-hydroxybenzylidene)amino)-2-methylthieno[3,2-*d*]pyrimidin-4(3*H*)-one **(19)**

Yellow solid, yield: %89. FTIR (cm^−1^): ν_max_ 3131, 3083, 1678, 1597, 1245. ^1^H NMR (400 MHz, DMSO) δ 9.89 (s, 1H, OH), 9.10 (s, 1H, N=CH), 8.16 (d, *J* = 5.3 Hz, 1H, ArH), 7.45 (d, *J* = 7.9 Hz, 1H, ArH), 7.34 (d, *J* = 5.3 Hz, 1H, ArH), 7.16 (d, *J* = 8.0 Hz, 1H, ArH), 6.89 (t, *J* = 7.9 Hz, 1H, ArH), 4.09 (q, *J* = 6.9 Hz, 2H, OCH_2_CH_3_), 2.48 (s, 3H, CH_3_), 1.35 (t, *J* = 6.9 Hz, 3H, OCH_2_CH_3_). ^13^C NMR (101 MHz, DMSO) δ 168.2, 155.8, 155.3, 154.7, 149.4, 148.0, 136.4, 125.5, 121.5, 120.2, 120.1, 118.9, 117.6, 65.0, 22.8, 15.3. HRMS-Q-TOF (*m*/*z*) calcd for C_16_H_15_N_3_O_3_S: 330.0912 [M + H]^+^, found: 330.0914.

(*E*)-3-((3-Ethoxy-4-hydroxybenzylidene)amino)-2-methylthieno[3,2-*d*]pyrimidin-4(3*H*)-one **(20)**

White solid, yield: %86. FTIR (cm^−1^): ν_max_ 3061, 1680, 1263. ^1^H NMR (400 MHz, DMSO) δ 9.98 (s, 1H, OH), 8.65 (s, 1H, N=CH), 8.15 (d, *J* = 5.2 Hz, 1H, ArH), 7.50 (s, 1H, ArH), 7.33–7.31 (m, 2H, ArH), 6.93 (d, *J* = 8.1 Hz, 1H, ArH), 4.07 (q, *J* = 6.8 Hz, 2H, OCH_2_CH_3_), 2.46 (s, 3H, CH_3_), 1.35 (t, *J* = 6.9 Hz, 3H, OCH_2_CH_3_). ^13^C NMR (101 MHz, DMSO) δ 170.3, 155.6, 155.1, 154.5, 152.2, 147.7, 136.0, 125.2, 125.0, 123.8, 121.2, 116.1, 112.1, 64.3, 22.4, 15.1. HRMS-Q-TOF (*m*/*z*) calcd for C_16_H_15_N_3_O_3_S: 330.0912 [M + H]^+^, found: 330.0914.

### 2.2. In Vitro Antileishmanial Activity Assay

#### 2.2.1. *L. major* Isolates

The *L. major* isolates (MHOM/TR/2014/CBCL-LM) used in the study were obtained from the Parasite Bank at Manisa Celal Bayar University, Faculty of Medicine, Department of Parasitology.

#### 2.2.2. Reproduction of *L. major* in Culture

*L. major* promastigotes cryopreserved in liquid nitrogen (−196 °C) were retrieved from storage and rapidly thawed in a 37 °C water bath. Following thawing, the parasites were aseptically inoculated into Novy–MacNeal–Nicolle (NNN) medium and RPMI-1640 culture medium supplemented with 10% fetal bovine serum (FBS), 1.1% gentamicin, and 1.1% penicillin–streptomycin, and subsequently incubated at 27 °C. To obtain parasites in the logarithmic growth phase, approximately 0.5–1 mL of actively growing promastigote culture was transferred on day 10 into sterile culture flasks containing 5 mL of fresh RPMI-1640 medium and further incubated at 27 °C. During continued cultivation, an additional 2–3 mL of RPMI-1640 medium was supplemented every two days to support parasite proliferation. Parasite growth and development were monitored between days 10 and 20 by enumeration using a Thoma counting chamber. Under these culture conditions, promastigotes reached a density of approximately 1 × 10^8^ parasites/mL by day 20 [20,21].

#### 2.2.3. Antipromastigote Activity for *L. major*

Compounds **1–20** were initially dissolved in dimethyl sulfoxide (DMSO; Sigma-Aldrich, St. Louis, MO, USA) to obtain stock solutions with a final concentration of 200 mM. Serial dilutions of the compounds (3.125–200 µM) or the reference drug miltefosine (Abmole Bioscience, Houston, TX, USA; 2–64 µM) were prepared and added to logarithmic-phase *L. major* parasite suspensions in sterile tubes. The treated parasite cultures were incubated at 27 °C for 24 h. Following incubation, the parasite suspensions were gently vortexed for 5 s, and 100 µL aliquots from each sample were transferred into sterile 96-well plates. Cell viability was then assessed by adding 10 µL of CVDK-8 Cell Viability Kit (EcoTech, Ankara, Türkiye) to each well, followed by incubation at 37 °C for 60 min. Absorbance was measured at 450 nm using a microplate spectrophotometer (Thermo Scientific, Waltham, MA, USA) [18].

#### 2.2.4. Cytotoxicity

To assess the cytotoxic effects of compounds **5**, **12**, and **19** in comparison with miltefosine, human umbilical vein endothelial cells (HUVECs; ATCC, Manassas, VA, USA) were employed as a model of healthy cells. HUVECs were maintained in Dulbecco’s Modified Eagle Medium (DMEM) supplemented with 10% fetal bovine serum (FBS; Gibco, Waltham, MA, USA) and 1% penicillin–streptomycin solution (Gibco, USA) at 37 °C under a humidified atmosphere containing 5% CO_2_. Cells were harvested using trypsin and seeded into sterile 96-well plates at a density of 4 × 10^3^ cells per well. After a 24 h incubation period to allow cell attachment, the culture medium was replaced with fresh medium containing compounds **5**, **12**, and **19** or miltefosine at final concentrations ranging from 3.125 to 200 µM (3.125, 6.25, 12.5, 25, 50, 100, and 200 µM; n = 3). The treated cells were incubated for an additional 24 h. Cell viability was subsequently evaluated by adding 10 µL of CVDK-8 Cell Viability Kit (EcoTech) to each well, followed by incubation at 37 °C for 60 min. Absorbance values were recorded at 450 nm using a microplate spectrophotometer [18].

#### 2.2.5. IC_50_ Calculations

Absorbance values obtained from the cell viability assays were normalized to the control group, which was defined as 100%, and expressed as percentage viability. The processed data were analyzed using GraphPad Prism version 10.6.1. IC_50_ values were calculated by nonlinear regression using a variable slope (four-parameter logistic) dose–response model with 95% confidence intervals. Results are presented as dose–response curves, and IC_50_ values are expressed as mean ± standard deviation.

### 2.3. Molecular Docking

Molecular docking studies were carried out using the Schrödinger Molecular Modeling Suite (2025-1) via the Maestro graphical interface (v14.3). The three-dimensional crystallographic structures of the target proteins (pteridine reductase (PDB ID: 1E7W), dihydroorotate dehydrogenase (PDB ID: 3GYE), nucleoside diphosphate kinase B (PDB ID: 3NGT), methionyl-tRNA synthetase (PDB ID: 3KFL), and phosphodiesterase B1 (PDB ID: 2RQ8) were retrieved from the Protein Data Bank. Protein and ligand preparation steps were performed according to established protocols, and protein structures were optimized using the Protein Preparation Wizard to ensure appropriate protonation states, hydrogen-bonding networks, and geometric integrity prior to docking. To account for receptor flexibility and achieve more reliable binding predictions, induced-fit docking (IFD) was employed. For each ligand, 20 binding poses were generated within the defined active site, and docking calculations were performed using the Glide XP (extra precision) algorithm. The resulting poses were ranked based on their docking scores, and the most favorable binding conformations were selected for further analysis. To complement the docking results and provide a more quantitative assessment of binding affinity, Prime MM-GBSA calculations were conducted on the selected protein–ligand complexes. Binding free energies (ΔG_bind) were estimated using the VSGB solvation model, allowing for a refined evaluation of the stability and strength of the predicted interactions [22,23,24].

## 3. Results and Discussion

### 3.1. Chemistry

In this study, twenty thieno[3,2-*d*]pyrimidine derivative Schiff bases were synthesized to evaluate their antipromastigote activities against *L. major*. Methyl 3-aminothiophene-2-carboxylate was used as the starting material in the synthesis process, and the intermediate 3-amino-2-methylthieno[3,2-*d*]pyrimidine-4(3*H*)-one **(TPM)** was obtained with 90% yield via a two-step reaction sequence [13]. In the second step, the target Schiff base compounds **(1–20)** were successfully synthesized via a condensation reaction of TPM with various commercial phenolic aldehydes, and the products were isolated with good yields in the range of 85–94% (Figure 2) [25]. The structures of all synthesized compounds were confirmed using FTIR, NMR, and HRMS spectroscopic techniques.

In the FTIR spectrum of compounds **1–20**, the C=O stretching bands of the thienopyrimidine ring and the characteristic N=CH bands of the imine group were seen in the range of 1685–1647 cm^−1^ and 1612–1597 cm^−1^, respectively [13]. The singlet observed at δ 4.99 ppm in the ^1^H NMR spectrum of the TPM compound belongs to the NH_2_ group, and as expected, this signal was not observed in the spectrum of the target Schiff base compounds [13]. Examination of the ^1^H NMR spectra of the target compounds reveals that the signals corresponding to the phenolic OH protons resonate as broad singlets in the range of δ 11.98–8.74 ppm, depending on the position of the hydroxyl group in the aromatic ring [26]. Additionally, proton signals belonging to the imine group, confirming Schiff base formation, were observed as singlets in the range of δ 9.41–8.60 ppm, again depending on the substitution pattern [27]. The methyl protons at position 2 of the thienopyrimidine core were detected as singlets in the range of δ 2.52–2.44 ppm in all target compounds. In ^13^C NMR spectra, signals corresponding to N=CH carbons, supporting imine formation, were observed in the range of δ 175.3–165.4 ppm [28]. Furthermore, resonances corresponding to C=O carbons were detected in the range of δ 155.8–155.2 ppm [13]. Methyl carbons at position 2 of the thienopyrimidine ring gave signals in the range of δ 22.8–22.0 ppm in all target compounds.

### 3.2. In Vitro Antipromastigote Activity and Cytotoxicity Assay

**Figure 3:**
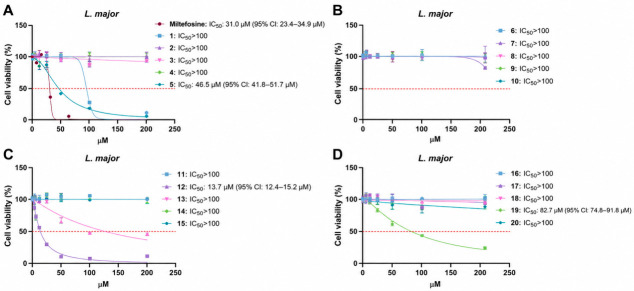
In vitro antipromastigote activity of compounds **1–20** against *L. major* ((**A**) compounds **1–5** and miltefosine, (**B**) compounds **6–10**, (**C**) compounds **11–15**, (**D**) compounds **16–20**). IC_50_ values were determined from dose–response curves and are expressed as mean ± standard deviation.

According to the results obtained, most of the synthesized compounds exhibited IC_50_ values above 100 µM against *L. major* promastigotes after a 24-h incubation period (Figure 3). However, compounds **5**, **12**, and **19** showed significant antipromastigote activity. The 24-h IC_50_ values of these compounds against *L. major* were calculated as 46.5 µM (95% CI: 41.8–51.7 µM), 13.7 µM (95% CI: 12.4–15.2 µM), and 82.7 µM (95% CI: 74.8–91.8 µM), respectively. The reference drug miltefosine showed an IC_50_ value of 31.0 µM under the same conditions.

**Figure 4:**
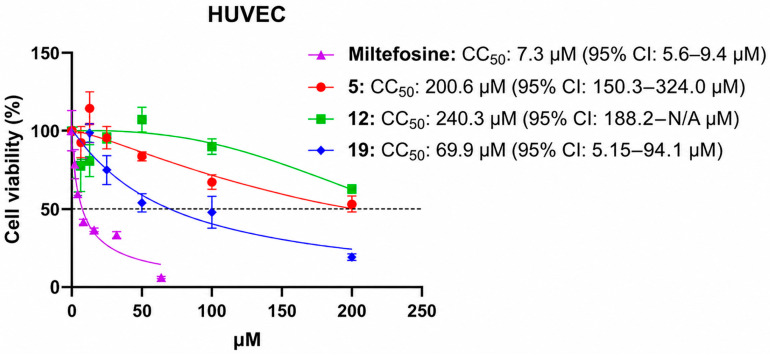
Cytotoxic effects of compounds **5**, **12**, and **19**, and miltefosine on HUVECs. CC_50_ values were calculated from dose–response curves and are presented as mean ± standard deviation. N/A denotes “Not Applicable”, indicating that the corresponding parameter was not applicable under the experimental conditions and was therefore not evaluated. The symbols used in the graph, including squares, triangles and circles, represent the dose–cell viability data points for the respective compounds and are used consistently throughout the figure.

To evaluate the cytotoxic effects of the selected active compounds on host cells, the HUVEC line was used as a non-cancerous cellular model. The 24-h CC_50_ values of compounds **5**, **12**, **19**, and miltefosine on HUVECs were determined to be 200.6 µM, 240.3 µM, 69.9 µM, and 7.3 µM, respectively (Figure 4).

Based on these data, the selectivity index (SI) values for the active compounds and miltefosine were calculated and are presented in Table 1. The SI values for compounds **5**, **12**, and **19** were found to be 4.3, 17.5, and 0.8, respectively, while this value was calculated as 0.2 for miltefosine.

Among the synthesized compounds, **12** stood out due to both its antipromastigote activity and its low cytotoxic effect on host cells. Exhibiting an IC_50_ value of 13.7 µM against *L. major*, **12** showed comparable activity to the reference drug miltefosine, while its high IC_50_ value (240.3 µM) on HUVECs demonstrated a distinct selectivity profile. The calculated SI value (17.5) indicates that **12** has the most favorable in vitro therapeutic window among the compounds evaluated in this study. These findings suggest that **12** is a suitable candidate for further biological and mechanistic studies.

#### Structure–Activity Relationship (SAR) Analysis

It appears that the activity of the synthesized thieno[3,2-*d*]pyrimidine derivative Schiff bases is closely related to the phenolic substitution pattern in the aromatic ring. Particularly noteworthy is the relatively better antipromastigote activity exhibited by compounds derived from salicylaldehydes, those with the phenolic hydroxyl group in the ortho (2-OH) position (**5**, **12**, and **19**). This can be attributed to the ortho-hydroxyl group forming intramolecular hydrogen bonds with the imine nitrogen, resulting in a more rigid and conformationally advantageous structure, thus offering a more suitable geometry for interaction with the target protein.

When derivatives with single hydroxyl substitutions were examined, compounds containing hydroxyls in the ortho, para, or meta positions **(1–3)** showed weak activity (IC_50_ > 100 µM). Among compounds containing multiple hydroxyl groups, only **5** (2,4-diOH) exhibited moderate activity (IC_50_ = 46.5 µM), while other derivatives showed no significant effect. In particular, the close proximity of hydroxyl groups in compounds **4** (2,3-diOH), **7** (3,4-diOH), and **8** (2,3,4-triOH) may have led to intramolecular hydrogen bonding between these groups, limiting their participation in the interaction with the target protein. In contrast, the more distant positioning of hydroxyl groups on the aromatic ring in compound **5** may have reduced such intramolecular interactions and allowed for the relative preservation of biological activity. On the other hand, the lack of activity observed in compound **6** (2,5-diOH), despite the distant positioning of hydroxyl groups, suggests that this positioning does not provide an interaction geometry compatible with the target protein’s binding site.

In methoxy compounds derived from vanillin, isovaniline, and syringaldehyde **(9, 10,** and **18)**, similar intramolecular interactions are likely to occur due to the presence of methoxy groups adjacent to the phenolic hydroxyl group. This may have reduced the effectiveness of the hydroxyl group as a hydrogen bond donor, leading to decreased biological activity.

When the effect of halogen substitution was evaluated, compound **12** (2-OH-5-Br) emerged as the most active compound in the series. This suggests that the presence of a single bromine atom at the 5-position is well tolerated and may contribute positively to activity, possibly through favorable modulation of lipophilicity and enhanced hydrophobic interactions within the binding pocket, while preserving the key conformational role of the ortho-hydroxyl group. In contrast, the activity of compound **11** (3-Br-4-OH) was negligible, which may indicate that the substitution pattern does not support an optimal spatial arrangement for effective target interaction. Furthermore, dibrominated derivatives **(13** and **14)** showed markedly reduced activity, which can likely be attributed to steric effects arising from the additional bromine atoms. These bulky substituents may interfere with the proper orientation of the phenolic hydroxyl group and consequently weaken its ability to participate in key hydrogen-bonding interactions with the target site.

In derivatives containing a nitro group **(15–17)**, no significant antileishmanial activity was observed. This can be explained by the nitro group reducing the electron density of the aromatic ring and simultaneously limiting the interaction of the hydroxyl group with the target by forming intramolecular hydrogen bonds with the phenolic hydroxyl group.

Finally, when derivatives with ethoxy substitution were examined, it was determined that **19**, containing a hydroxyl at the 2-OH position, showed activity, albeit at a low level (IC_50_ = 82.7 µM), while **20**, containing a 4-OH, showed no activity (IC_50_ > 100 µM). These results support the idea that the ortho-hydroxyl position plays a critical role in antileishmanial activity, even with different substituents.

### 3.3. Molecular Docking

To gain preliminary insight into the possible molecular interactions underlying the observed antipromastigote activity, molecular docking studies were performed to predict the possible mechanism of action of the most active compound **(12)** against selected *L. major* target proteins. Several *L. major* proteins have been reported as potential therapeutic targets in antileishmanial drug discovery due to their essential roles in parasite survival, proliferation, and virulence [29]. In this context, enzymes involved in parasite metabolic pathways and cytoskeletal organization are frequently investigated as drug targets, as their inhibition may lead to impaired growth or cell death of the parasite.

Firstly, the aim of molecular docking analyses in this study is not to define a precise mechanism of action for antileishmanial activity, but to provide a predictive mechanistic framework by comparatively examining the binding affinities and interaction modes of the most active compound in the series with potential pivot proteins that may play a role in its efficacy against *L. major*. To this end, compound **12** was docked to pteridine reductase, dihydroorotate dehydrogenase, nucleoside diphosphate kinase B, methionyl-tRNA synthetase, and phosphodiesterase B1 proteins isolated from *L. major* [18,29]. Docking studies were performed using the Induced Fit Docking (IFD) protocol, which allows for more realistic binding poses compared to classical rigid docking approaches due to its simultaneous consideration of ligand and protein flexibility [30]. The IFD approach provides more accurate modeling of binding geometry and interaction networks, especially in systems where the active site conformation can change depending on ligand binding. The optimal protein-ligand complexes obtained were re-scored using the MM-GBSA method to quantitatively evaluate their binding free energies [31]. The results obtained from IFD and MM-GBSA analyses are summarized in Table 2.

Examination of the docking results presented in Table 2 shows that compound **12** exhibits varying binding affinities to proteins associated with different biological pathways of *L. major*. Among the targets examined, phosphodiesterase B1 (PDEB1) stands out as the protein to which the compound showed the highest binding affinity. The IFD score (−9.042 kcal/mol) and MM-GBSA binding free energy (−67.21 kcal/mol) obtained for PDEB1 indicate that compound **12** can bind strongly and stably to the active site of this enzyme. This finding is biologically noteworthy, as PDEB1 is a target protein that plays a critical role in the regulation of intracellular cAMP signaling in Leishmania species and is considered important for the proliferation and life cycle of the parasite [32,33].

Compound **12** also shows moderate-to-low interaction with methionyl-tRNA synthetase (MetRS). The calculated IFD score of −6.724 kcal/mol and MM-GBSA value of −40.81 kcal/mol for MetRS indicate that the compound has a low binding potential to this enzyme, which plays a key role in protein synthesis. MetRS is a vital enzyme in *Leishmania species* and is one of the targets widely evaluated in antiparasitic drug development studies [34,35].

In contrast, the binding energies obtained for dihydroorotate dehydrogenase (DHODH) [36] and nucleoside diphosphate kinase B (NDKB) [37] are low, suggesting that compound 12 has a relatively limited interaction potential with these proteins. The weaker binding scores observed for pteridine reductase (PTR1) suggest that compound **12** may be less likely to exert a dominant effect through this enzyme involved in folate metabolism [38,39].

When these results are considered together, it can be predicted that the observed antipromastigote activity of compound **12** against *L. major* may not be due to a single target protein, but rather to the contribution of its interactions with proteins that play a critical role in the parasite’s vital processes, particularly PDEB1 and MetRS. However, it should be noted that the obtained docking data are predictive in nature and need to be supported by experimental enzyme inhibition studies.

**Figure 5:**
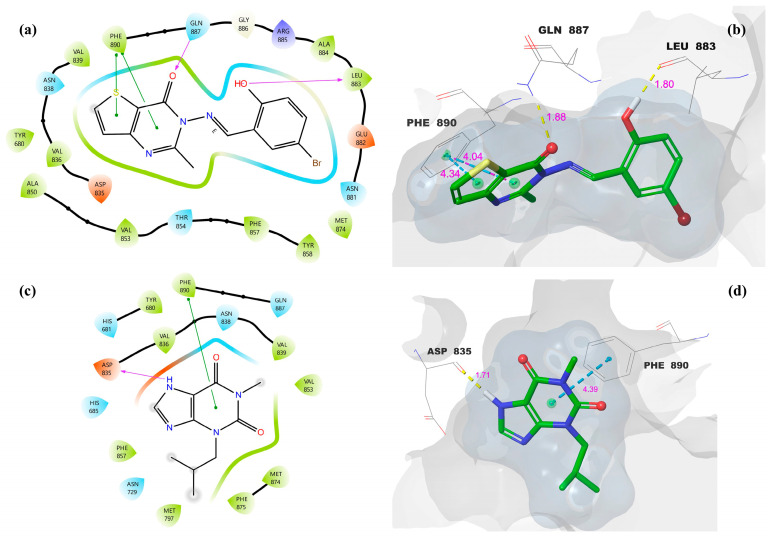
Molecular docking 2D (**a**) and 3D (**b**) ligand-protein interactions of 12-PDEB1 complex and 2D (**c**) and 3D (**d**) ligand-protein interactions of IBMX-PDEB1 complex.

The binding mode of compound **12** and 3-isobutyl-1-methyl-3,7-dihydro-1*H*-purine-2,6-dione (known as IBMX), the reference inhibitor of PDEB1, to the active site of the *L. major* PDEB1 enzyme is shown in Figure 5. Docking results reveal that the compound adopts a stable binding position by forming multiple and directed interactions in the PDEB1 active site. The formation of a hydrogen bond between the phenolic OH group and the Leu883 residue at a distance of 1.80 Å indicates that the compound is oriented correctly towards the active site. The fact that Leu883 is one of the key residues shaping the hydrophobic character of the ligand-binding pocket of PDEB1 enhances the contribution of this interaction to binding stability [40].

The carbonyl oxygen of the thieno[3,2-*d*]pyrimidine ring contributes to the stabilization of the compound’s binding conformation by forming a hydrogen bond with Gln887, located near the entrance of the active site, at a distance of 1.88 Å. The fact that Gln887 is a polar residue involved in ligand recognition and orientation supports the importance of this interaction in terms of binding specificity [18,41]. Furthermore, the aromatic structure of the thienopyrimidine ring strengthens the hydrophobic interaction network by forming two π–π stacking interactions (4.04 and 4.34 Å) with the Phe890 residue. This interaction refers to noncovalent interactions occurring between aromatic systems, which arise from a combination of dispersion forces, electrostatic interactions, and other weak intermolecular contributions rather than a specific π-orbital-driven attractive force. These interactions are important in medicinal chemistry as they can contribute to ligand stabilization within hydrophobic or aromatic-rich binding pockets of target proteins, thereby influencing binding affinity and overall biological activity [42]. Phe890 is known to play a critical role in the binding of aromatic ligands in the PDEB1 active site [43], and these interactions can be considered one of the key factors explaining the high binding affinity of compound **12**.

In addition, the polar interactions of the bromine atom with Asn881, the phenolic OH group with Gly886, and the sulfur atom in the thienopyrimidine ring with Phe890 further enhance the overall stability of the ligand–protein complex. This multi-interaction network enables compound **12** to bind strongly and in a well-defined manner to the PDEB1 active site, presenting a picture consistent with the high binding affinity values obtained in docking studies.

To compare the binding profile of compound **12**, the interactions of the reference inhibitor IBMX with the *L. major* PDEB1 protein were also investigated. IBMX exhibited a docking score of −6.481 kcal/mol and an MM-GBSA binding energy of −17.74 kcal/mol. Similar to compound **12**, IBMX formed a π–π stacking interaction with Phe890 at a distance of 4.39 Å. In addition, IBMX established a hydrogen bond interaction with Asp835 (1.71 Å). In contrast, compound **12** displayed a more extensive interaction pattern by forming two additional hydrogen bond interactions with Gln887 and Leu883 at distances of 1.88 and 1.80 Å, respectively, together with an additional π–π stacking interaction with Phe890 (4.34 Å). Consistent with these interactions, compound **12** demonstrated markedly improved binding parameters, with a docking score of −9.042 kcal/mol and an MM-GBSA value of −67.21 kcal/mol. These findings suggest that compound **12** possesses a more favorable binding mode toward the PDEB1 active site compared with the reference inhibitor IBMX.

Miltefosine, the reference antileishmanial drug used in this study, is an alkylphosphocholine derivative whose mechanism of action is not fully understood but is primarily associated with disruption of membrane lipid metabolism, inhibition of phospholipid and sterol biosynthesis, immunomodulation, and induction of apoptosis-like cell death in Leishmania parasites [44,45]. Since no study associating miltefosine with *L. major* PDEB1 inhibition has been reported to date, a comparative PDEB1 inhibition analysis against miltefosine was not performed. This provides a relevant pharmacological benchmark for interpreting the docking interactions observed for the investigated compounds.

## 4. Conclusions

In this study, a series of thieno[3,2-*d*]pyrimidine-4(3*H*)-one-based phenolic Schiff bases were synthesized and their antipromastigote activity against *L. major* was systematically evaluated. In vitro biological results revealed that among the synthesized compounds, compound **12** was the most effective and selective derivative of the series, with an IC_50_ value of 13.7 µM and a selectivity index of 17.5. These values indicate a more favorable therapeutic profile compared to the reference drug miltefosine (IC_50_ = 31.0 µM, SI = 0.2). SAR assessments revealed that the activity is largely related to the ortho-hydroxyl substitution in the aromatic ring, and this group plays a critical role in both activity and selectivity.

In computational studies, compound **12**, the most active compound in the series, was docked onto biologically important proteins isolated from *L. major*. IFD and MM-GBSA analyses showed that compound **12** exhibited a particularly strong binding affinity to the PDEB1 protein (IFD: −9.042 kcal/mol, MM-GBSA: −67.21 kcal/mol). Binding energies obtained for other target proteins remained at low levels, suggesting that PDEB1 could be a potential pivot target in the antipromastigote activity of compound **12**.

This study also has some limitations. First, biological evaluations were performed only under in vitro conditions and only on the promastigote form of *L. Major*; therefore, direct generalization of the obtained results to in vivo efficacy and pharmacokinetic behavior is not possible. While the promastigote stage is commonly used as an initial screening model for antileishmanial activity, it does not fully represent the clinically relevant intracellular amastigote form. Similarly, computational docking and free binding energy calculations are predictive approaches, and the proposed interaction modes have not been confirmed by experimental biochemical or structural studies. Therefore, further studies are needed to evaluate the active derivatives, particularly compound **12**, against the amastigote stage, to conduct target validation experiments, and to perform structural optimization studies.

## Figures and Tables

**Table 1:** In vitro cytotoxicity results and selectivity indices of compounds 5, 12, 19, and miltefosine.

Compounds	IC_50_ (µM)	CC_50_ (µM)	Selectivity Index (SI)
	*L. major*	HUVEC	
**5**	46.5	200.6	4.3
**12**	13.7	240.3	17.5
**19**	82.7	69.9	0.8
**Miltefosine**	31.0	7.3	0.2

**Table 2:** Docking scores and MM-GBSA ΔG binding free energies of compound **12** for some *L. major* proteins.

Proteins	PDB ID	Compound 12	
		IFD Scores (kcal/mol)	MM-GBSA ΔG Bind. (kcal/mol)
Pteridine reductase	1E7W	−4.671	−22.01
Dihydroorotate dehydrogenase	3GYE	−4.809	−46.09
Nucleoside diphosphate kinase B	3NGT	−4.999	−29.04
Methionyl-tRNA synthetase	3KFL	−6.724	−40.81
Phosphodiesterase B1	2RQ8	−9.042	−67.21

## Data Availability

The original contributions presented in this study are included in the article/Supplementary Material. Further inquiries can be directed to the corresponding authors.

## Supplementary Materials

The following supporting information can be downloaded at: https://www.mdpi.com/article/10.3390/life16060979/s1.

## Abbreviations

FTIR	Fourier Transform Infrared Spectroscopy
NMR	Nuclear Magnetic Resonance
HRMS	High-Resolution Mass Spectrometry
IFD	Induced Fit Docking
MM-GBSA	Molecular Mechanics–Generalized Born Surface Area
HUVEC	Human Umbilical Vein Endothelial Cell
SAR	Structure–Activity Relationship
PTR1	Pteridine Reductase
DHODH	Dihydroorotate Dehydrogenase
NDKB	Nucleoside Diphosphate Kinase B
MetRS	Methionyl-tRNA Synthetase
PDEB1	Phosphodiesterase B1
NNN	Novy–MacNeal–Nicolle
FBS	Fetal Bovine Serum
DMSO	Dimethyl Sulfoxide
