# Supplementary material for: New Thieno[3,2-d]pyrimidin-4(3H)-one Schiff Bases as Selective Antileishmanial Agents
Source: Life (Basel). 2026 Jun 10;16(6):979. doi: 10.3390/life16060979 (PMC13302256; doi:10.3390/life16060979)
Supplement: Supplementary file 1 [file life-16-00979-s001.zip › life-4291008-supplementary.pdf]

SUPPLEMENTARY MATERIAL

CONTENTS **Page**

^{1}H NMR Spectrum of TPM.......... 3
^{13}C NMR Spectrum of TPM.......... 3
FTIR Spectrum of TPM.......... 4
HRMS Spectrum of TPM.......... 4
^{1}H NMR Spectrum of Compound 1.......... 5
^{13}C NMR Spectrum of Compound 1.......... 5
FTIR Spectrum of Compound 1.......... 6
HRMS Spectrum of Compound 1.......... 6
^{1}H NMR Spectrum of Compound 2.......... 7
^{13}C NMR Spectrum of Compound 2.......... 7
FTIR Spectrum of Compound 2.......... 8
HRMS Spectrum of Compound 2.......... 8
^{1}H NMR Spectrum of Compound 3.......... 9
^{13}C NMR Spectrum of Compound 3.......... 9
FTIR Spectrum of Compound 3.......... 10
HRMS Spectrum of Compound 3.......... 10
^{1}H NMR Spectrum of Compound 4.......... 11
^{13}C NMR Spectrum of Compound 4.......... 11
FTIR Spectrum of Compound 4.......... 12
HRMS Spectrum of Compound 4.......... 12
^{1}H NMR Spectrum of Compound 5.......... 13
^{13}C NMR Spectrum of Compound 5.......... 13
FTIR Spectrum of Compound 5.......... 14
HRMS Spectrum of Compound 5.......... 14
^{1}H NMR Spectrum of Compound 6.......... 15
^{13}C NMR Spectrum of Compound 6.......... 15
FTIR Spectrum of Compound 6.......... 16
HRMS Spectrum of Compound 6.......... 16
^{1}H NMR Spectrum of Compound 7.......... 17
^{13}C NMR Spectrum of Compound 7.......... 17
FTIR Spectrum of Compound 7.......... 18
HRMS Spectrum of Compound 7.......... 18
^{1}H NMR Spectrum of Compound 8.......... 19
^{13}C NMR Spectrum of Compound 8.......... 19
FTIR Spectrum of Compound 8.......... 20
HRMS Spectrum of Compound 8.......... 20
^{1}H NMR Spectrum of Compound 9.......... 21
^{13}C NMR Spectrum of Compound 9.......... 21
FTIR Spectrum of Compound 9.......... 22
HRMS Spectrum of Compound 9.......... 22
^{1}H NMR Spectrum of Compound 10.......... 23
^{13}C NMR Spectrum of Compound 10.......... 23
FTIR Spectrum of Compound 10.......... 24
HRMS Spectrum of Compound 10.......... 24
^{1}H NMR Spectrum of Compound 11.......... 25
^{13}C NMR Spectrum of Compound 11.......... 25
FTIR Spectrum of Compound 11.......... 26
HRMS Spectrum of Compound 11.......... 26
^{1}H NMR Spectrum of Compound 12.......... 27
^{13}C NMR Spectrum of Compound 12.......... 27

FTIR Spectrum of Compound 12………………………………………………………… 28
HRMS Spectrum of Compound 12……………………………………………………….. 28
^{1}H NMR Spectrum of Compound 13………………………………………………….. 29
^{13}C NMR Spectrum of Compound 13…………………………………………………. 29
FTIR Spectrum of Compound 13………………………………………………………… 30
HRMS Spectrum of Compound 13……………………………………………………….. 30
^{1}H NMR Spectrum of Compound 14………………………………………………….. 31
^{13}C NMR Spectrum of Compound 14…………………………………………………. 31
FTIR Spectrum of Compound 14………………………………………………………… 32
HRMS Spectrum of Compound 14……………………………………………………….. 32
^{1}H NMR Spectrum of Compound 15………………………………………………….. 33
^{13}C NMR Spectrum of Compound 15…………………………………………………. 33
FTIR Spectrum of Compound 15………………………………………………………… 34
HRMS Spectrum of Compound 15……………………………………………………….. 34
^{1}H NMR Spectrum of Compound 16………………………………………………….. 35
^{13}C NMR Spectrum of Compound 16…………………………………………………. 35
FTIR Spectrum of Compound 16………………………………………………………… 36
HRMS Spectrum of Compound 16……………………………………………………….. 36
^{1}H NMR Spectrum of Compound 17………………………………………………….. 37
^{13}C NMR Spectrum of Compound 17…………………………………………………. 37
FTIR Spectrum of Compound 17………………………………………………………… 38
HRMS Spectrum of Compound 17……………………………………………………….. 38
^{1}H NMR Spectrum of Compound 18………………………………………………….. 39
^{13}C NMR Spectrum of Compound 18…………………………………………………. 39
FTIR Spectrum of Compound 18………………………………………………………… 40
HRMS Spectrum of Compound 18……………………………………………………….. 40
^{1}H NMR Spectrum of Compound 19………………………………………………….. 41
^{13}C NMR Spectrum of Compound 19…………………………………………………. 41
FTIR Spectrum of Compound 19………………………………………………………… 42
HRMS Spectrum of Compound 19……………………………………………………….. 42
^{1}H NMR Spectrum of Compound 20………………………………………………….. 43
^{13}C NMR Spectrum of Compound 20…………………………………………………. 43
FTIR Spectrum of Compound 20………………………………………………………… 44
HRMS Spectrum of Compound 20……………………………………………………….. 44
2D and 3D Docking Poses of 12-PDEB1 Complex……………………………………… 45

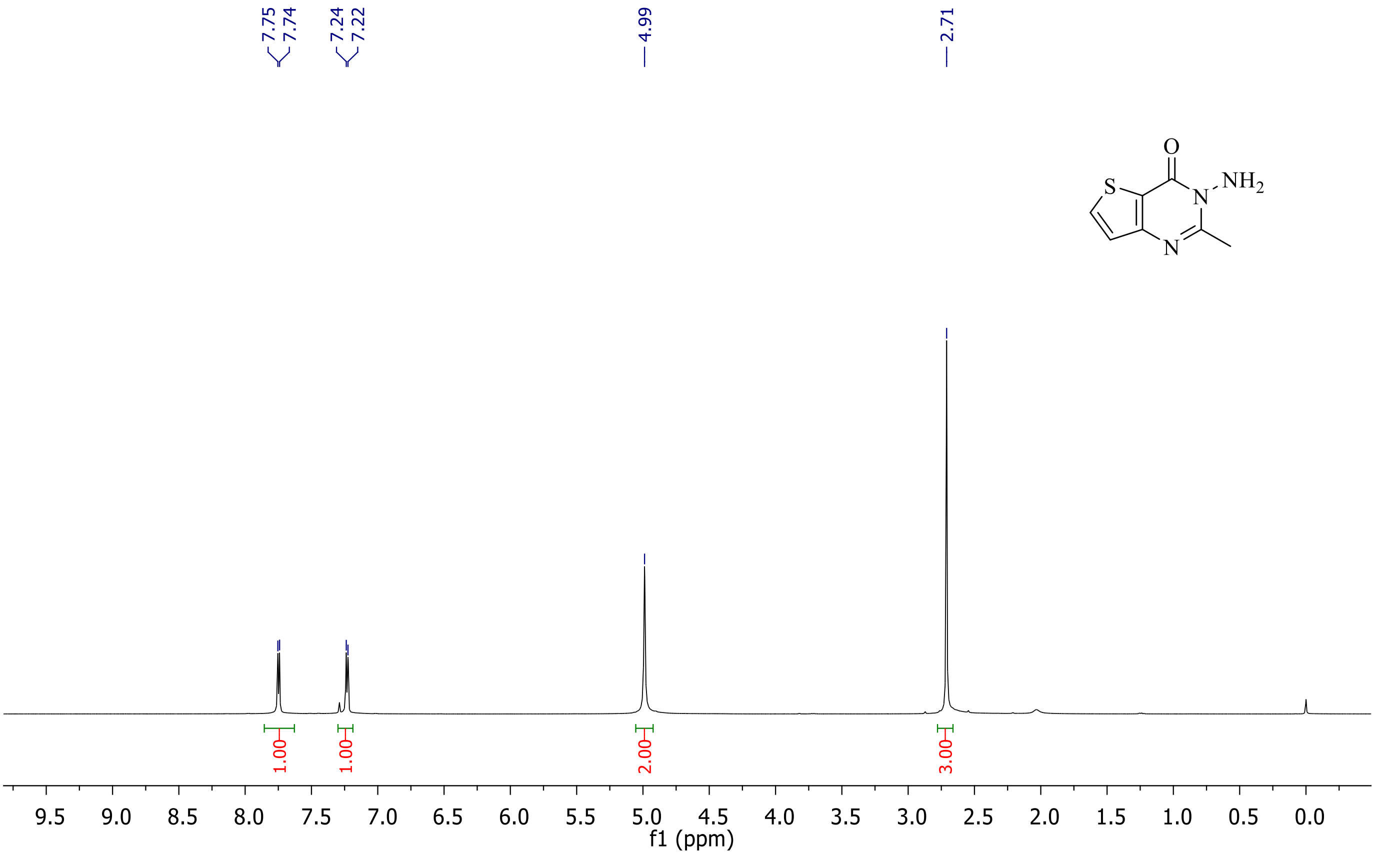

^{1}H NMR Spectrum of TPM

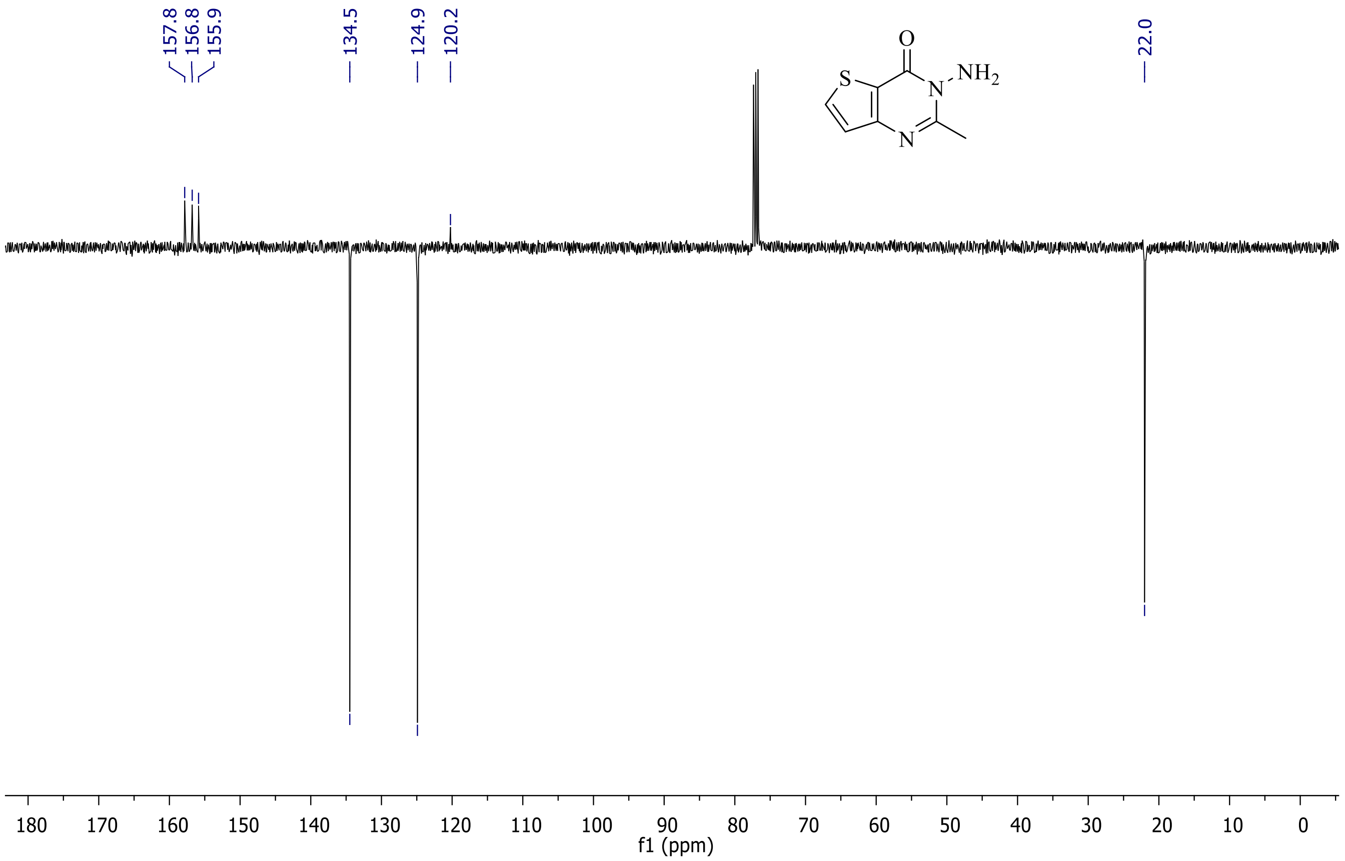

^{13}C NMR-APT Spectrum of TPM

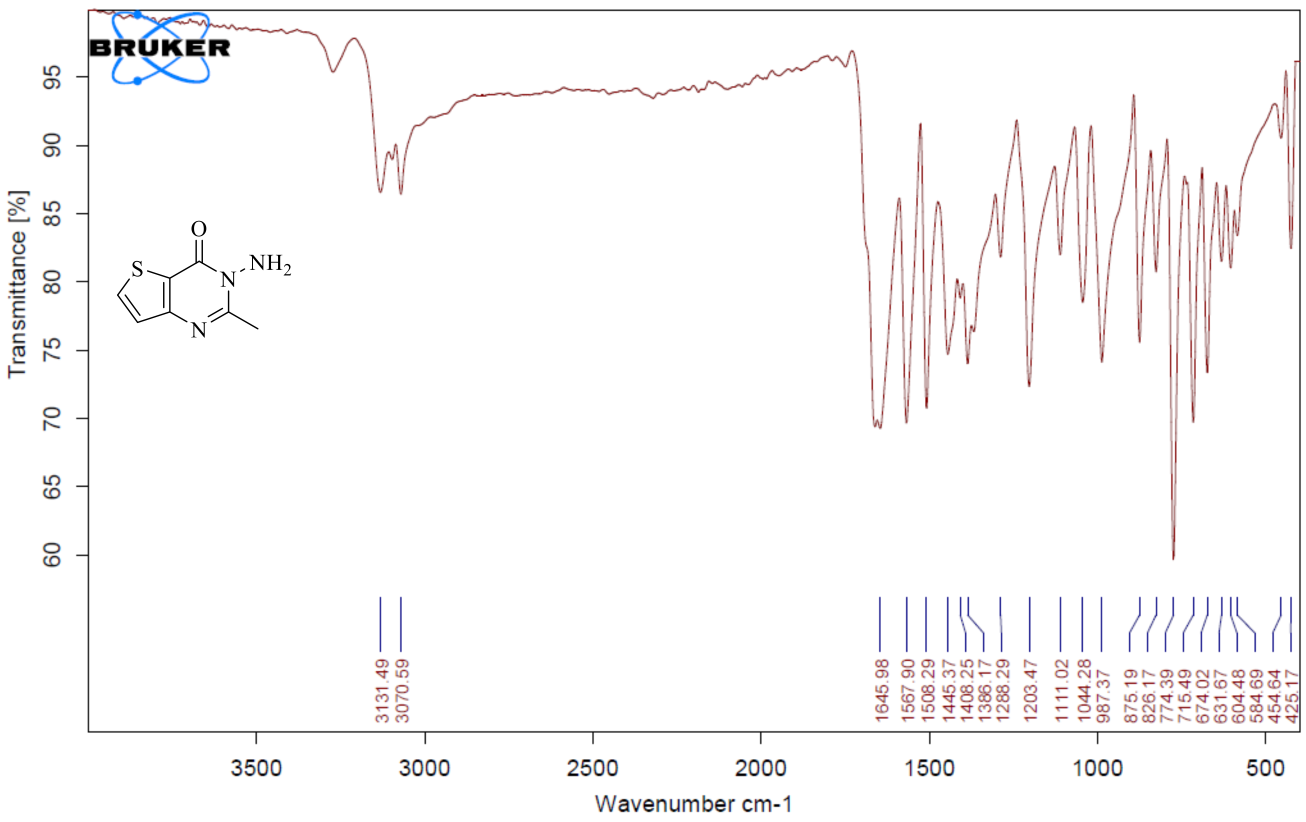

FTIR Spectrum of TPM

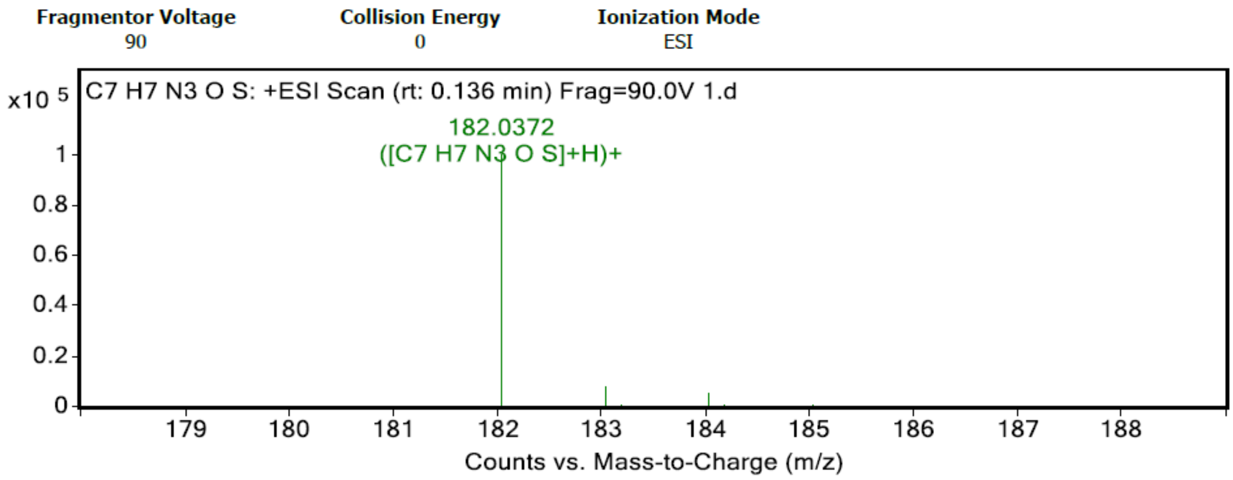

HRMS Spectrum of TPM

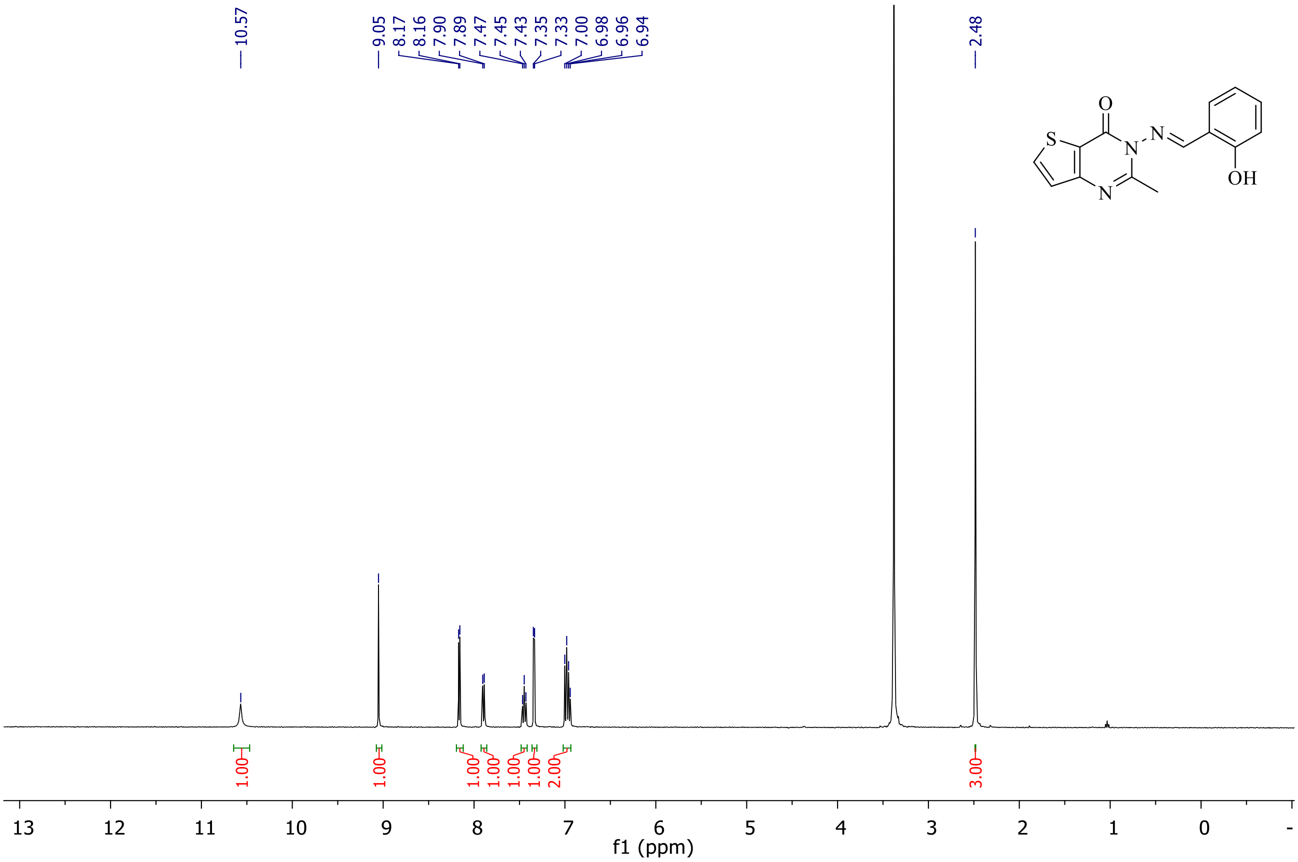

^{1}H NMR Spectrum of Compound 1

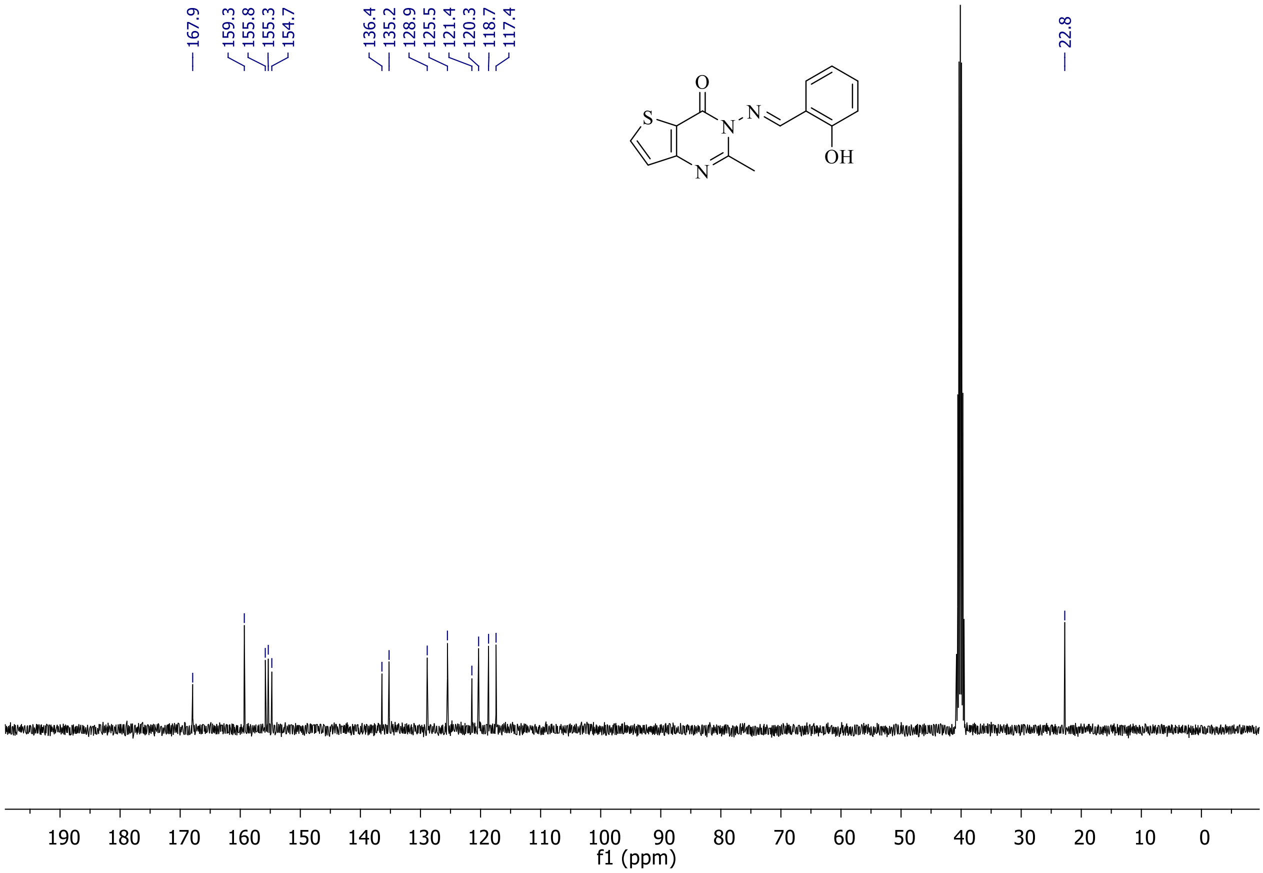

^{13}C NMR Spectrum of Compound 1

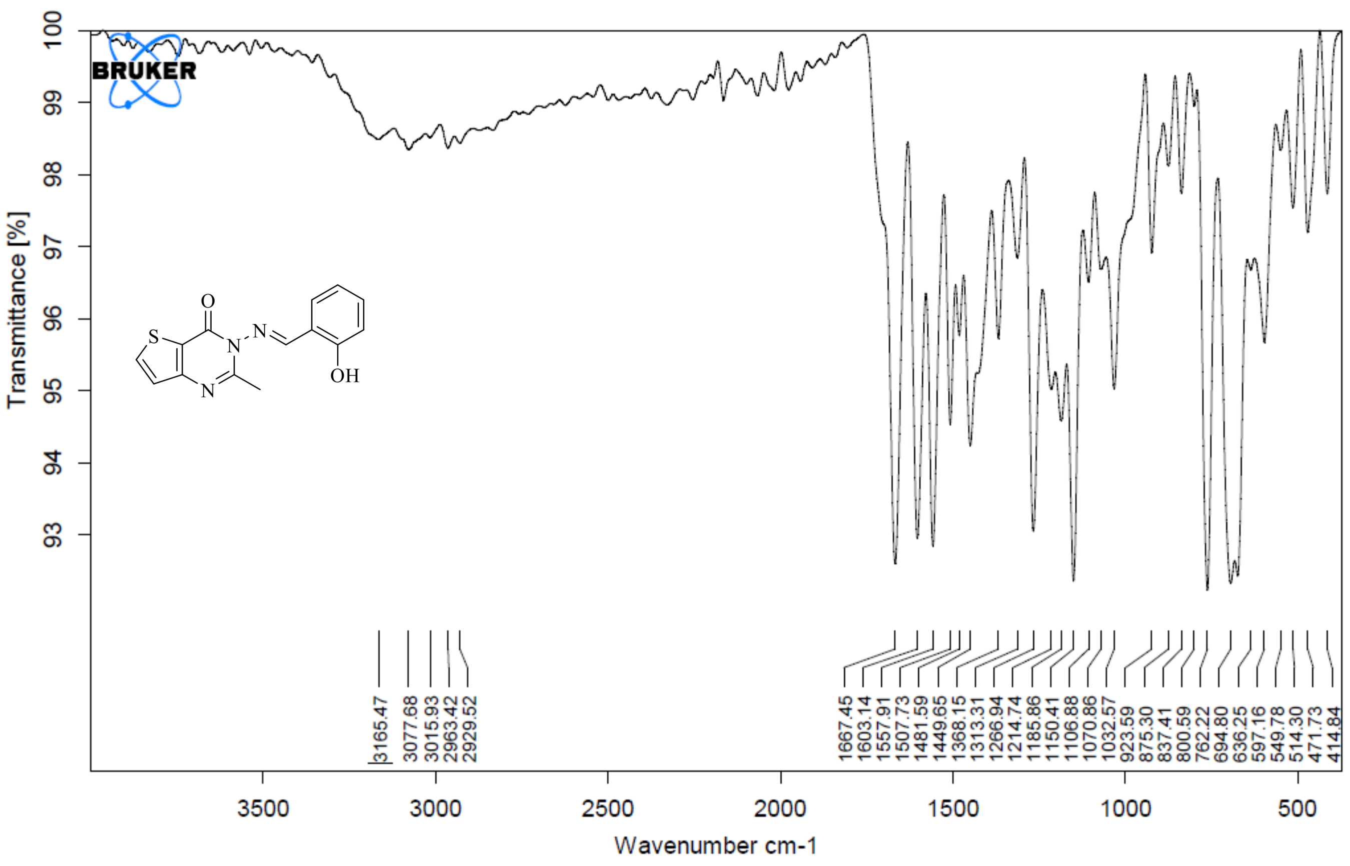

FTIR Spectrum of Compound 1

Spectra

Fragmentor Voltage	Collision Energy	Ionization Mode
90	0	ESI

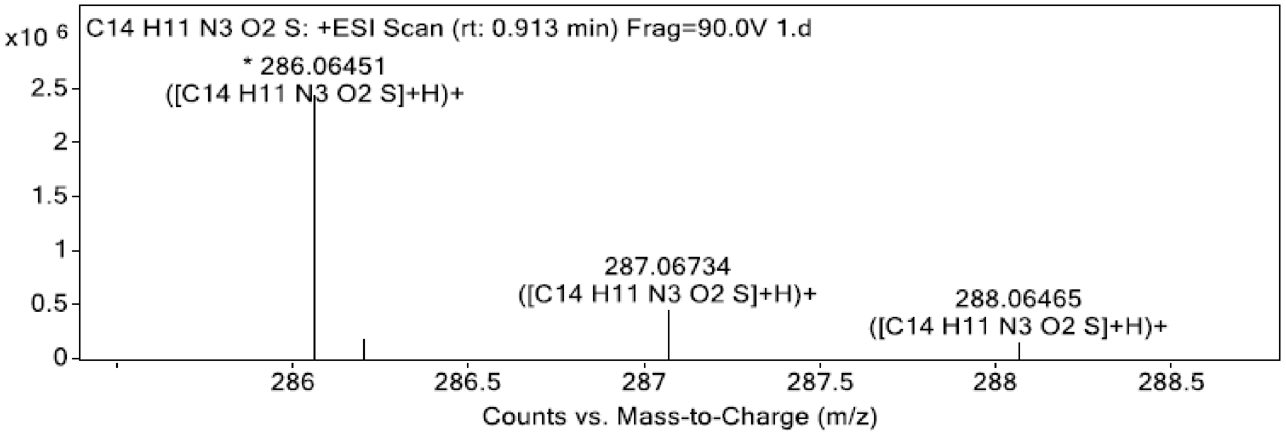

HRMS Spectrum of Compound 1

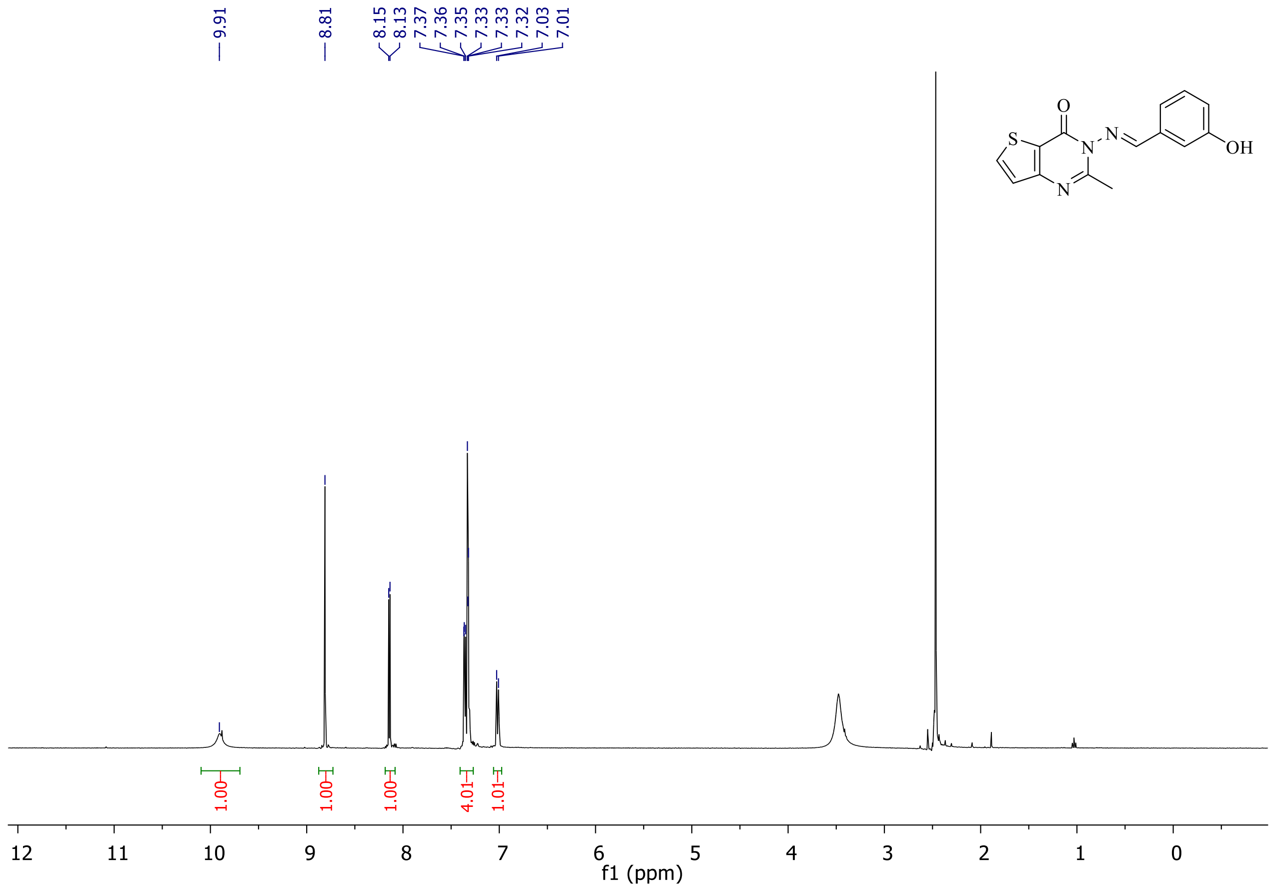

^{1}H NMR Spectrum of Compound 2

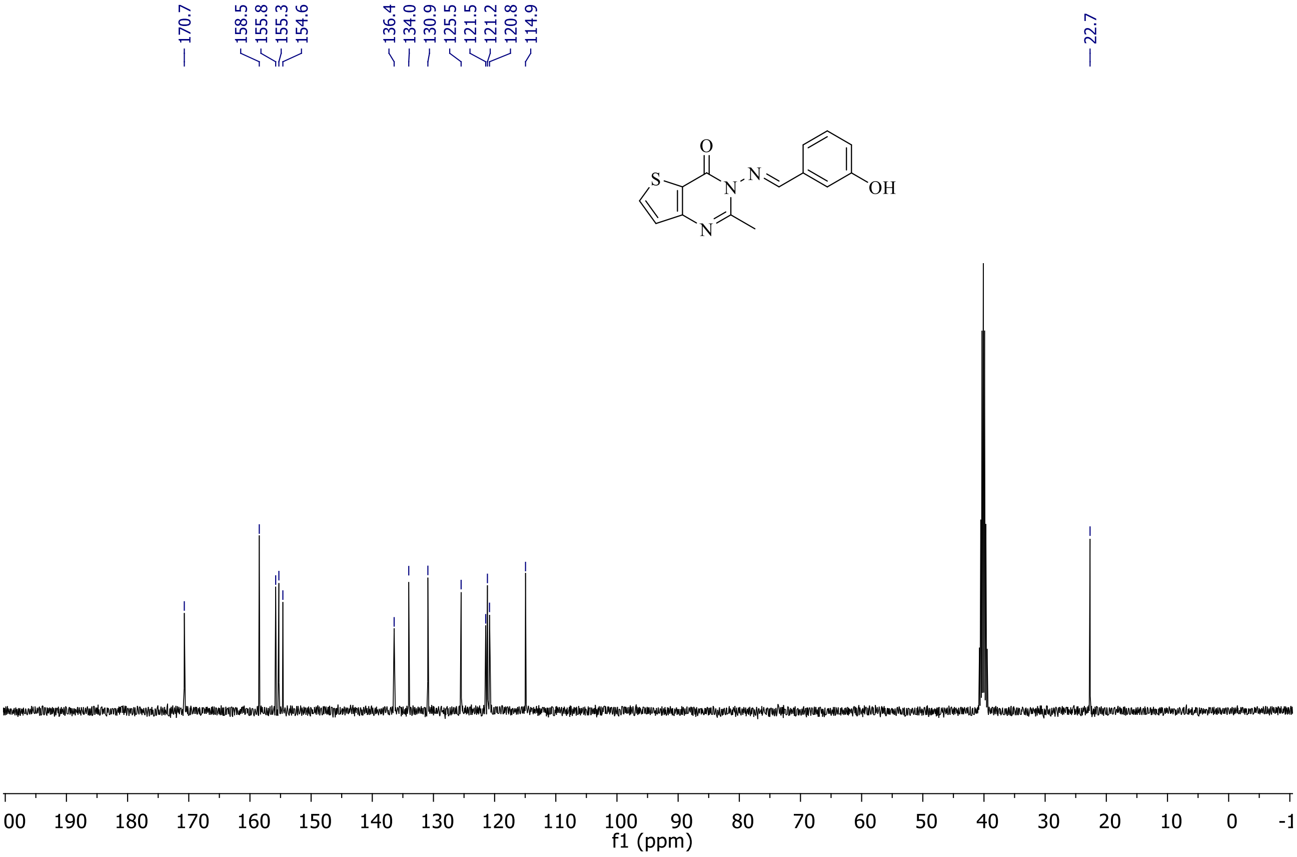

^{13}C NMR Spectrum of Compound 2

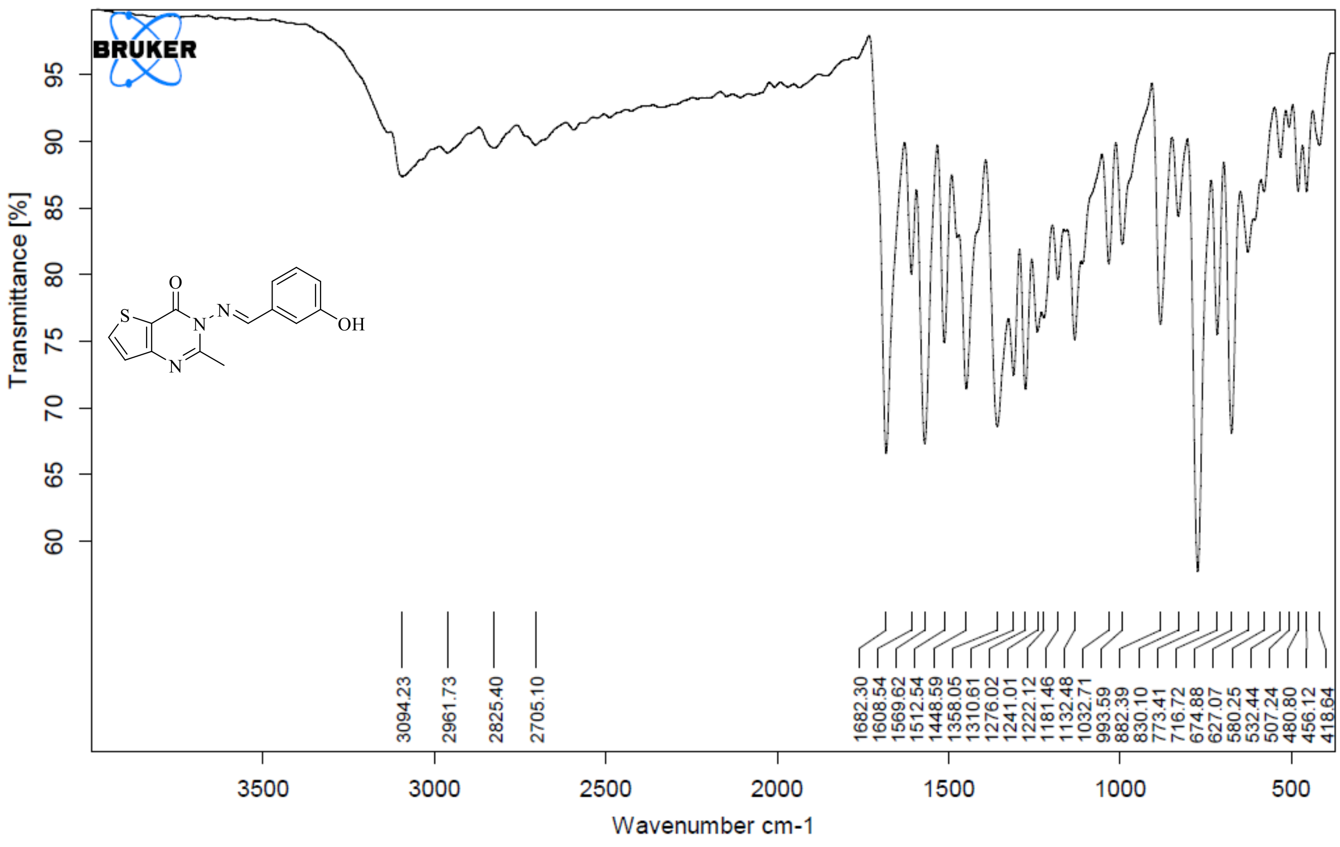

FTIR Spectrum of Compound 2

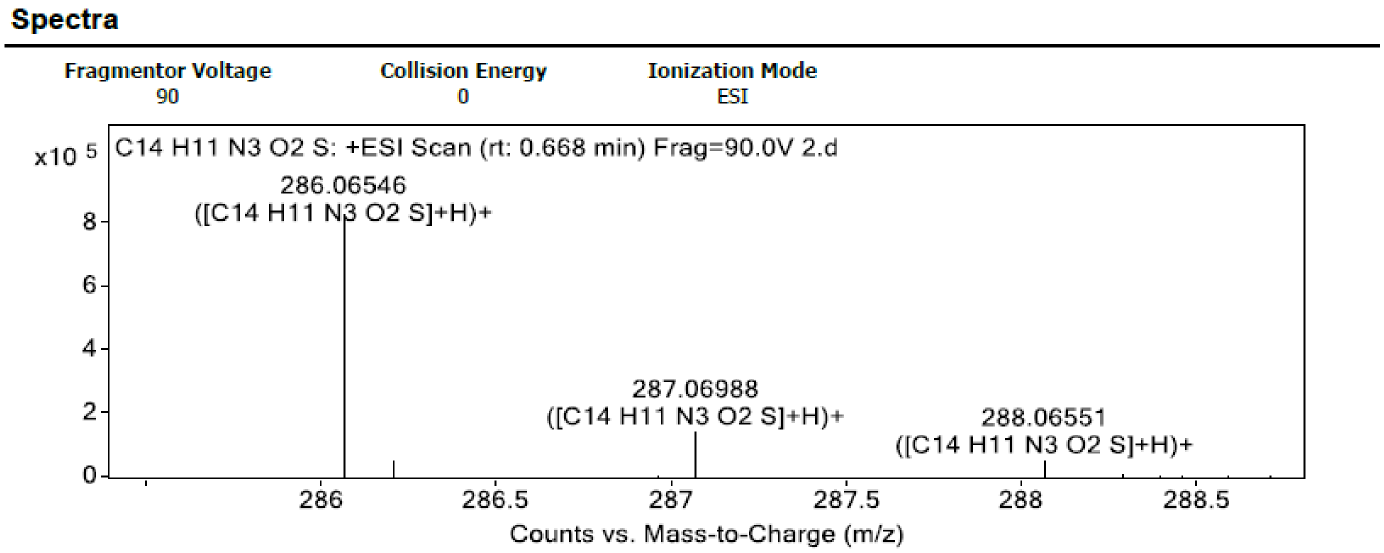

HRMS Spectrum of Compound 2

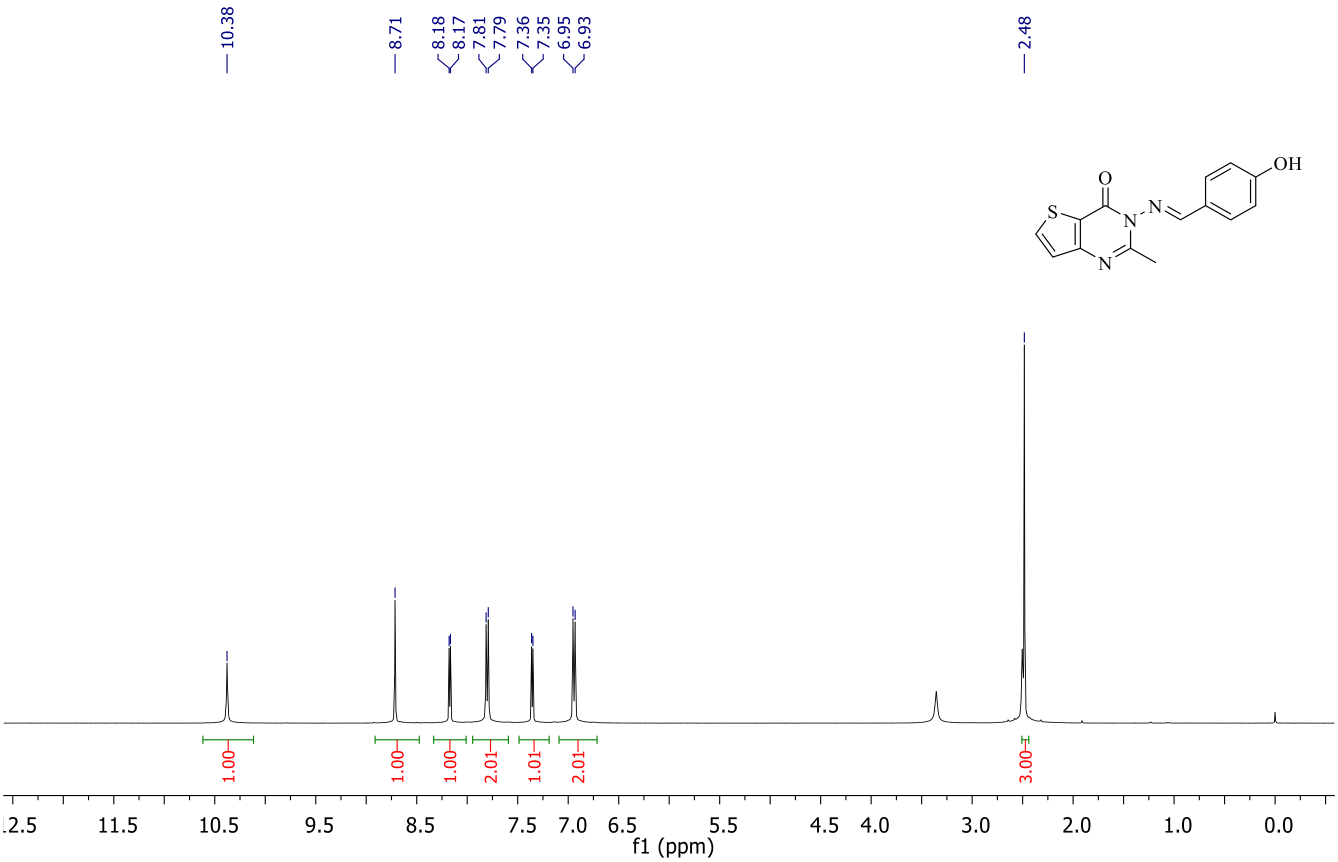

^{1}H NMR Spectrum of Compound 3

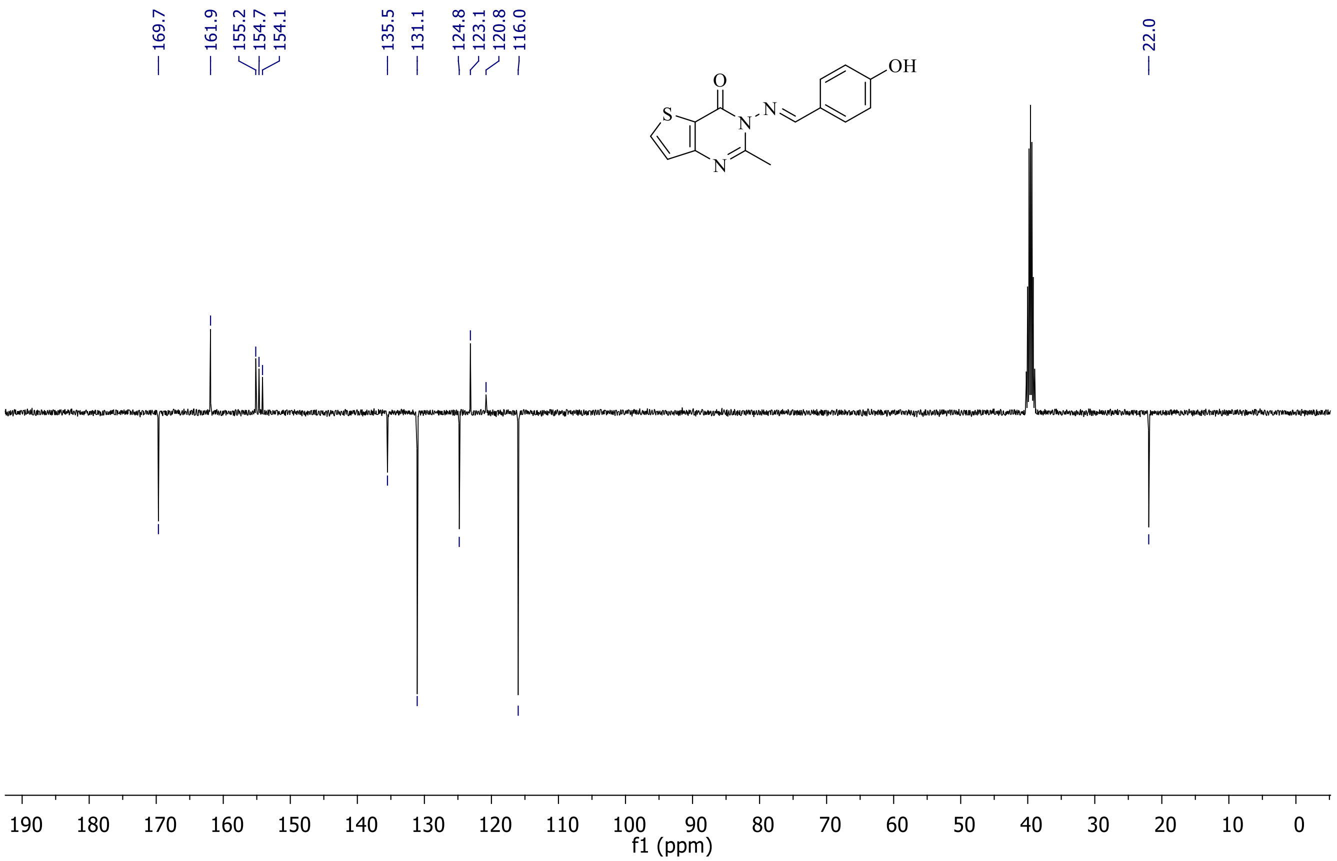

^{13}C NMR-APT Spectrum of Compound 3

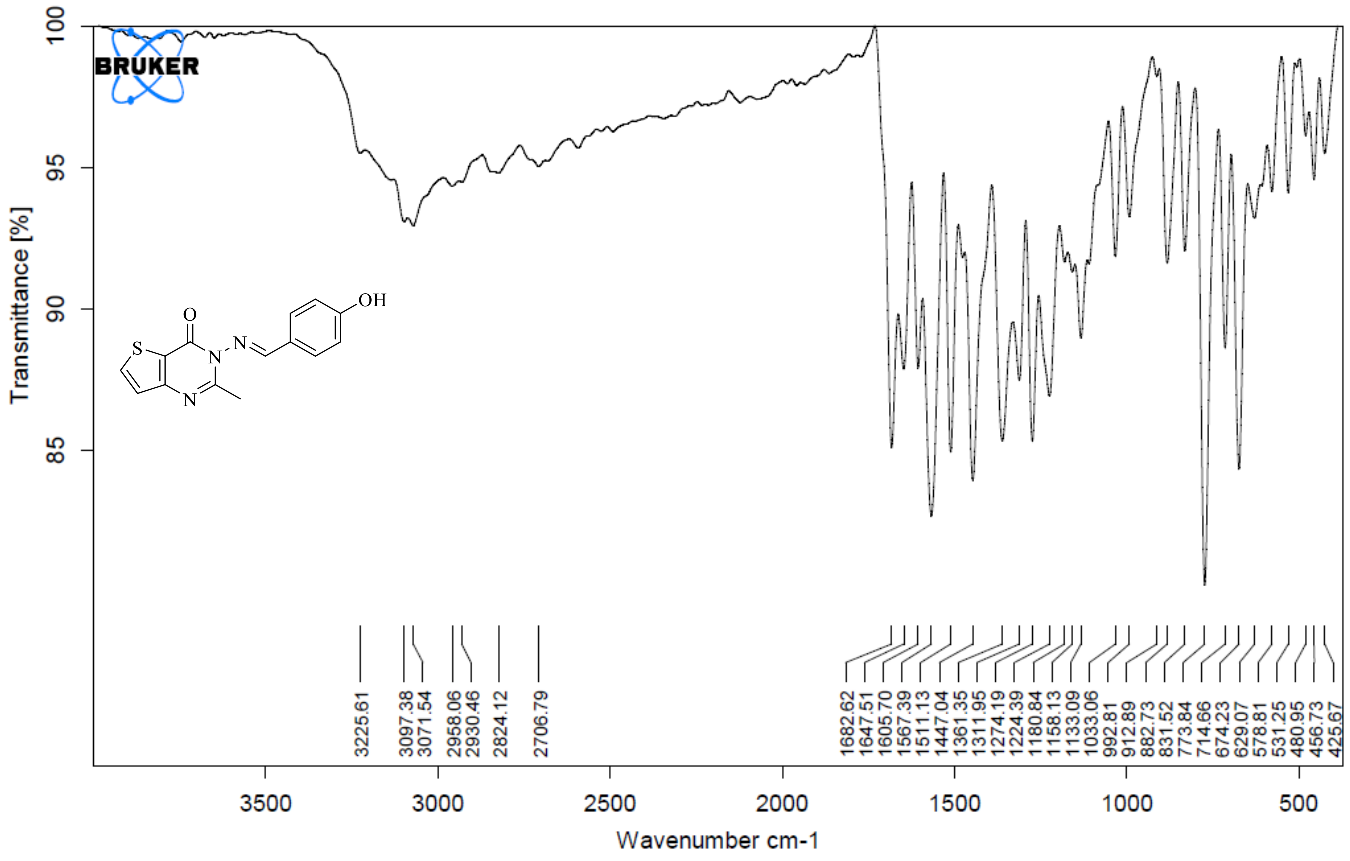

FTIR Spectrum of Compound 3

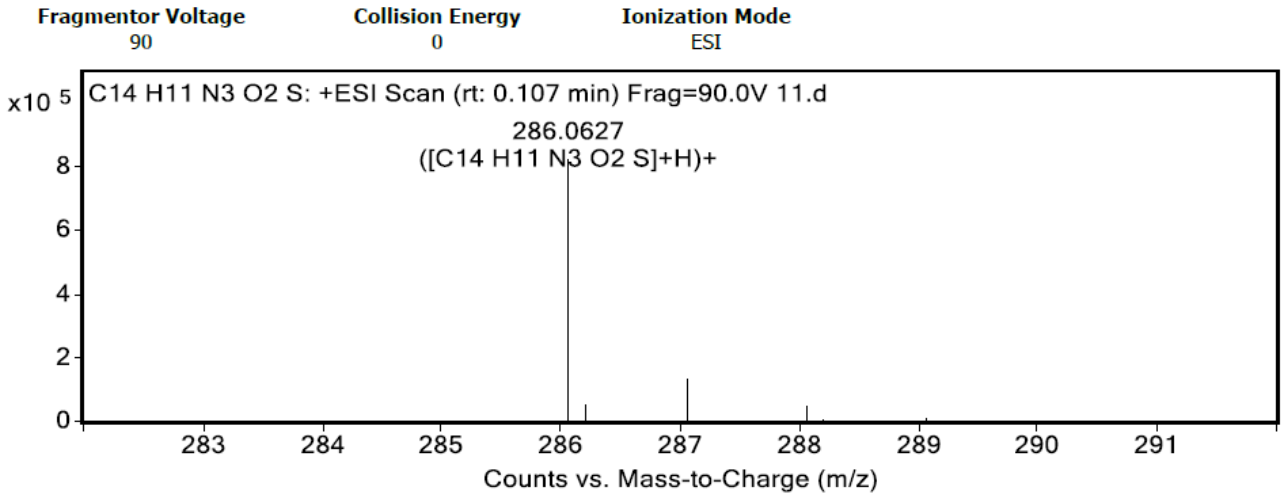

HRMS Spectrum of Compound 3

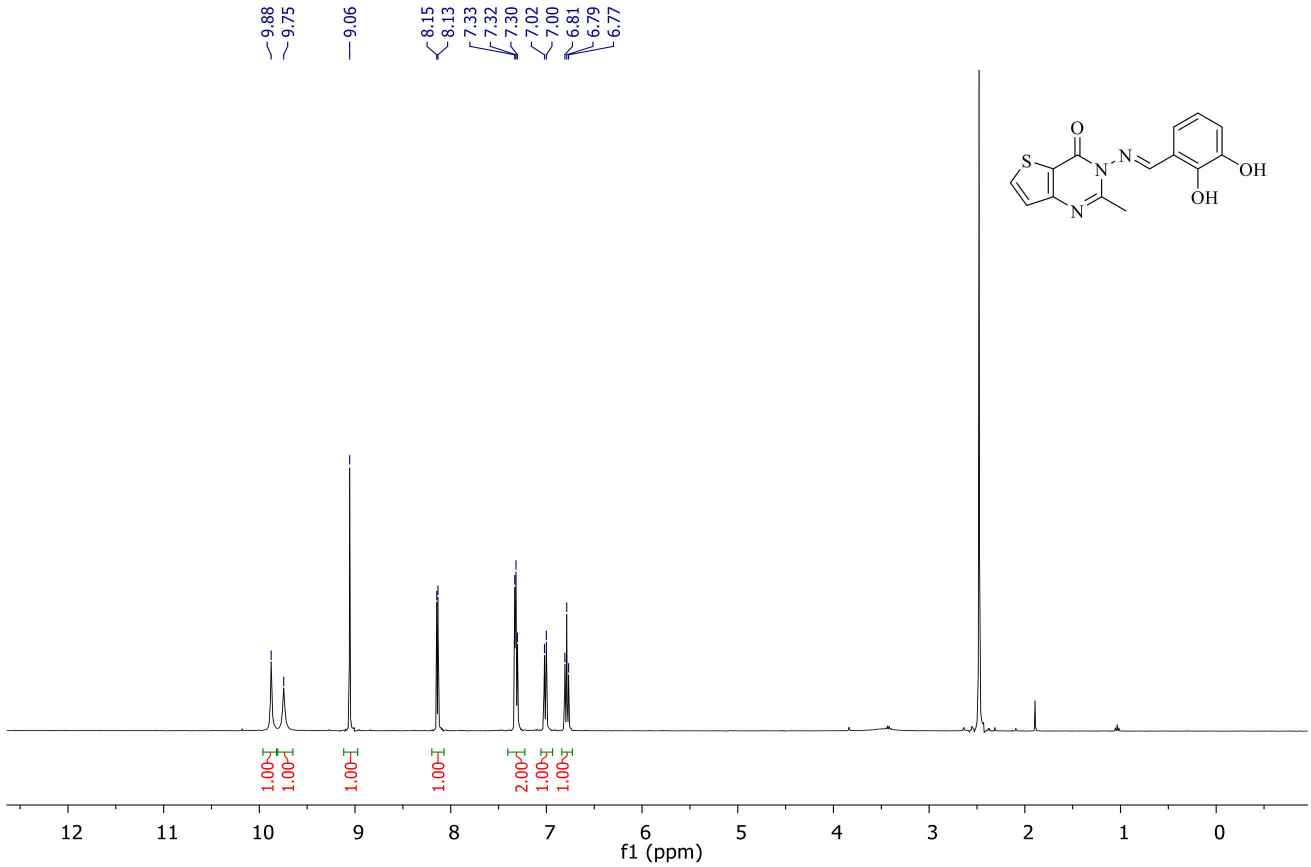

^{1}H NMR Spectrum of Compound 4

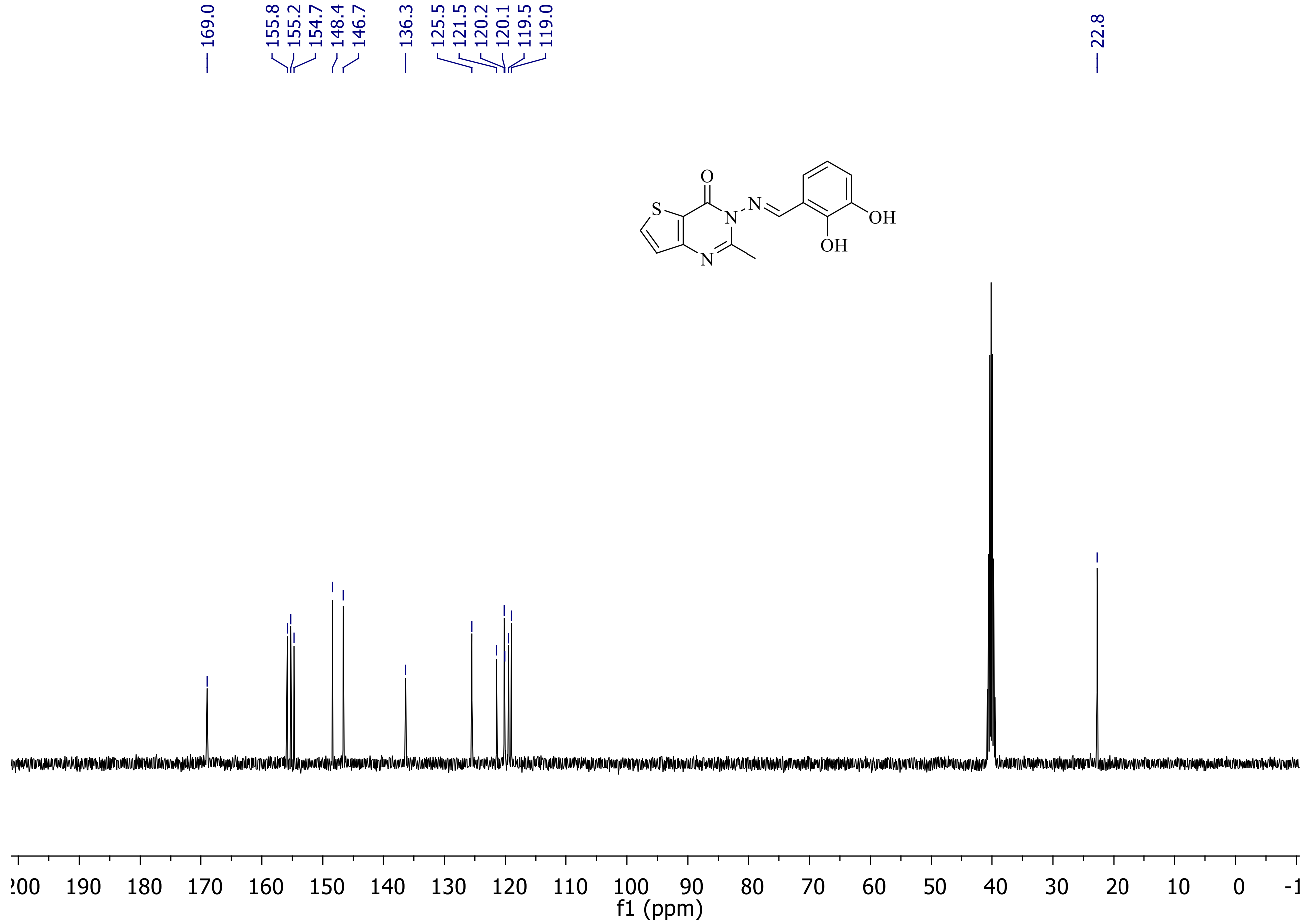

^{13}C NMR Spectrum of Compound 4

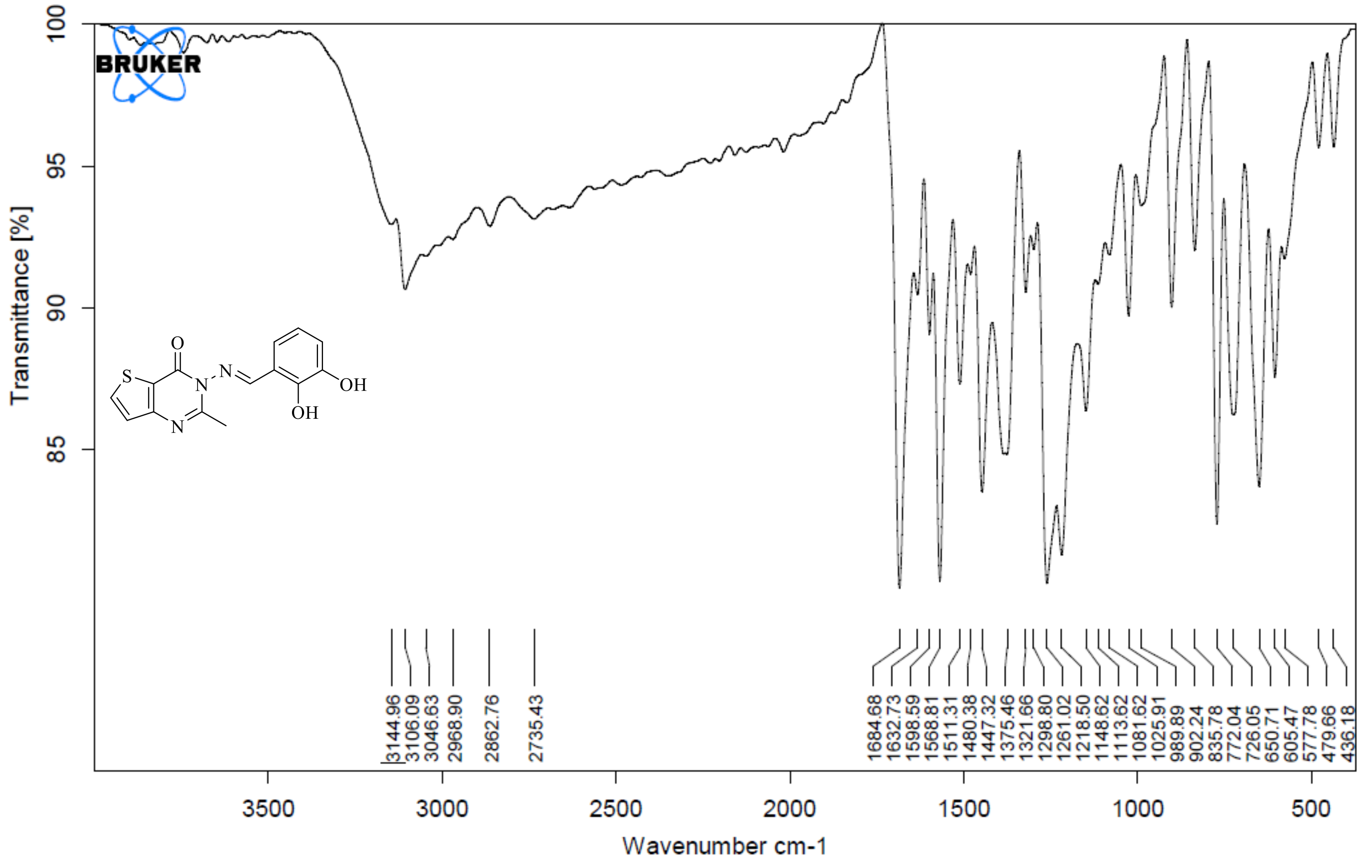

FTIR Spectrum of Compound 4

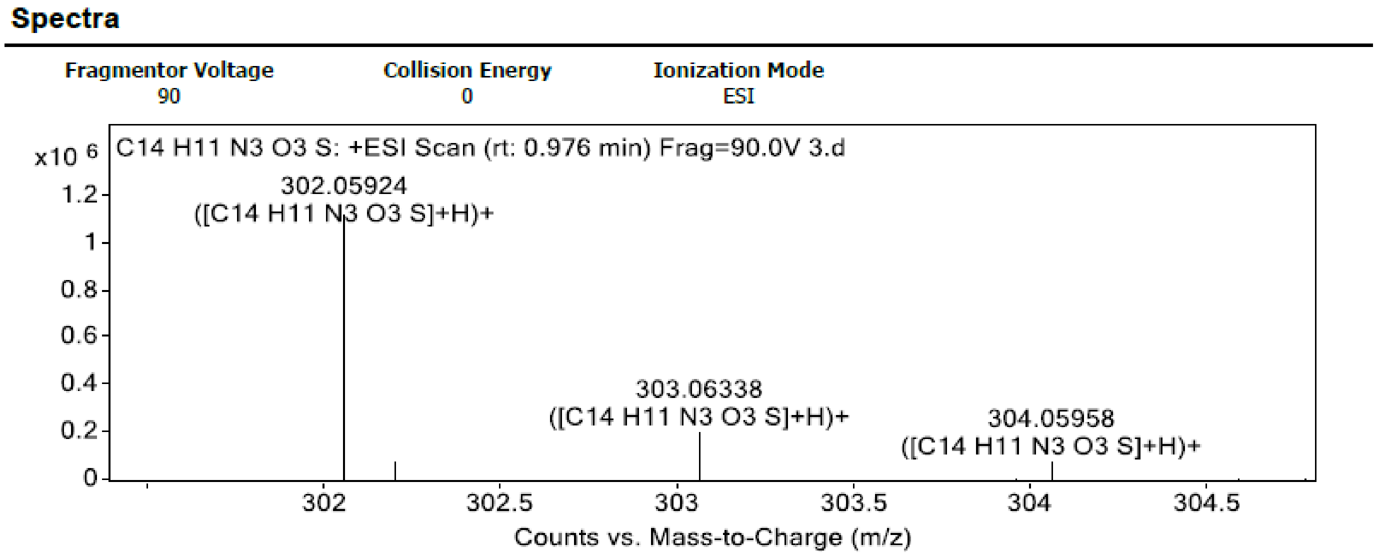

HRMS Spectrum of Compound 4

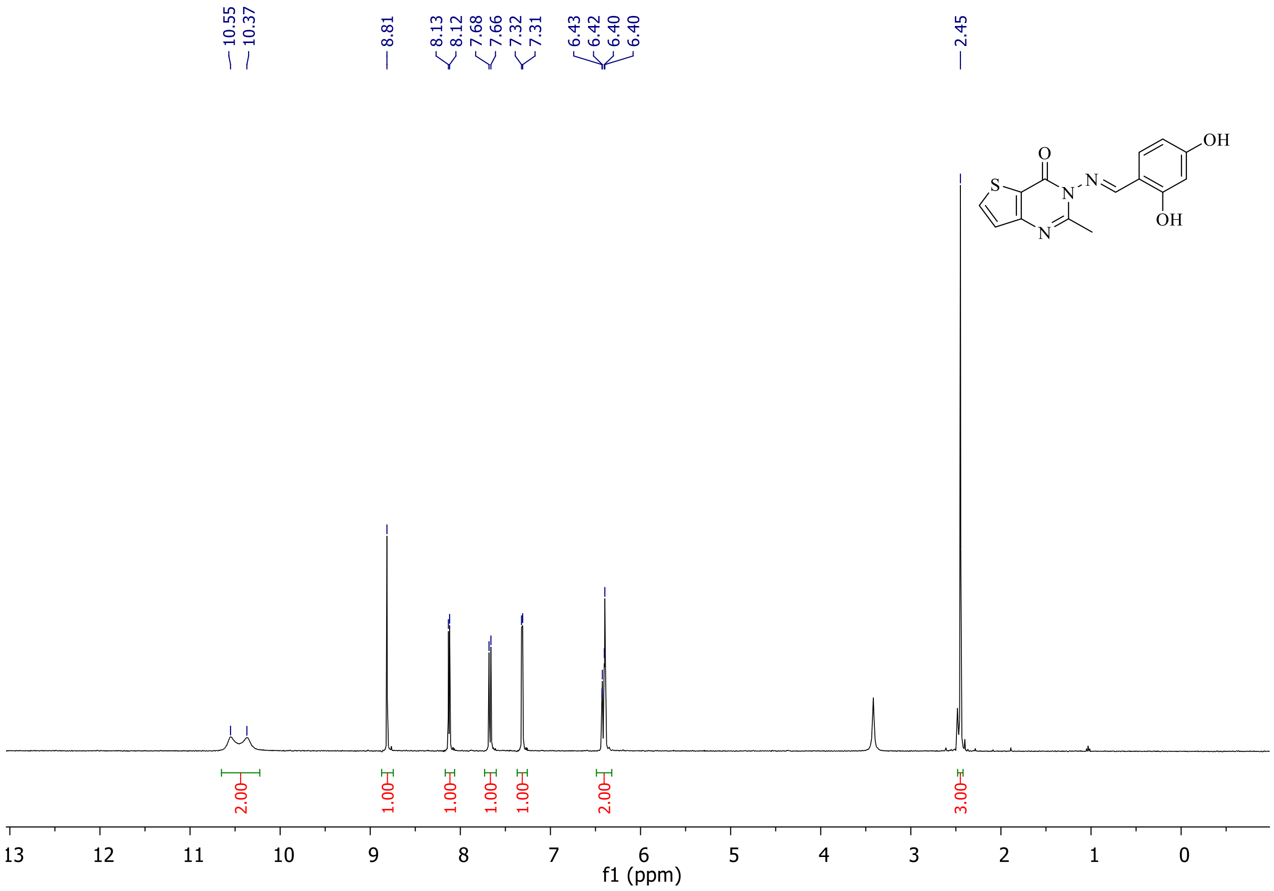

^{1}H NMR Spectrum of Compound 5

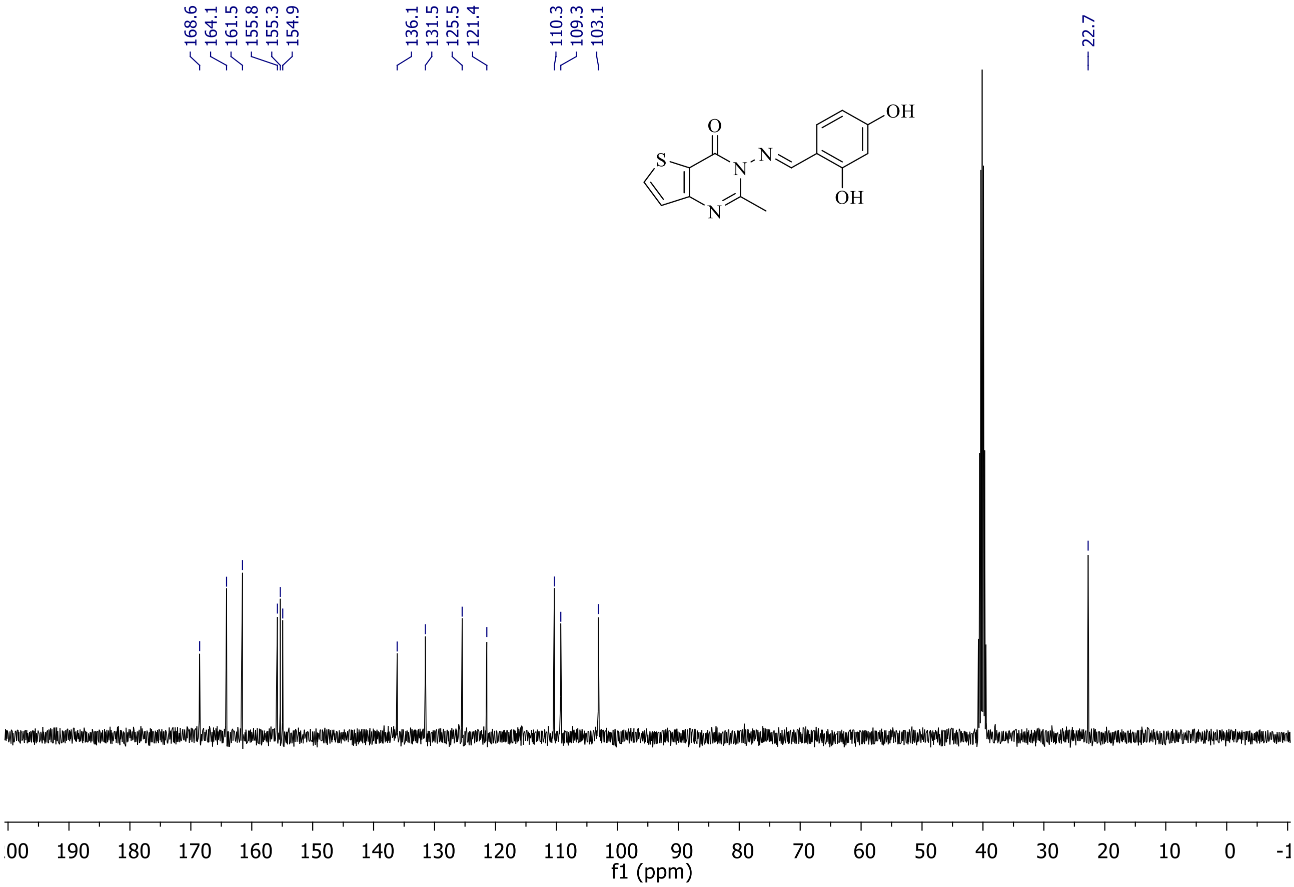

^{13}C NMR Spectrum of Compound 5

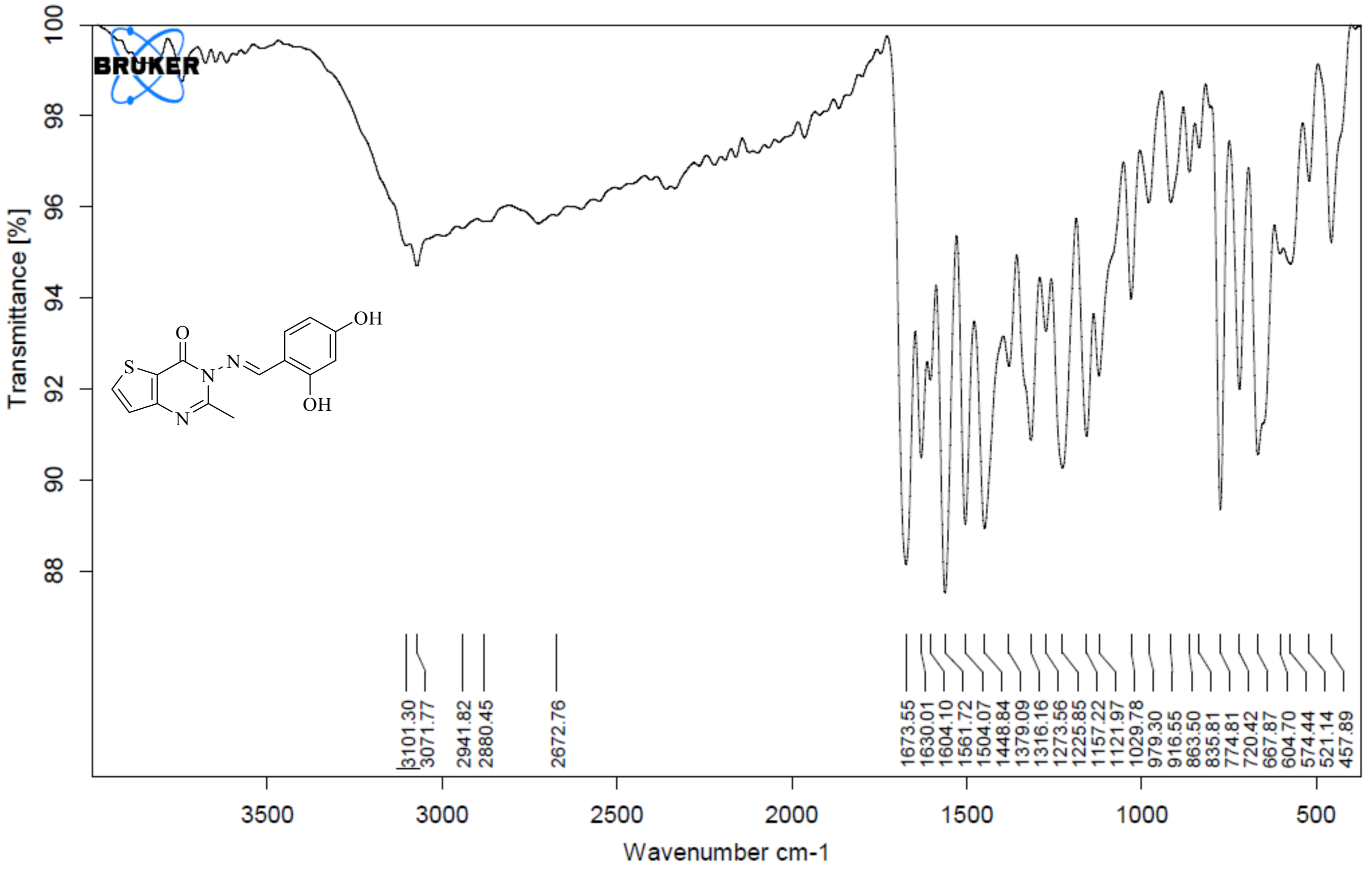

FTIR Spectrum of Compound 5

Spectra

Fragmentor Voltage	Collision Energy	Ionization Mode
90	0	ESI

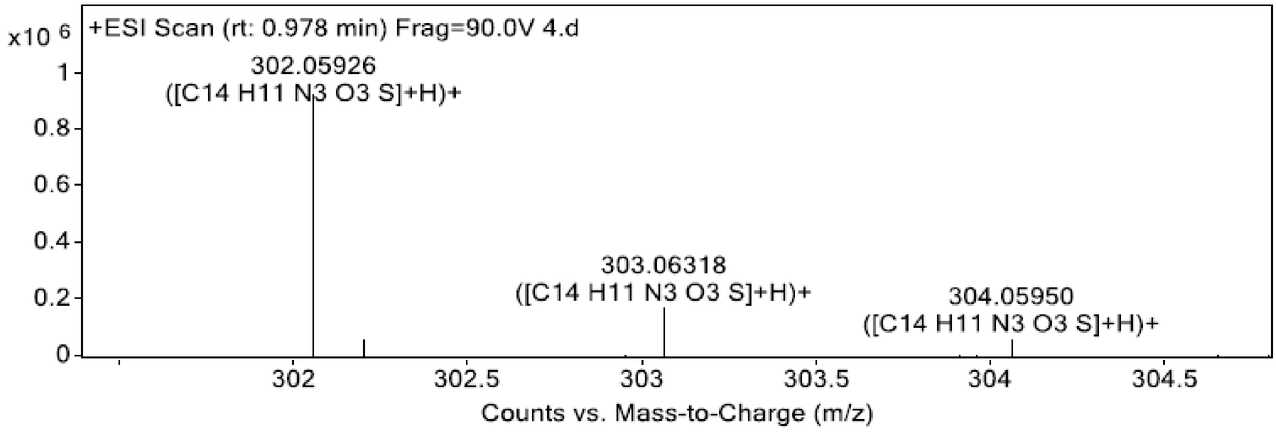

HRMS Spectrum of Compound 5

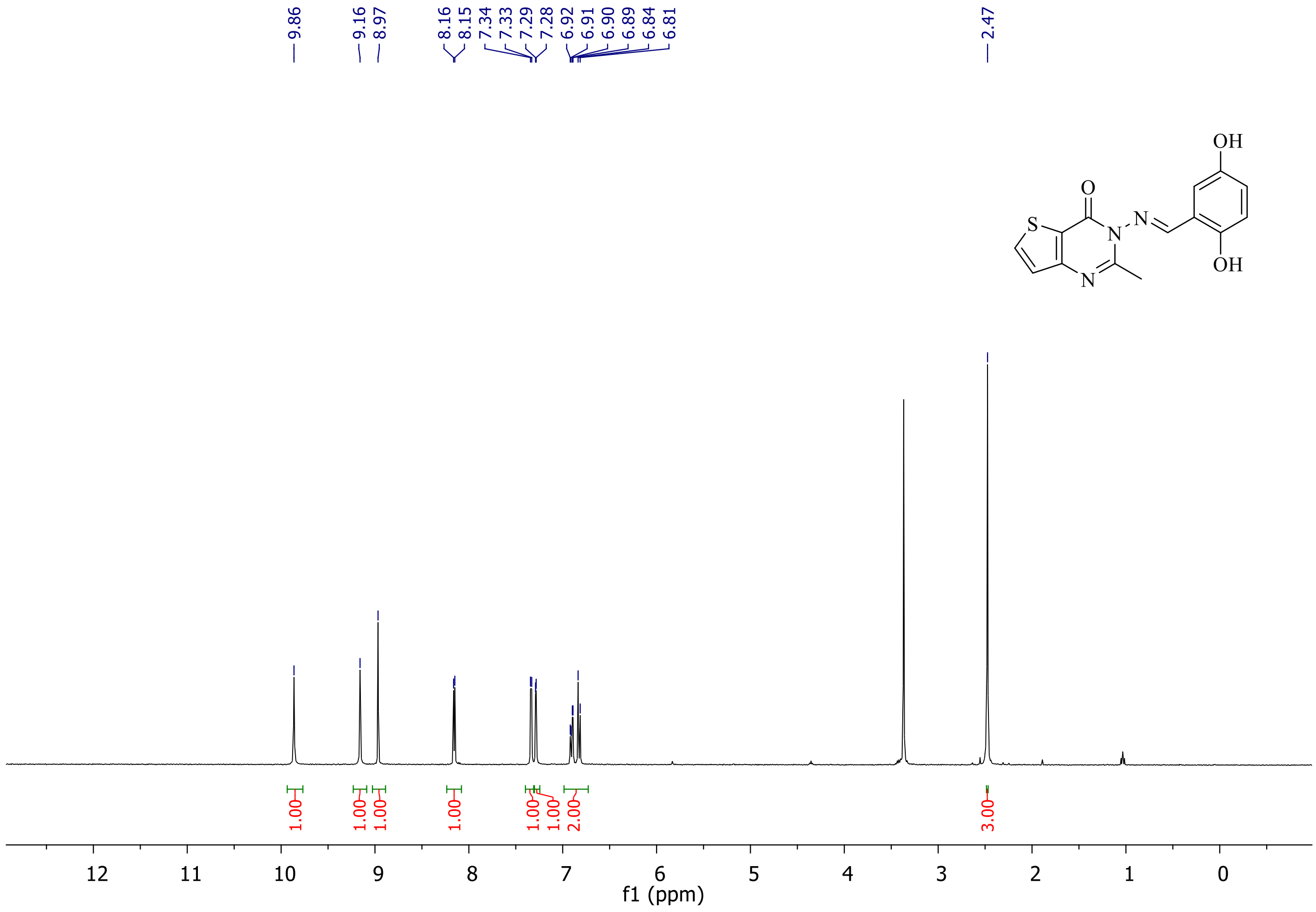

^{1}H NMR Spectrum of Compound 6

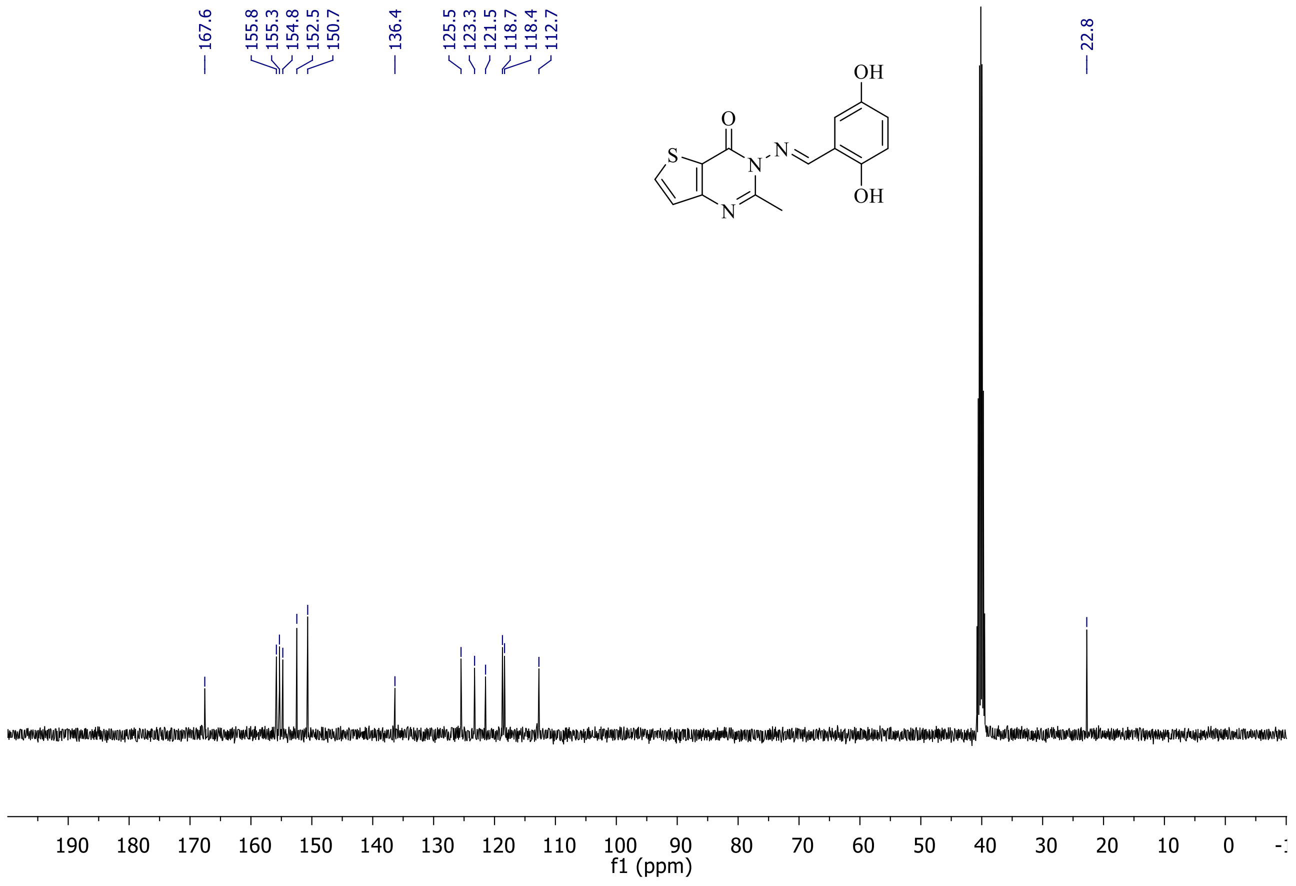

^{13}C NMR Spectrum of Compound 6

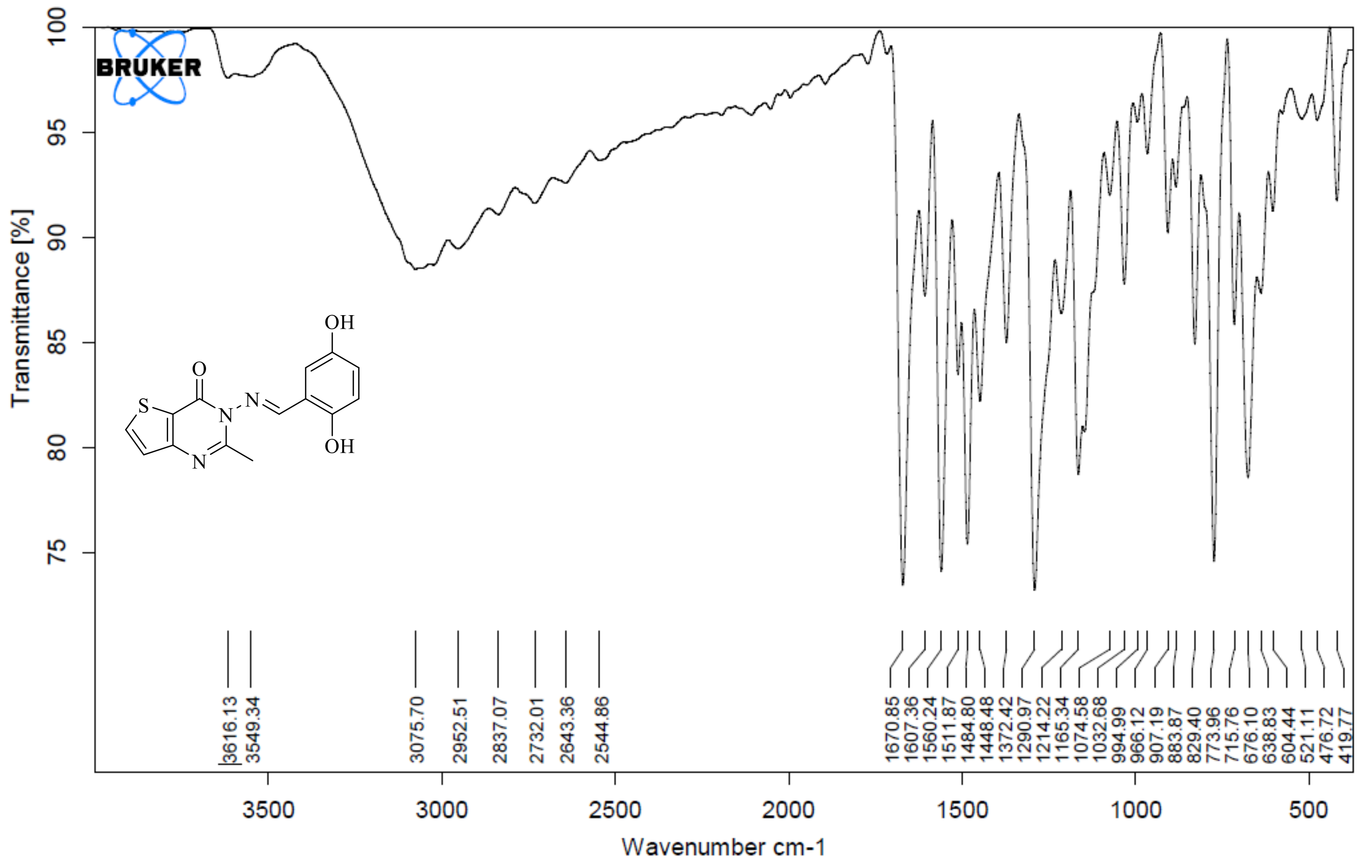

FTIR Spectrum of Compound 6

Spectra

Fragmentor Voltage	Collision Energy	Ionization Mode
90	0	ESI

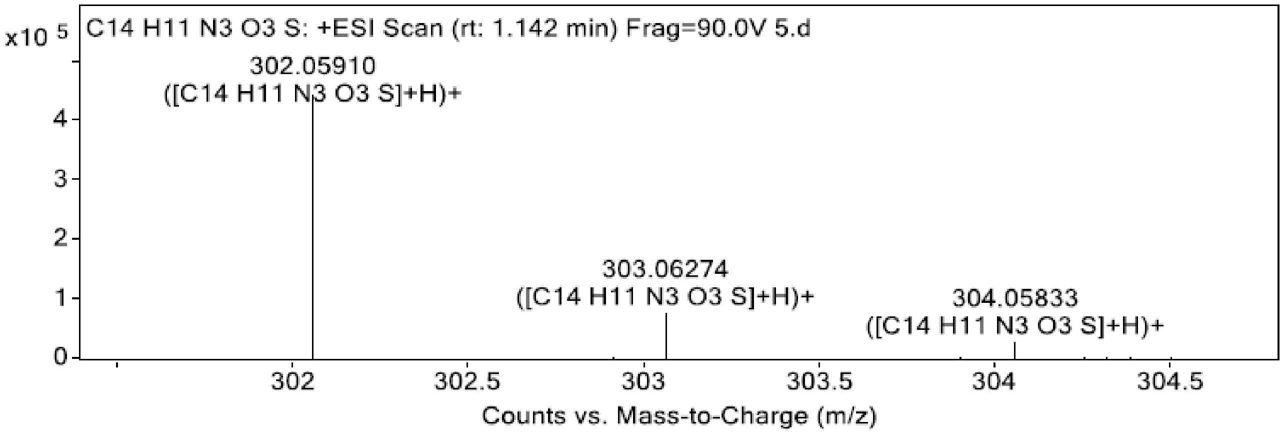

HRMS Spectrum of Compound 6

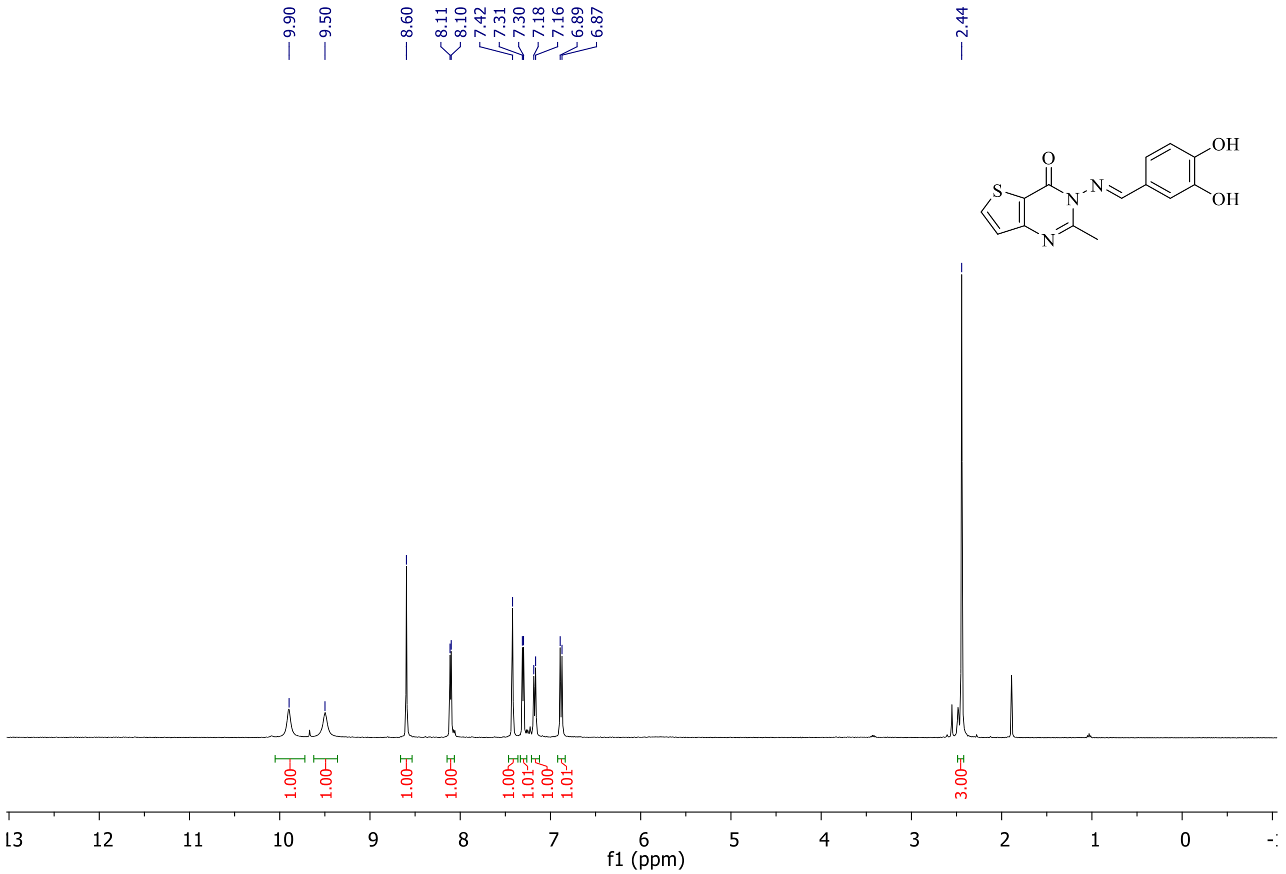

^{1}H NMR Spectrum of Compound 7

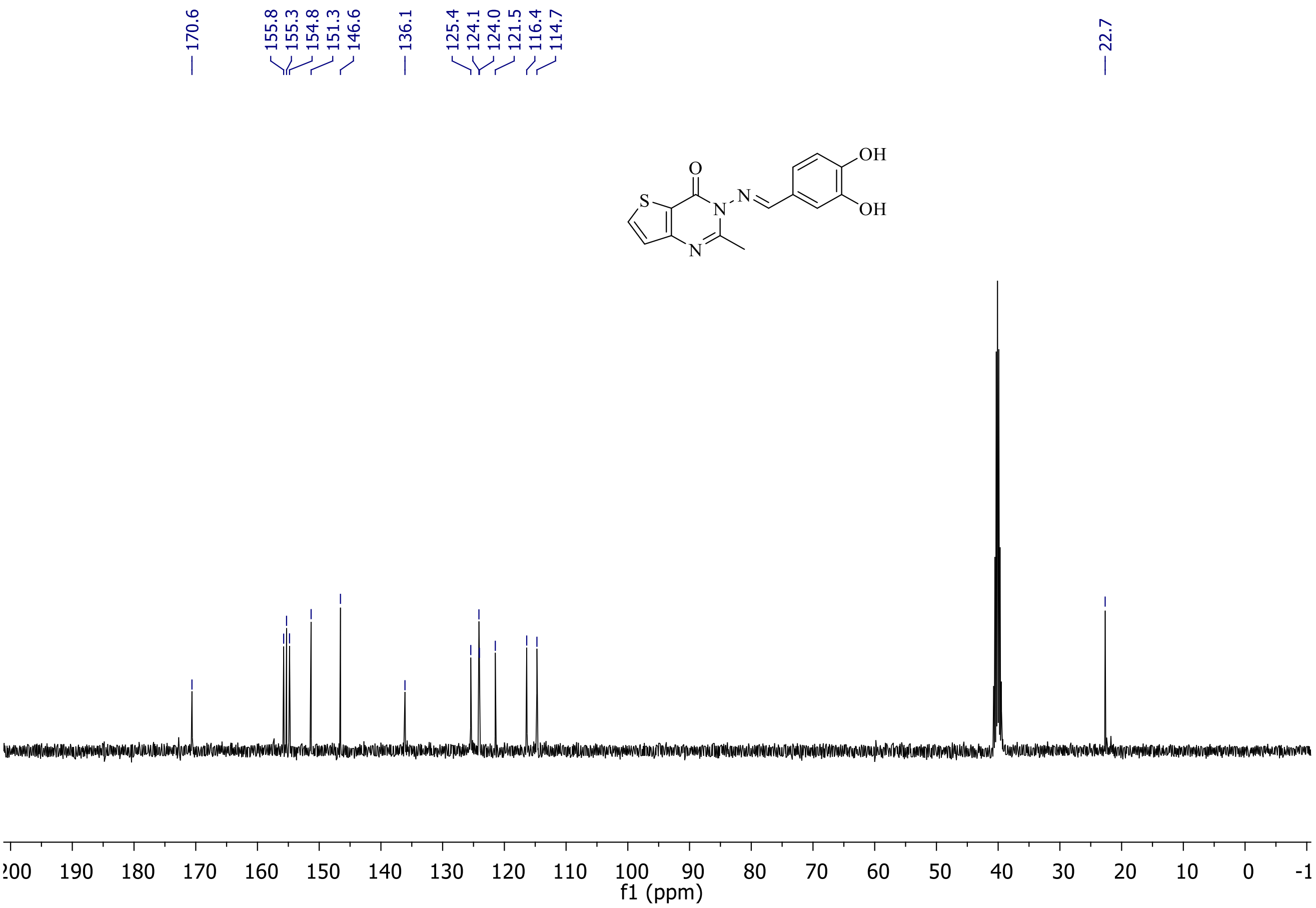

^{13}C NMR Spectrum of Compound 7

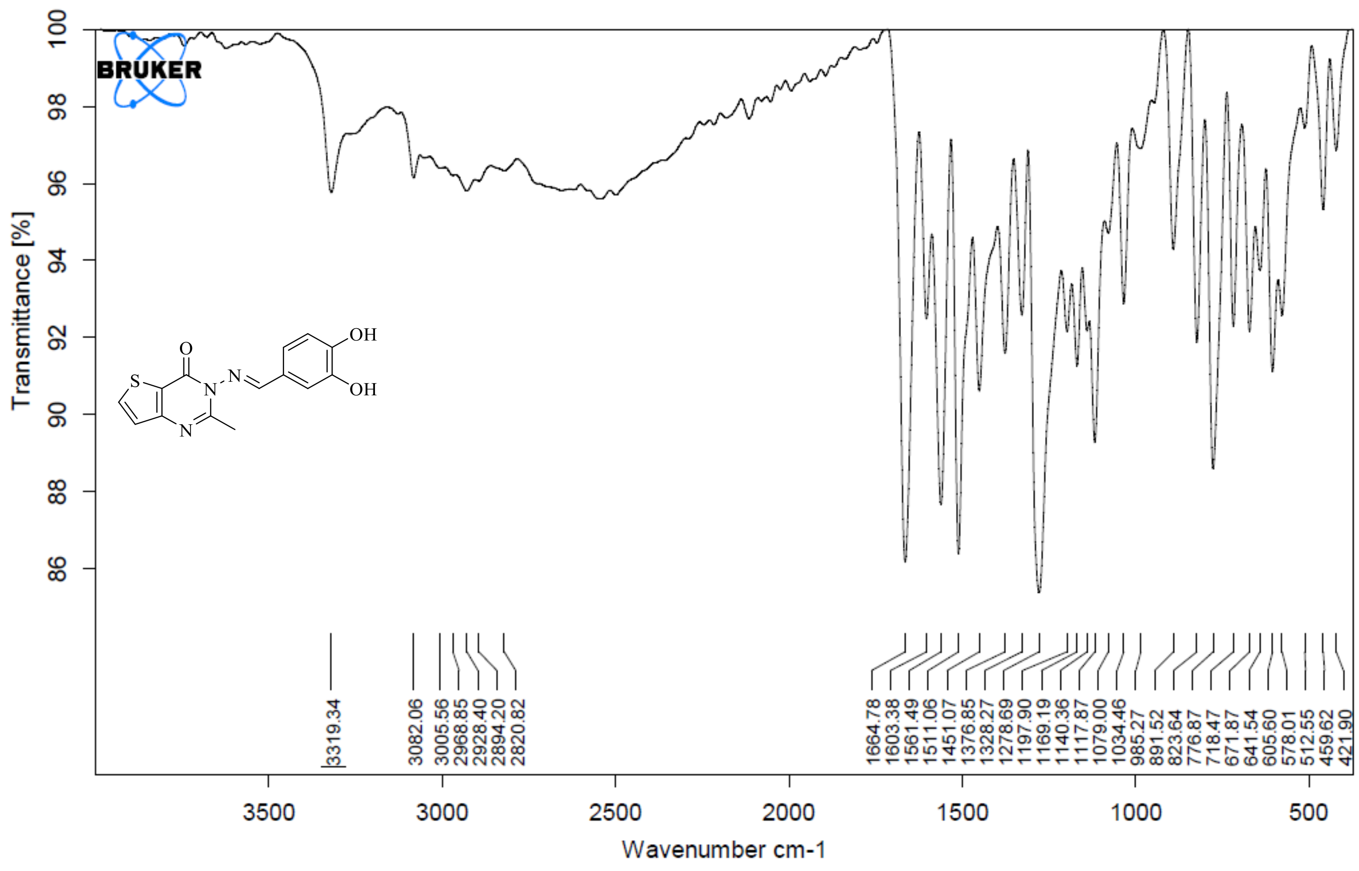

FTIR Spectrum of Compound 7

Spectra

Fragmentor Voltage	Collision Energy	Ionization Mode
90	0	ESI

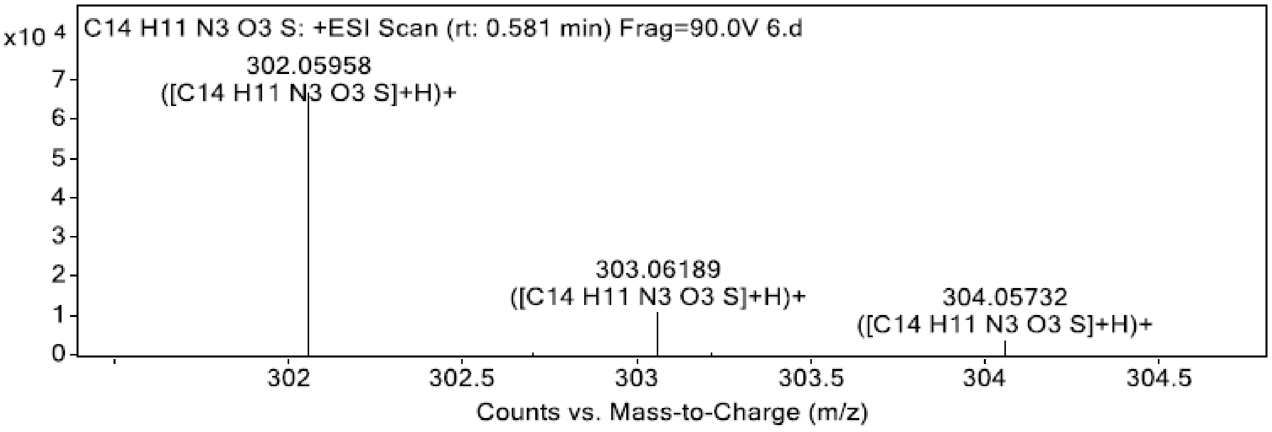

HRMS Spectrum of Compound 7

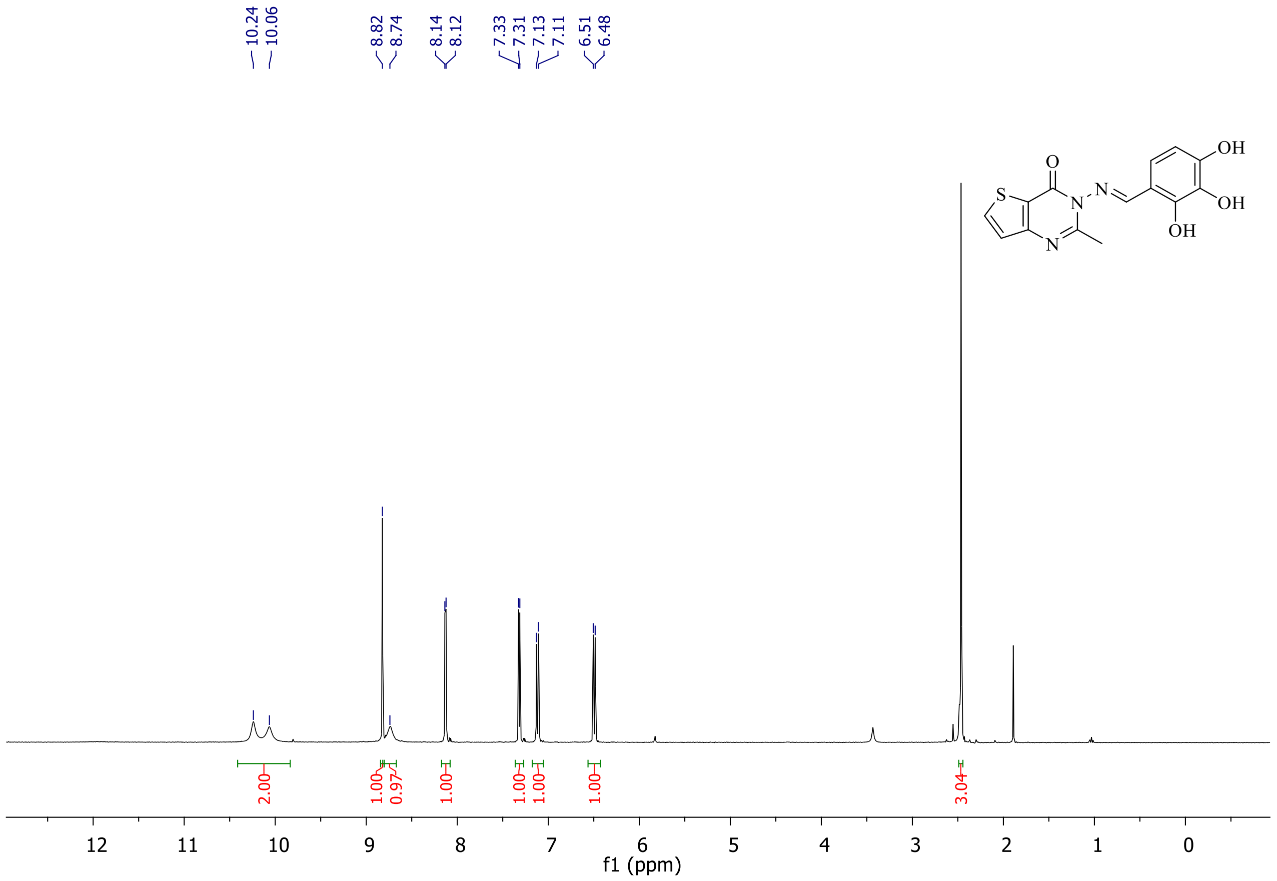

^{1}H NMR Spectrum of Compound 8

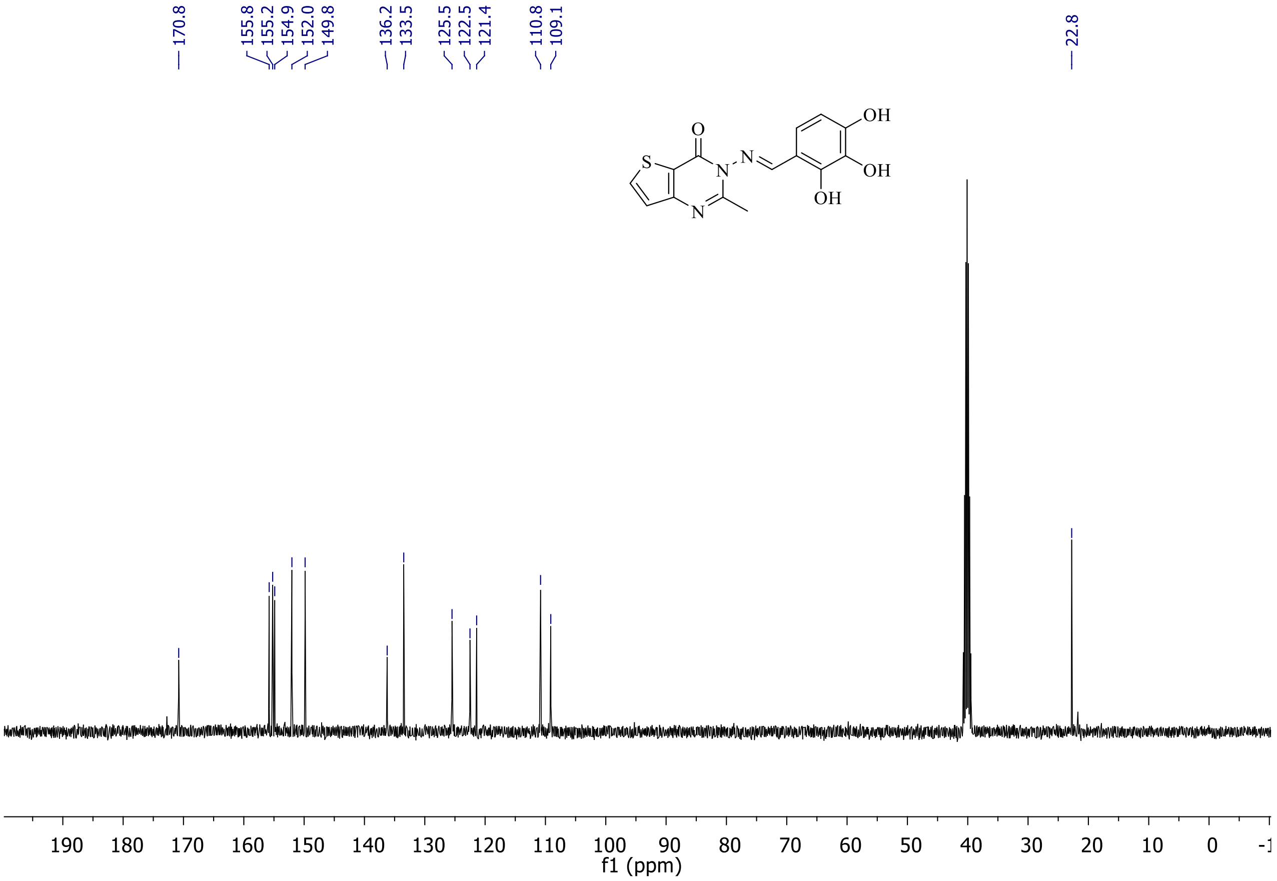

^{13}C NMR Spectrum of Compound 8

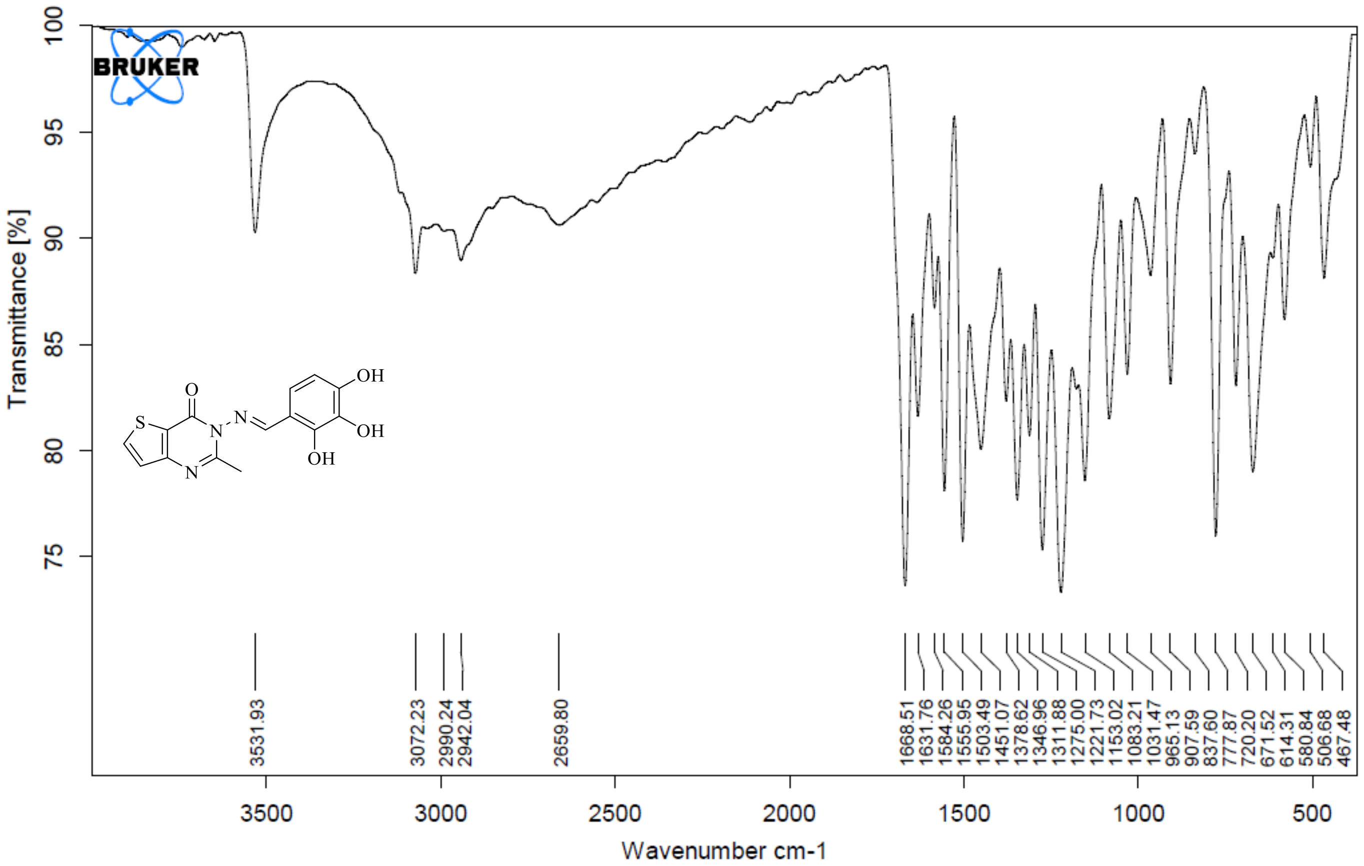

FTIR Spectrum of Compound 8

Spectra

Fragmentor Voltage	Collision Energy	Ionization Mode
90	0	ESI

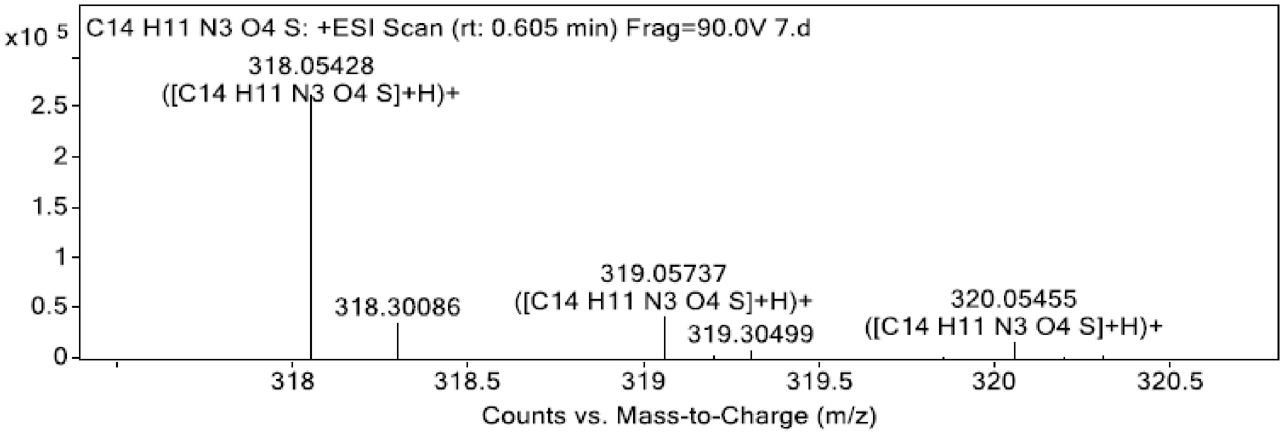

HRMS Spectrum of Compound 8

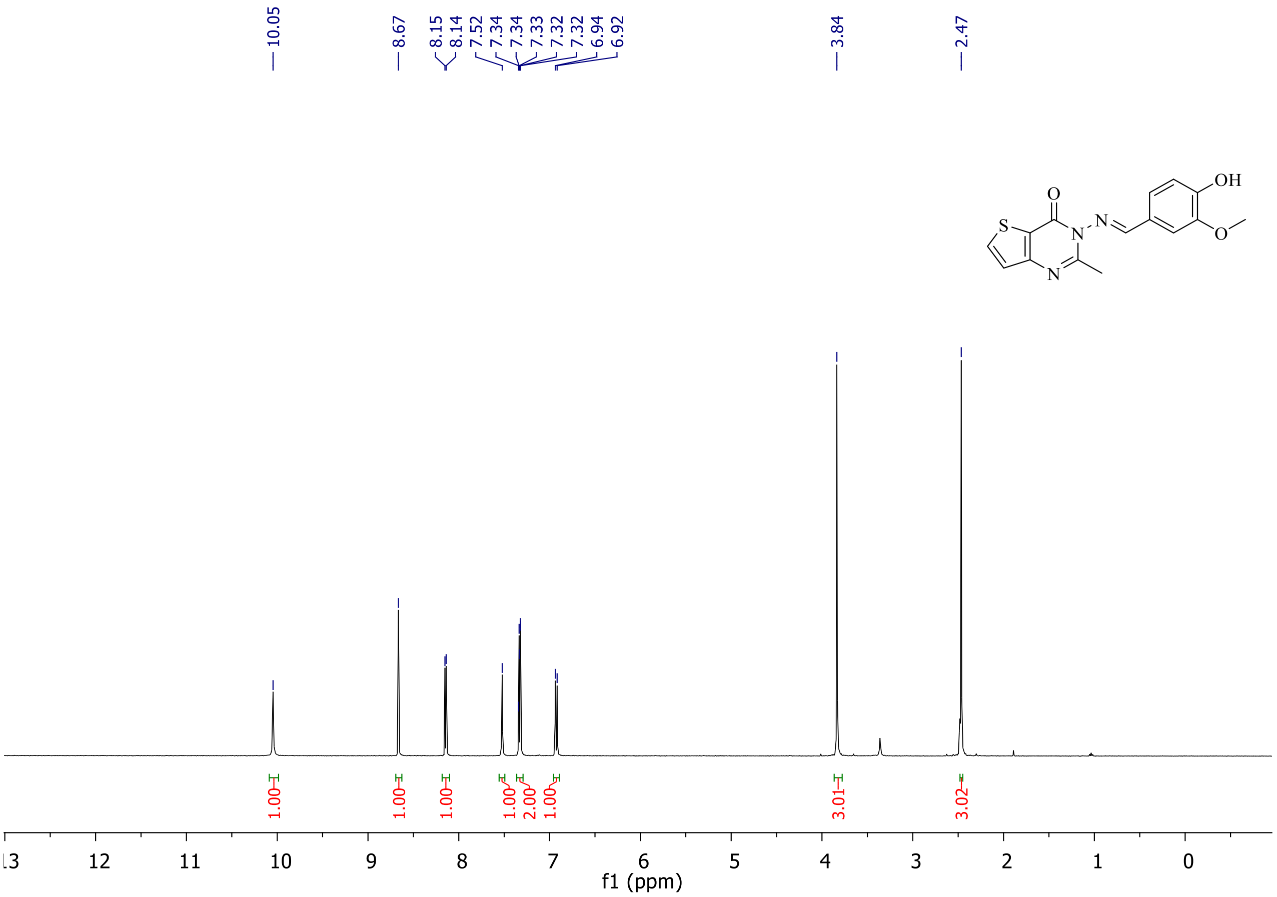

^{1}H NMR Spectrum of Compound 9

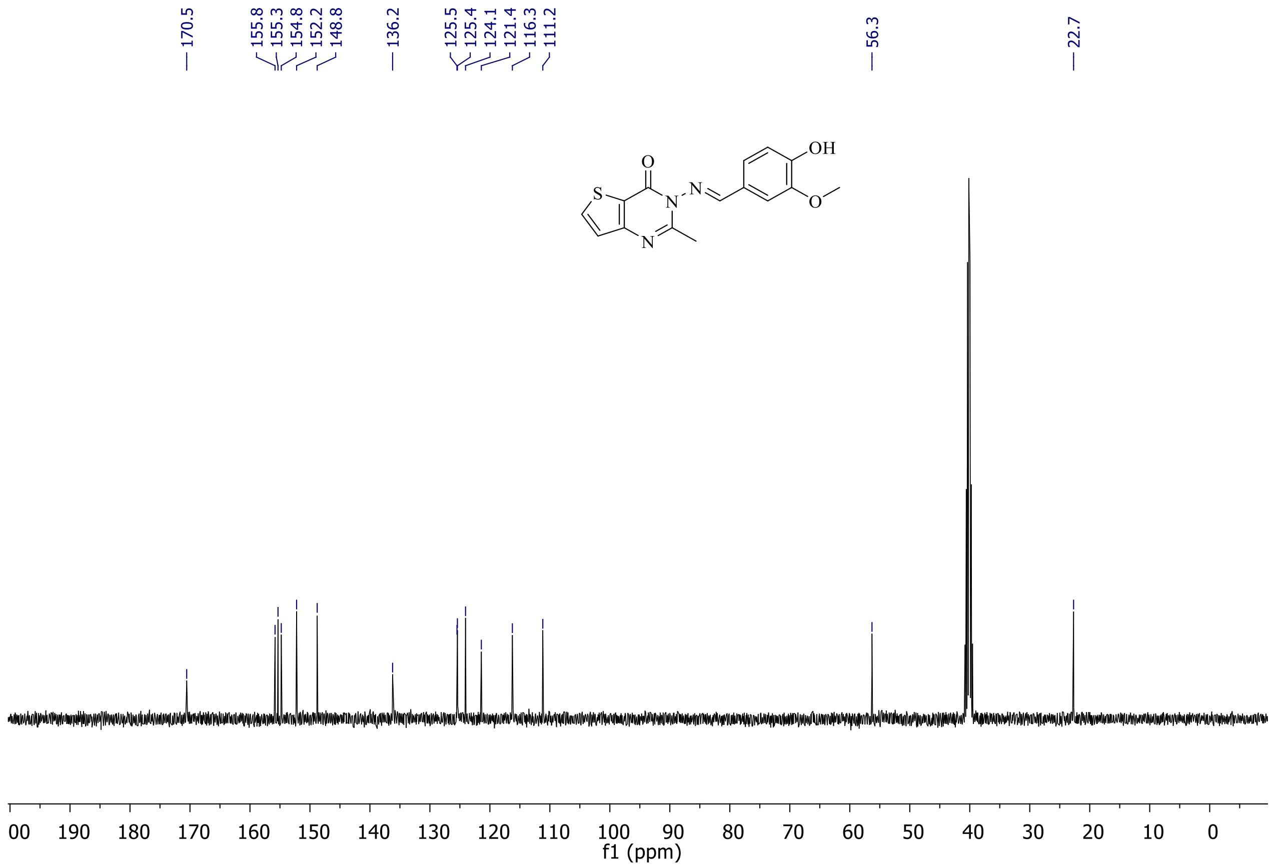

^{13}C NMR Spectrum of Compound 9

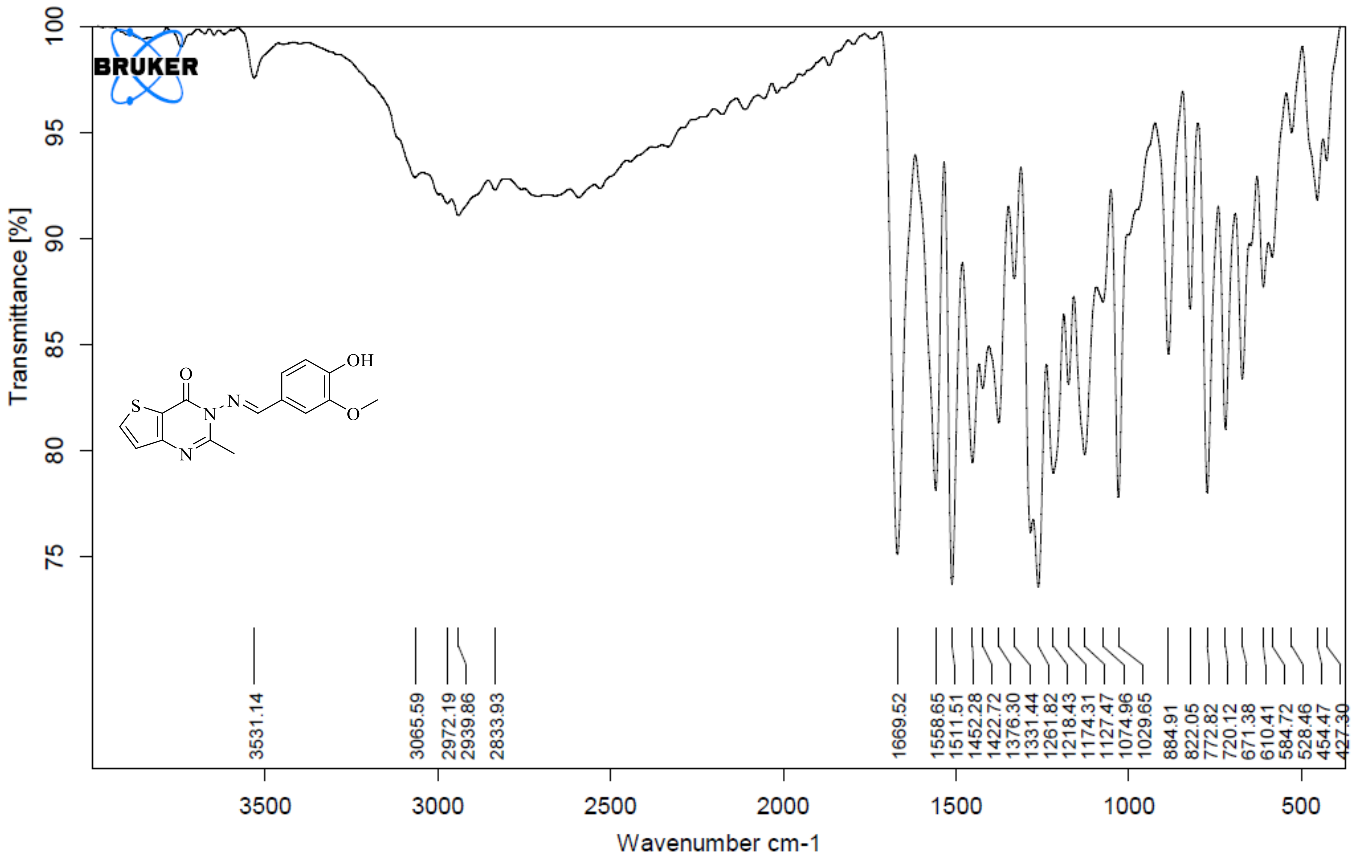

FTIR Spectrum of Compound 9

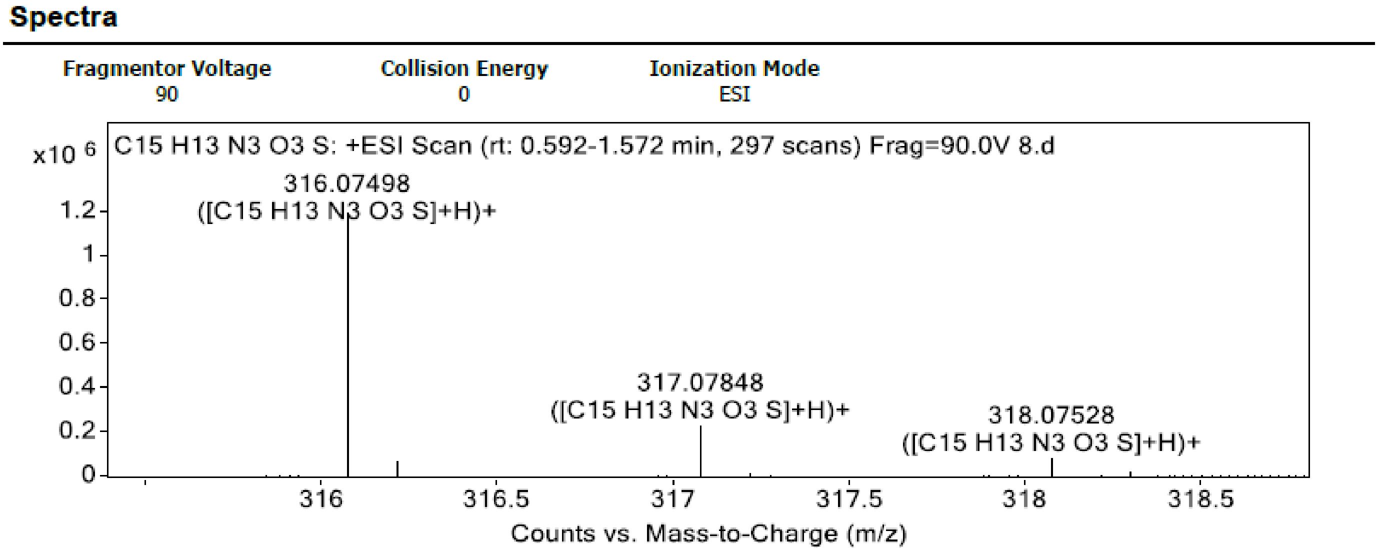

HRMS Spectrum of Compound 9

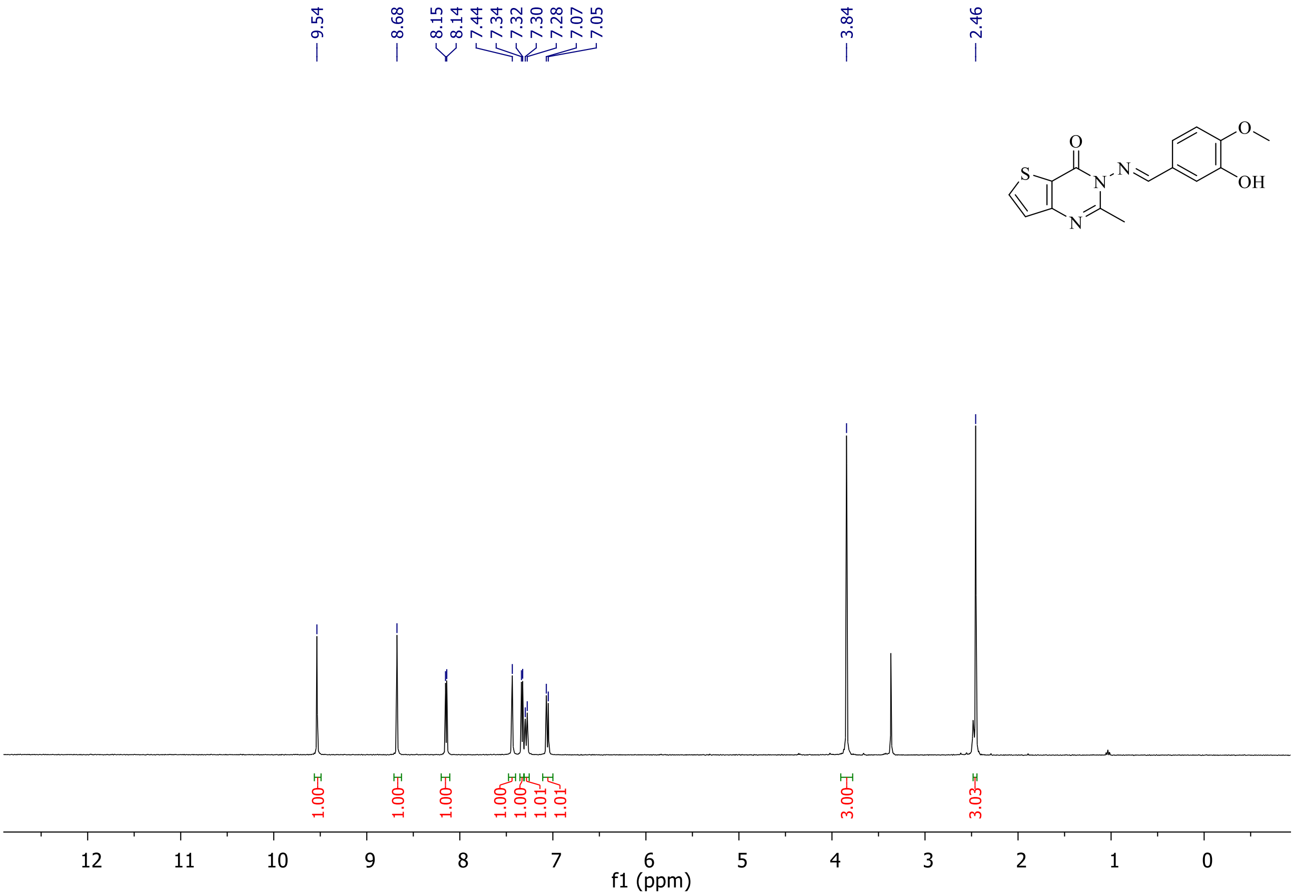

^{1}H NMR Spectrum of Compound 10

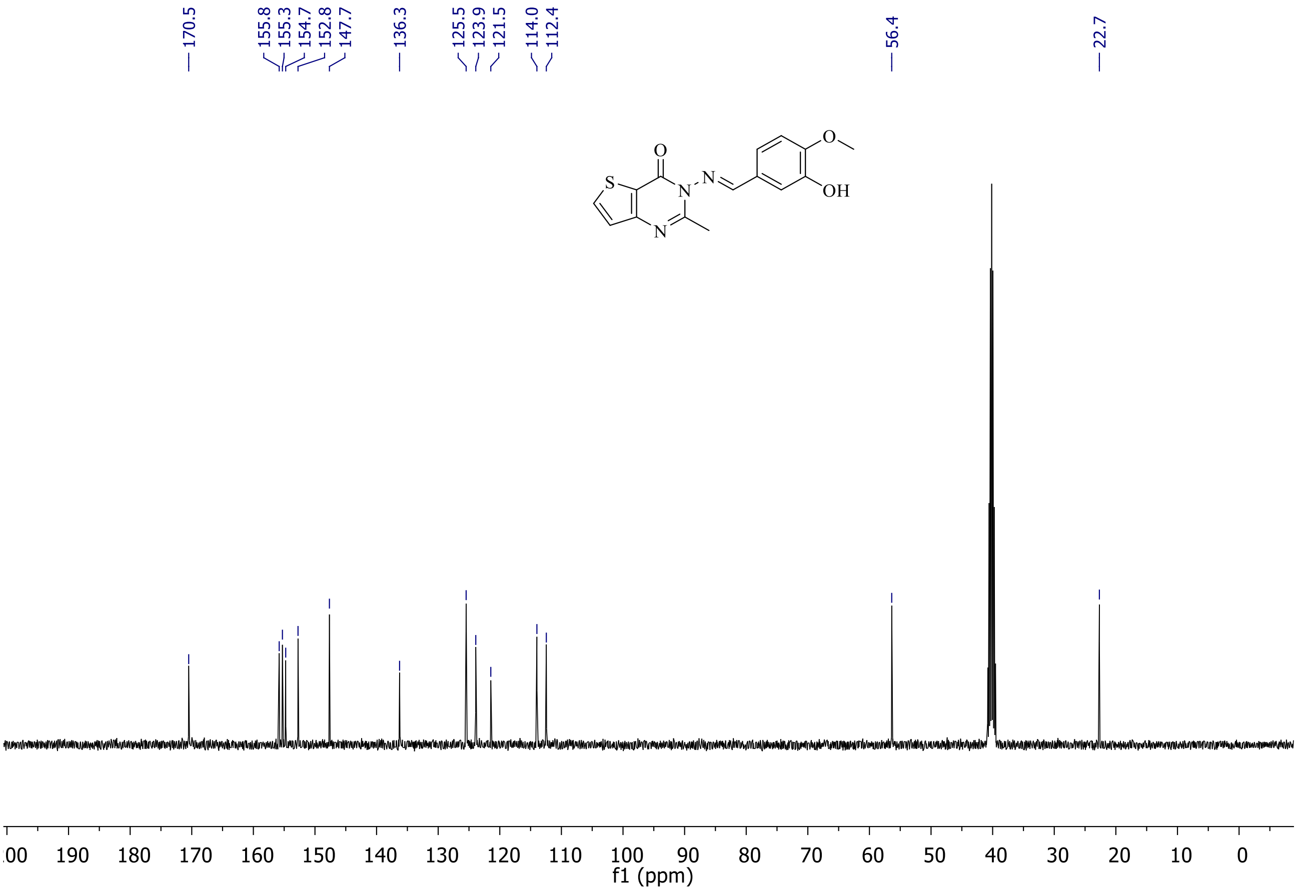

^{13}C NMR Spectrum of Compound 10

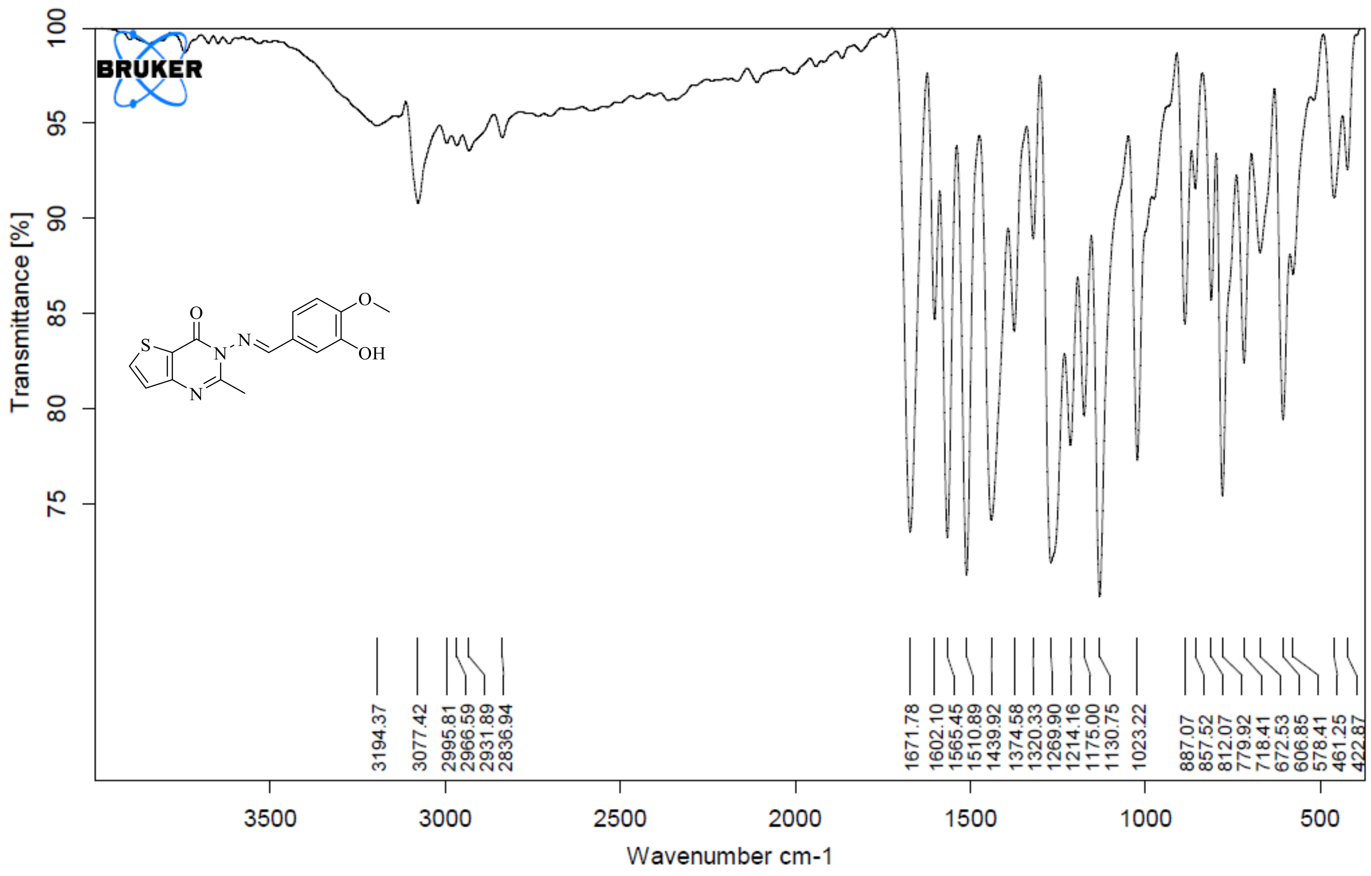

FTIR Spectrum of Compound 10

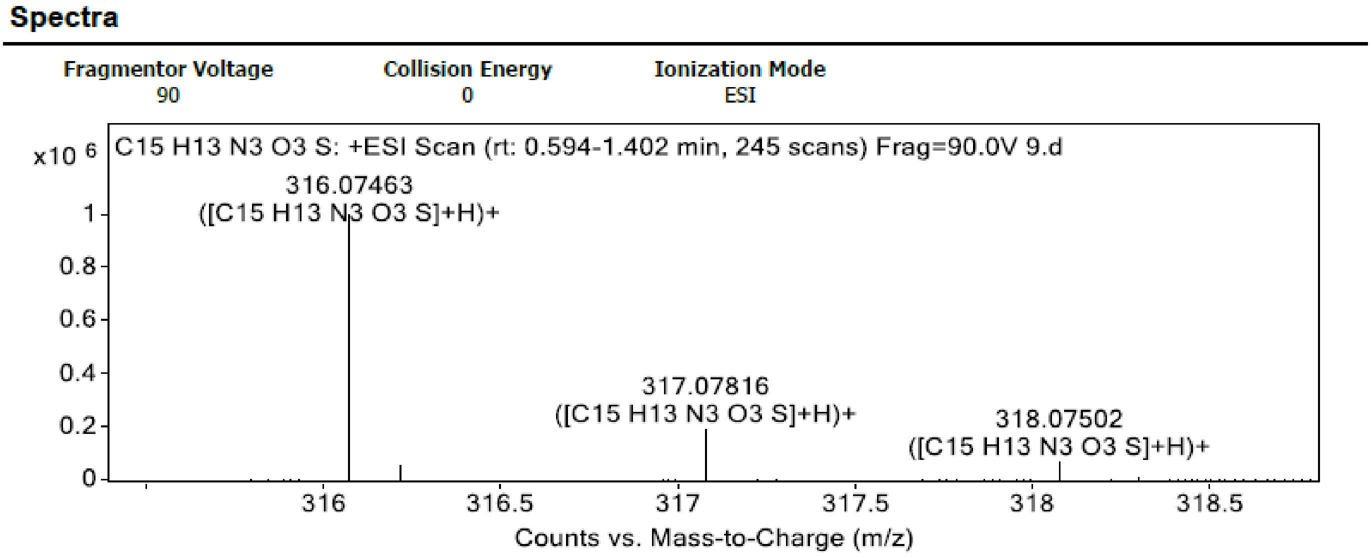

HRMS Spectrum of Compound 10

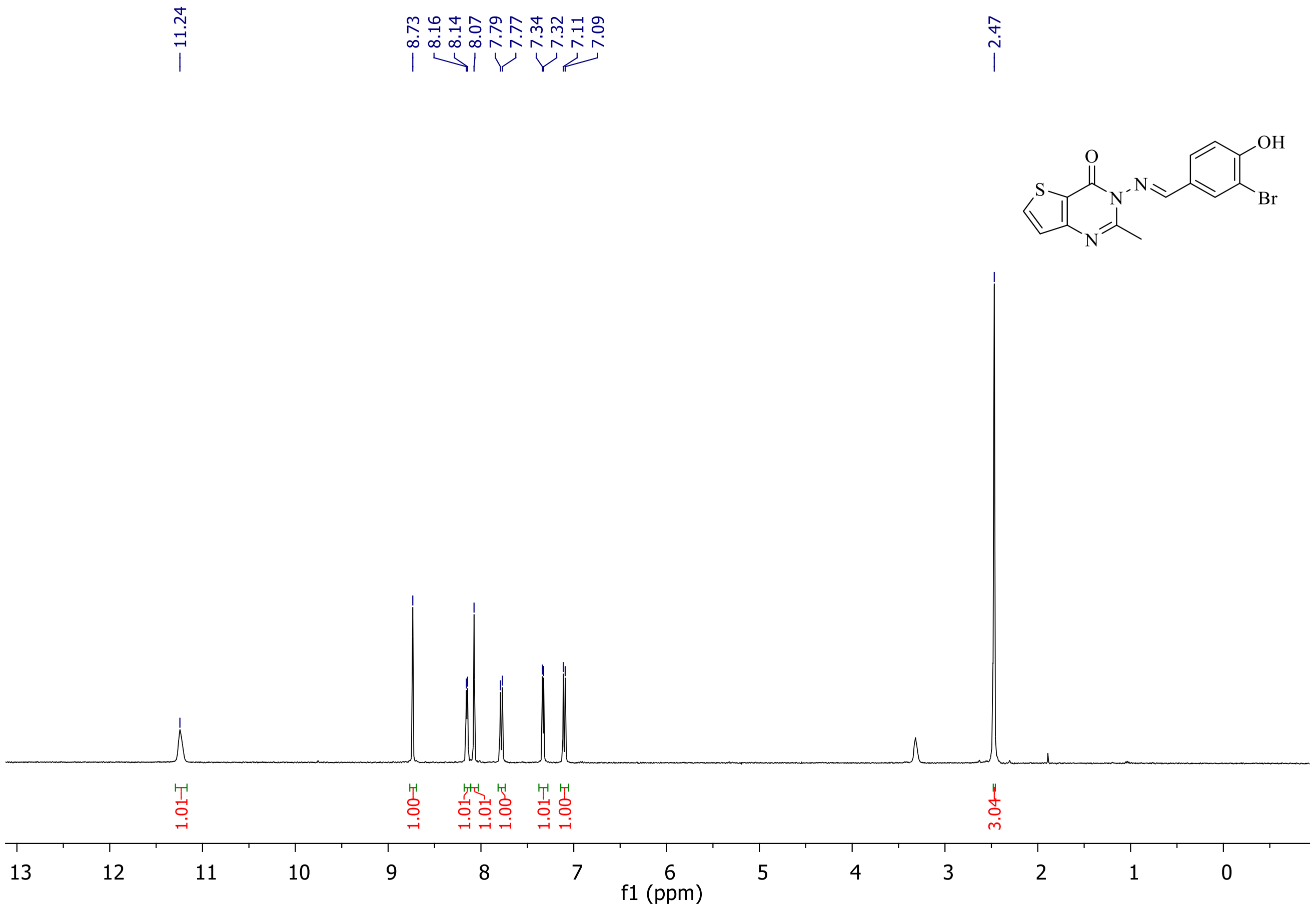

^{1}H NMR Spectrum of Compound 11

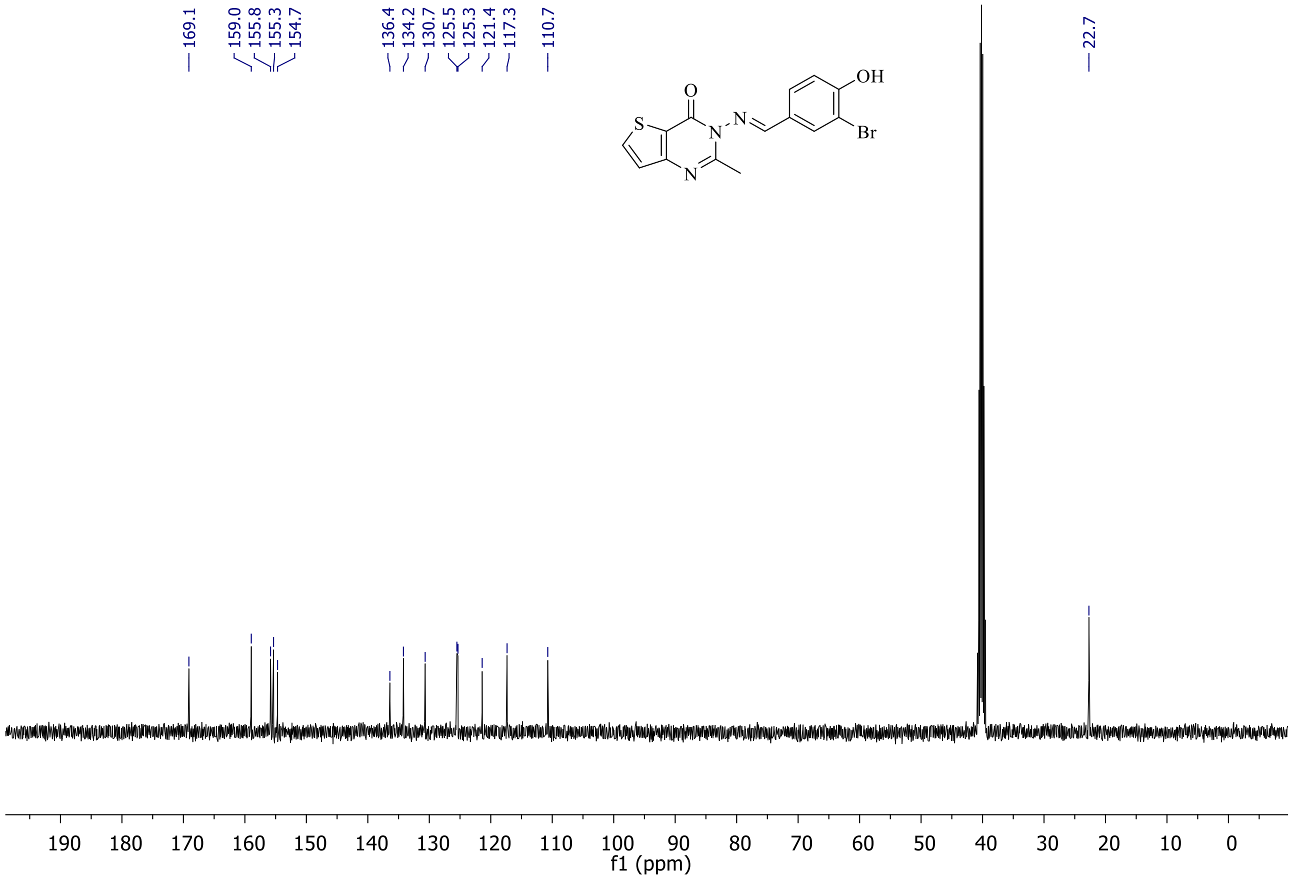

^{13}C NMR Spectrum of Compound 11

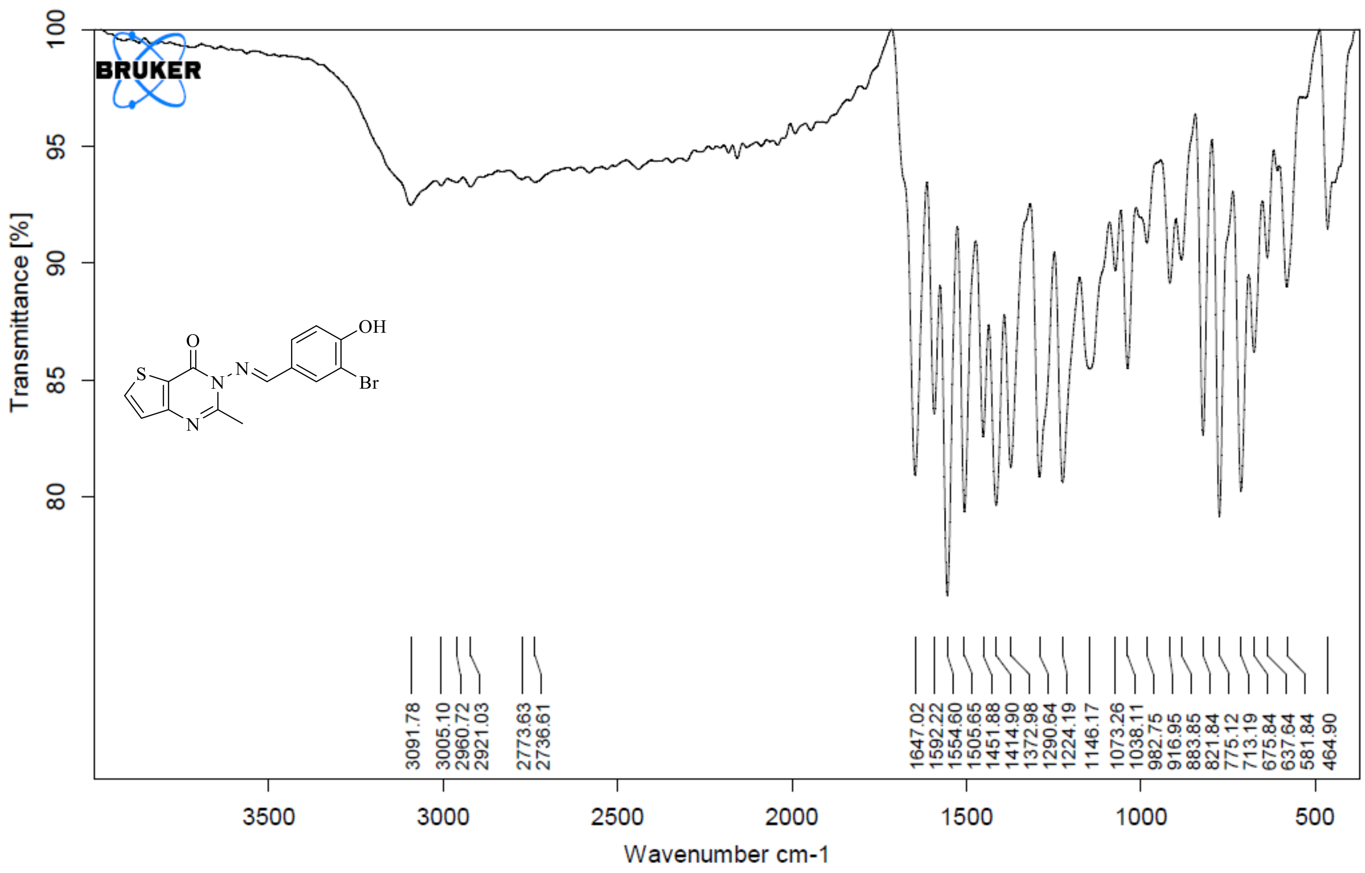

FTIR Spectrum of Compound 11

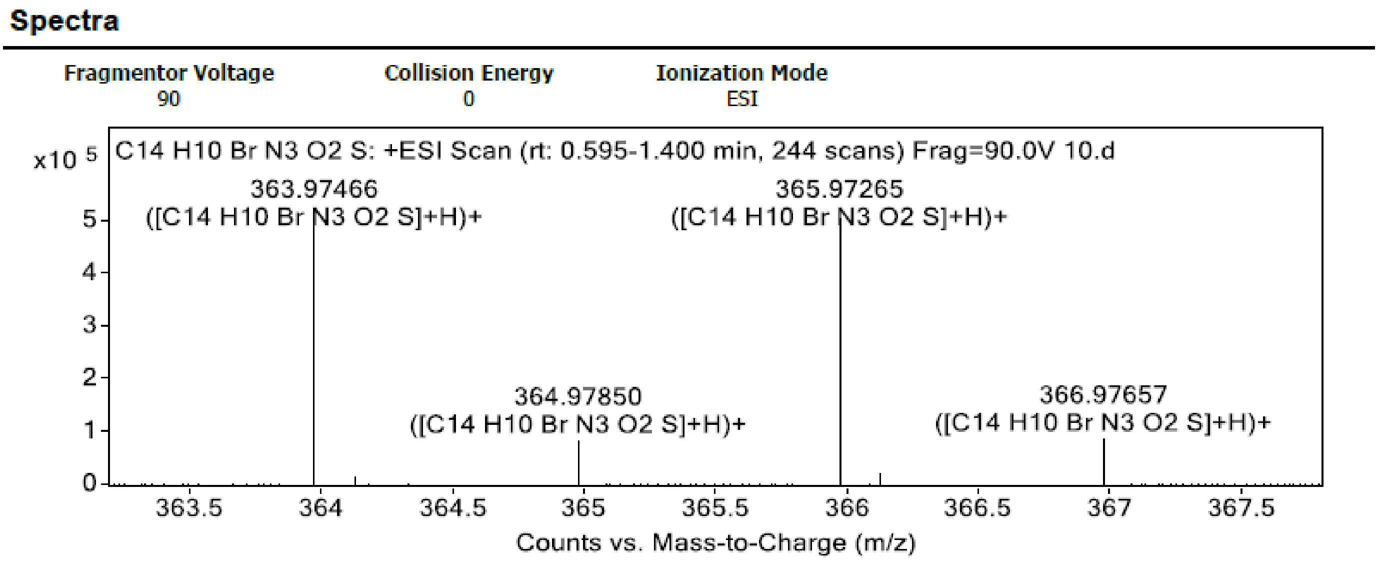

HRMS Spectrum of Compound 11

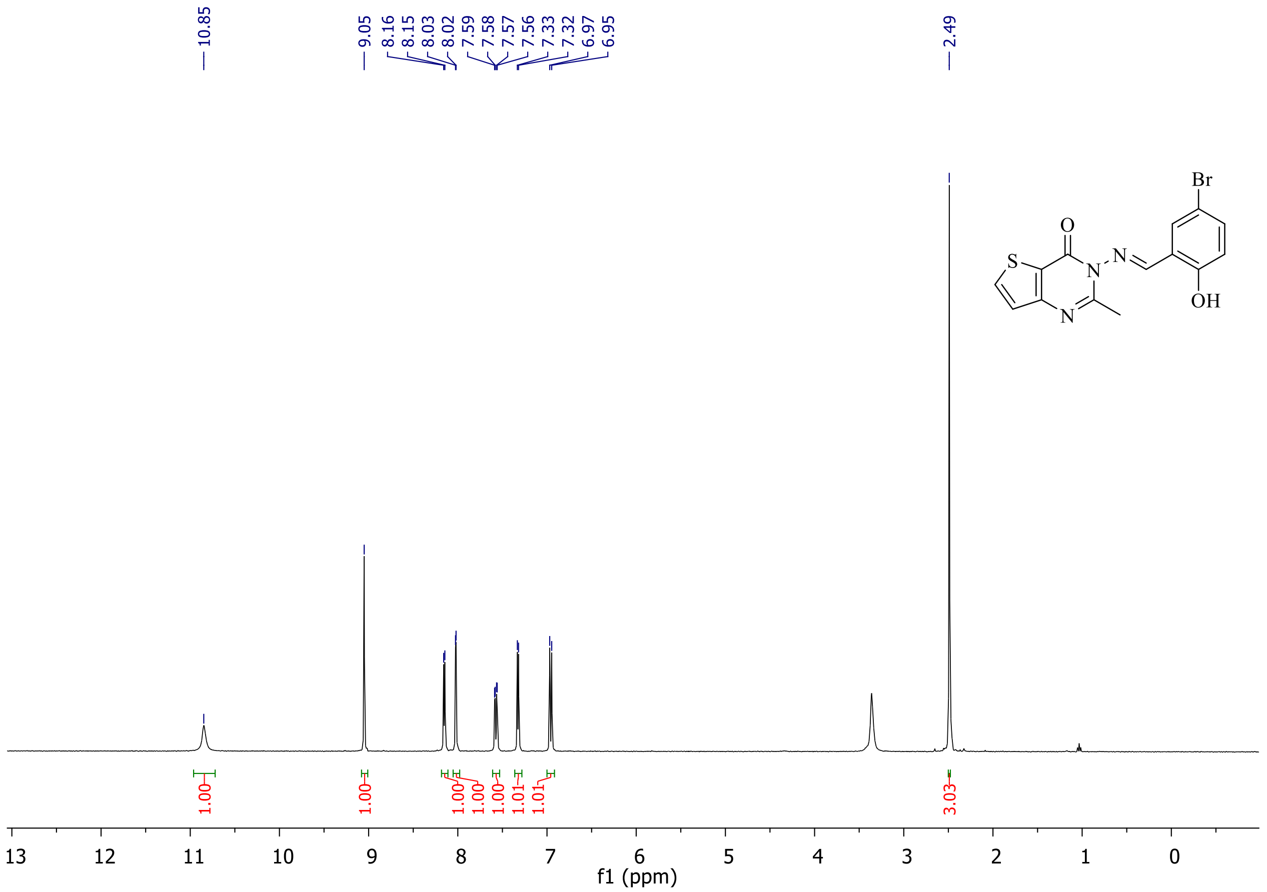

^{1}H NMR Spectrum of Compound 12

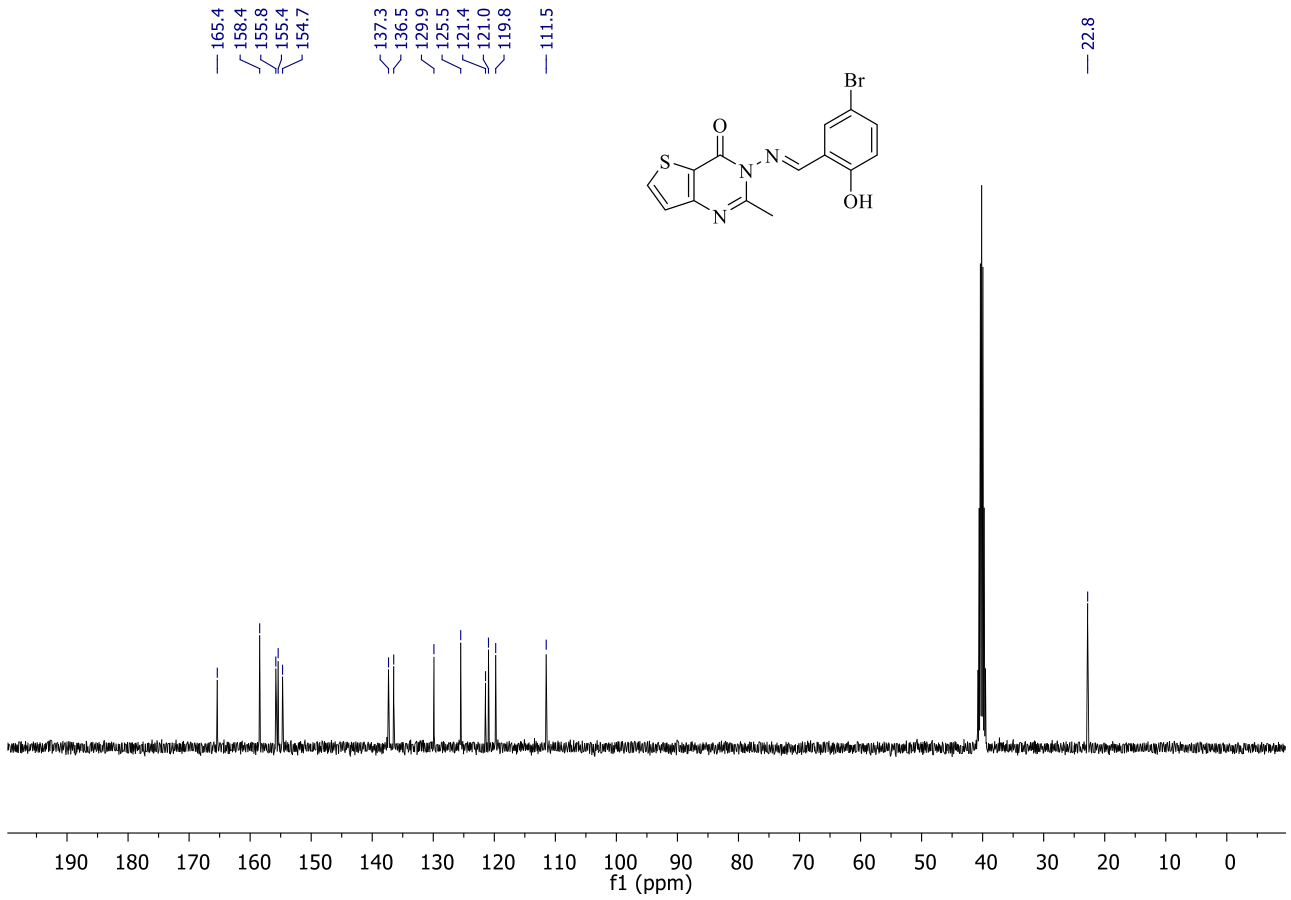

^{13}C NMR Spectrum of Compound 12

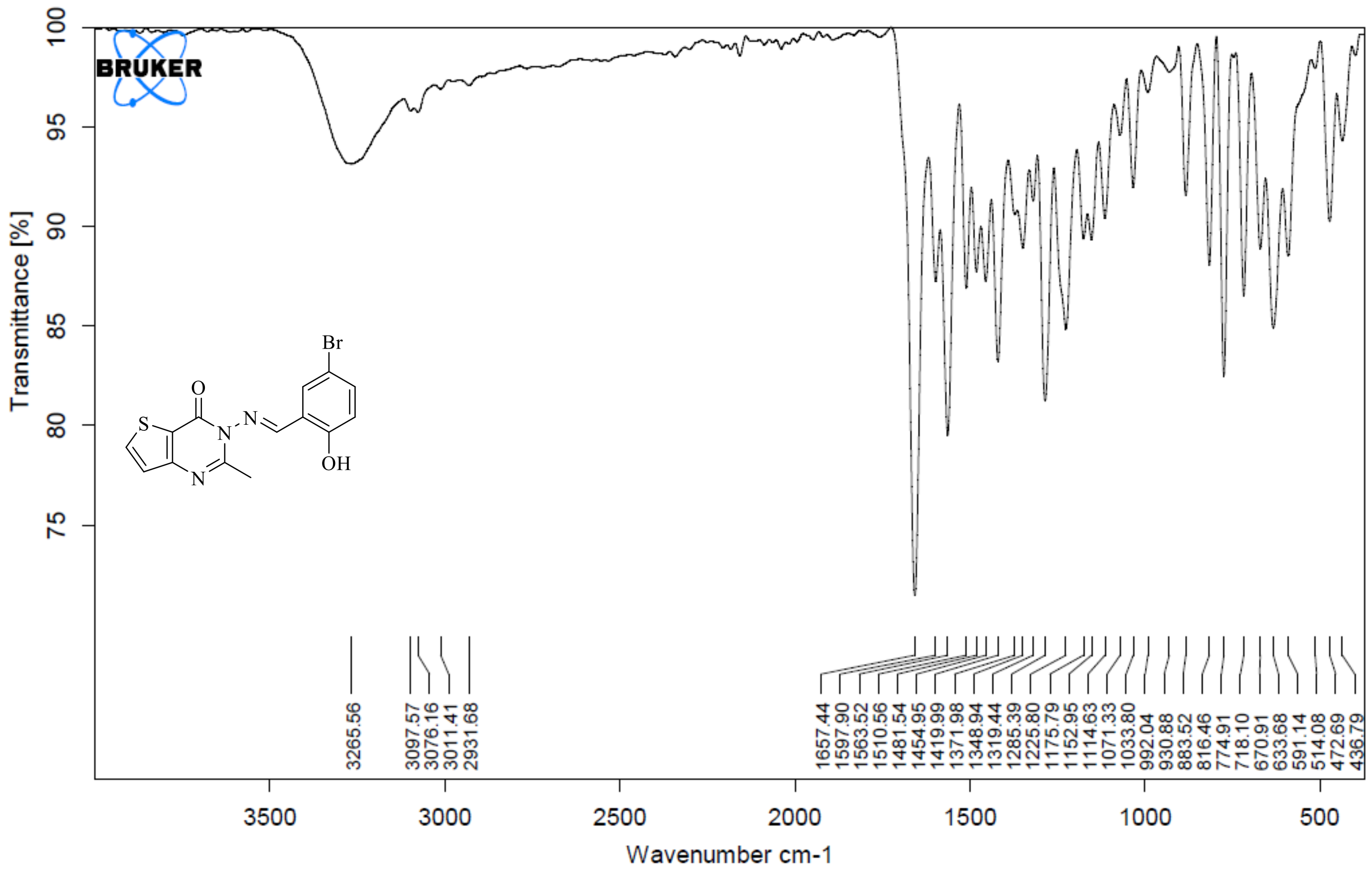

FTIR Spectrum of Compound 12

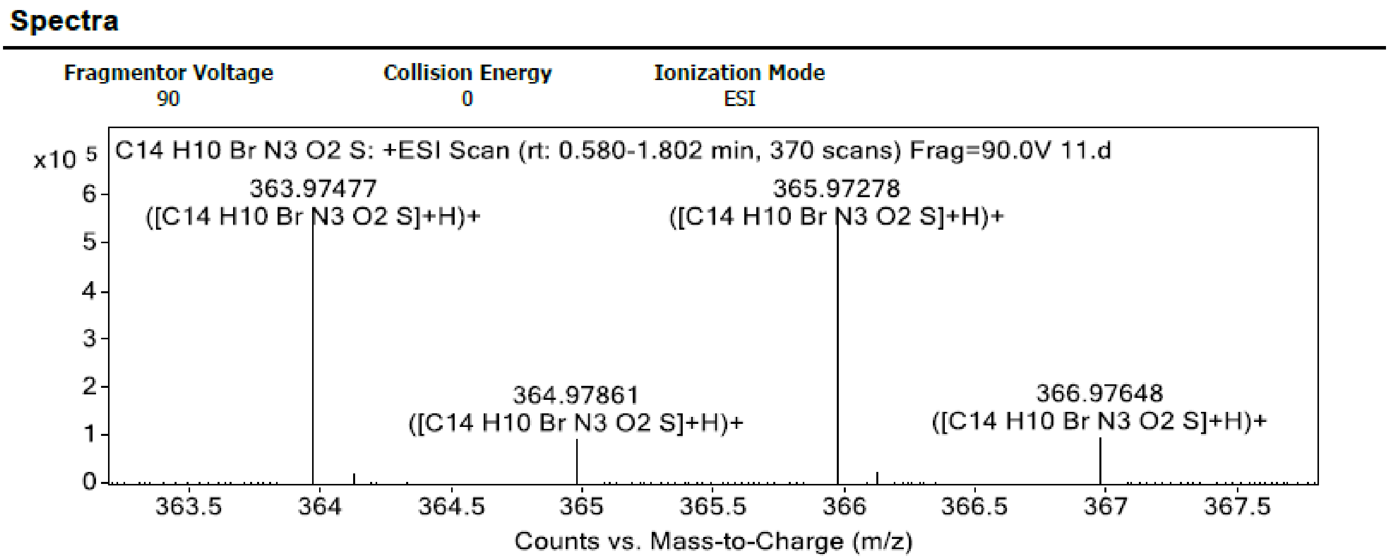

HRMS Spectrum of Compound 12

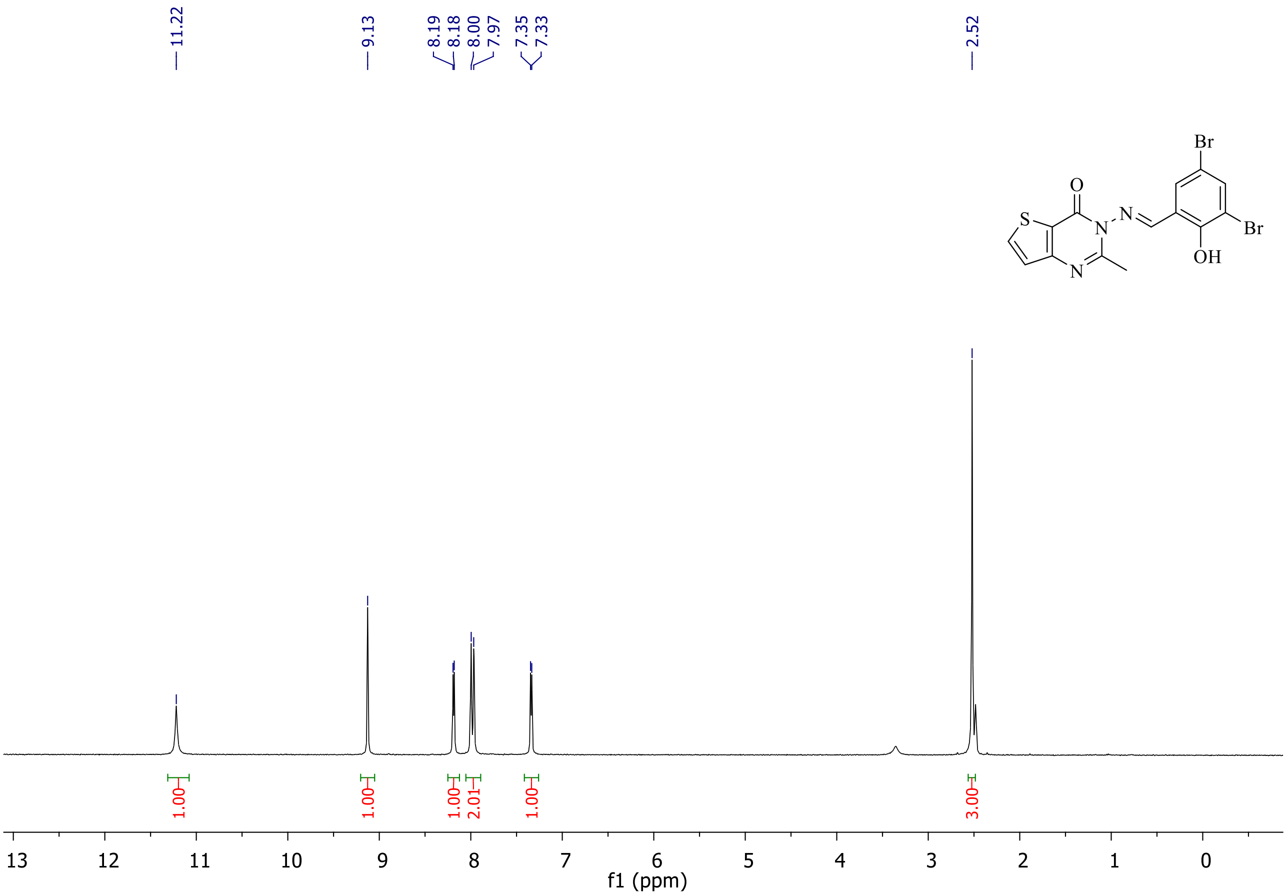

^{1}H NMR Spectrum of Compound 13

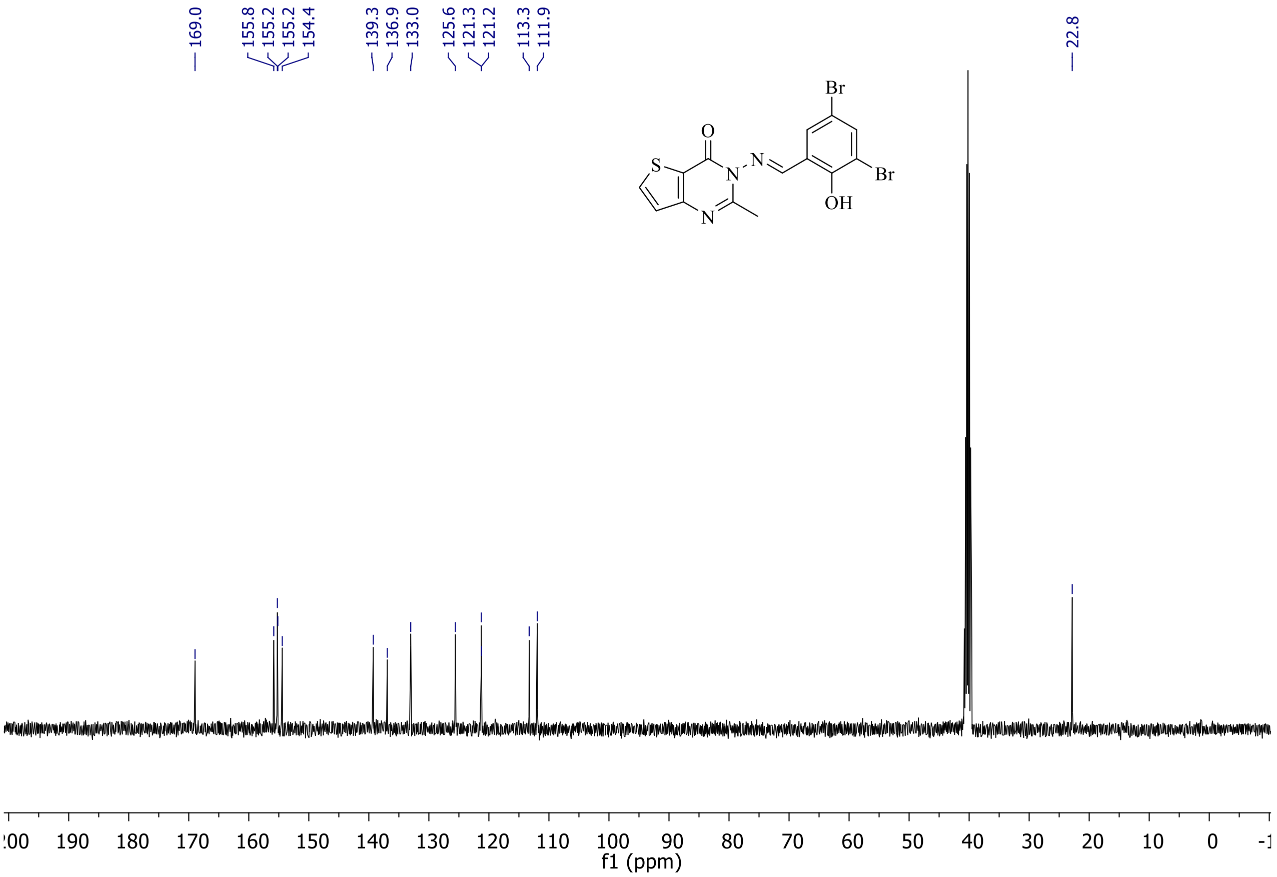

^{13}C NMR Spectrum of Compound 13

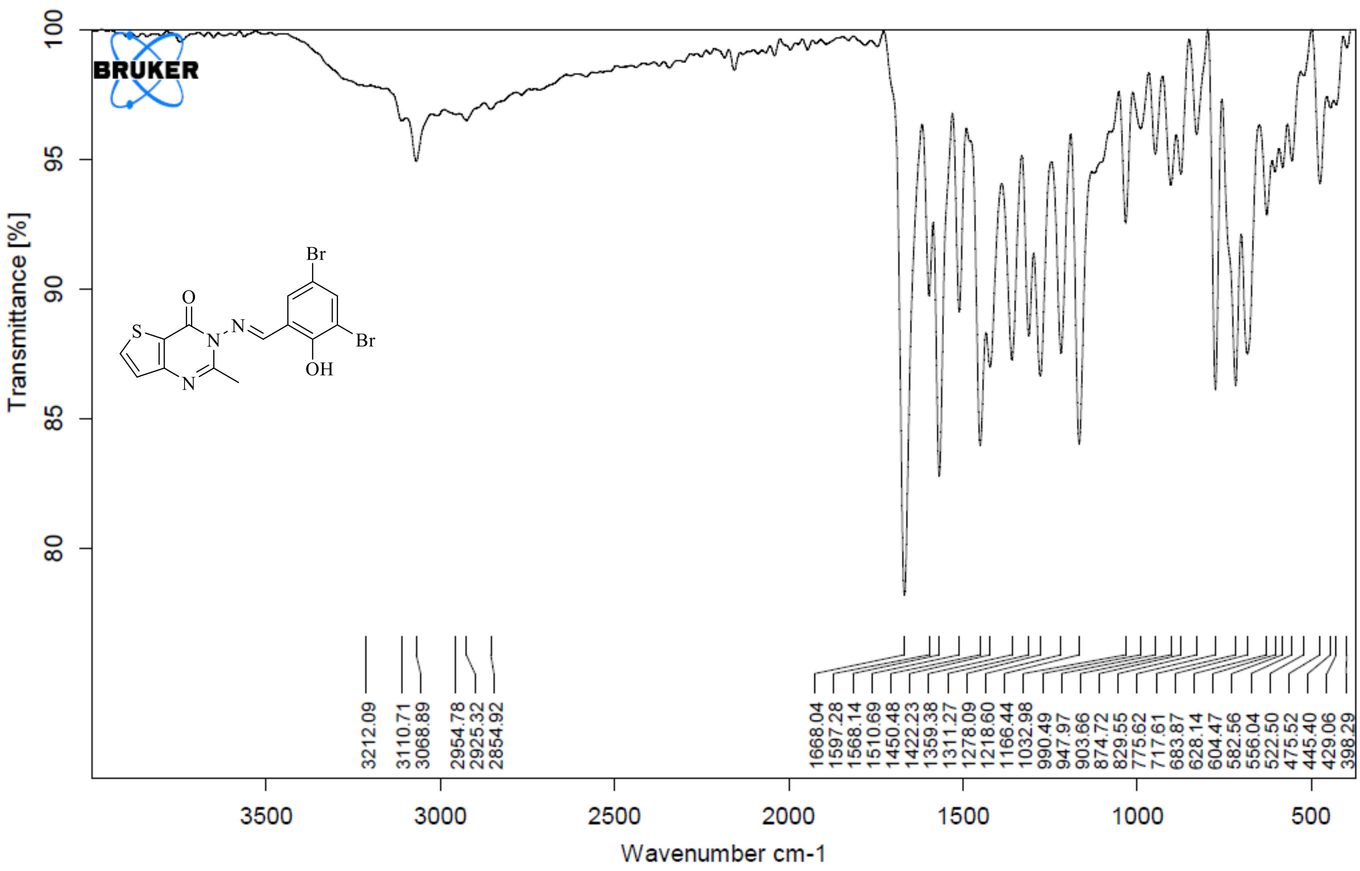

FTIR Spectrum of Compound 13

Spectra

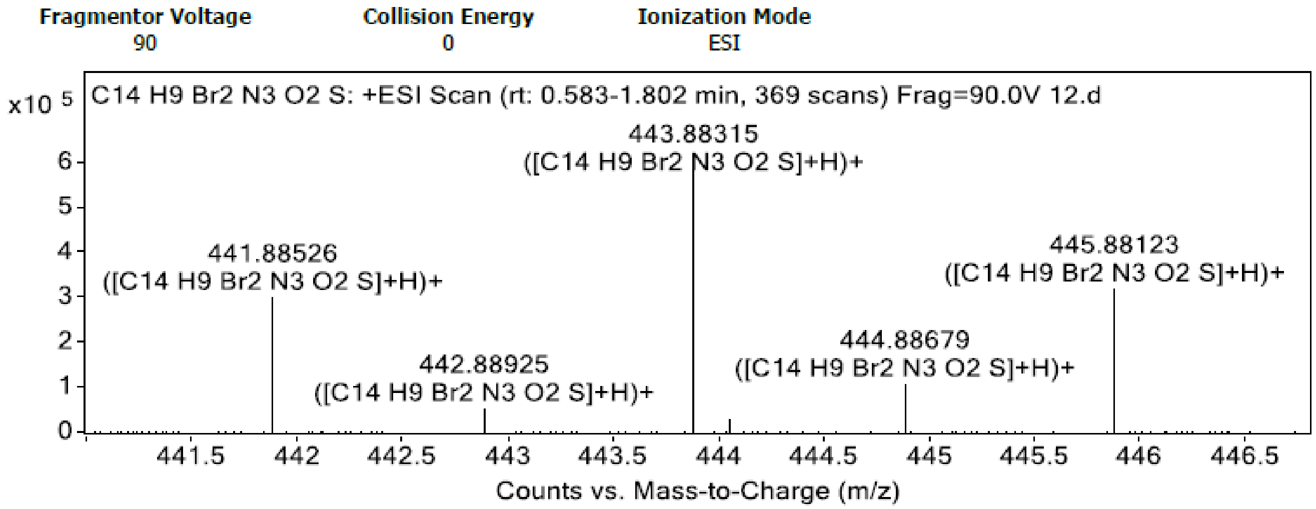

HRMS Spectrum of Compound 13

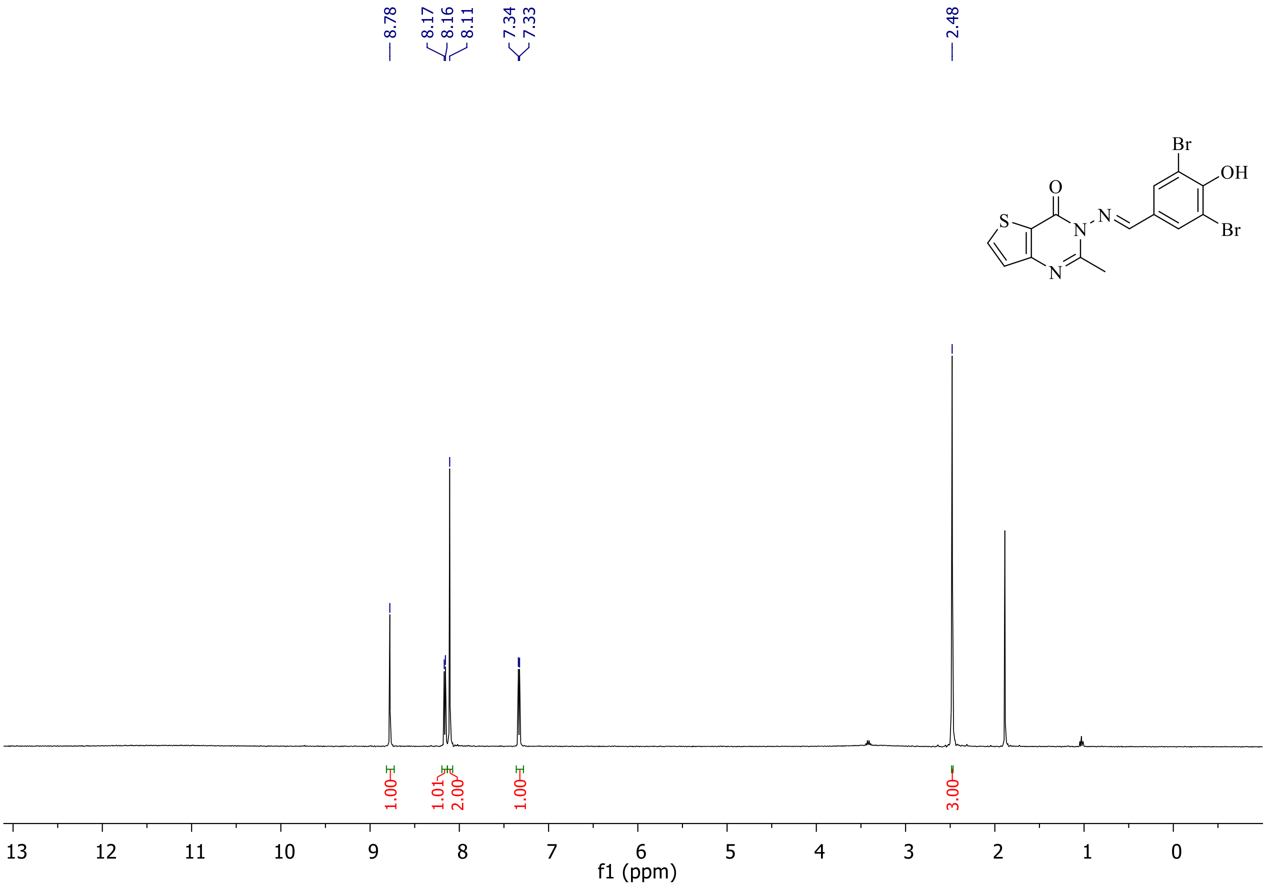

^{1}H NMR Spectrum of Compound 14

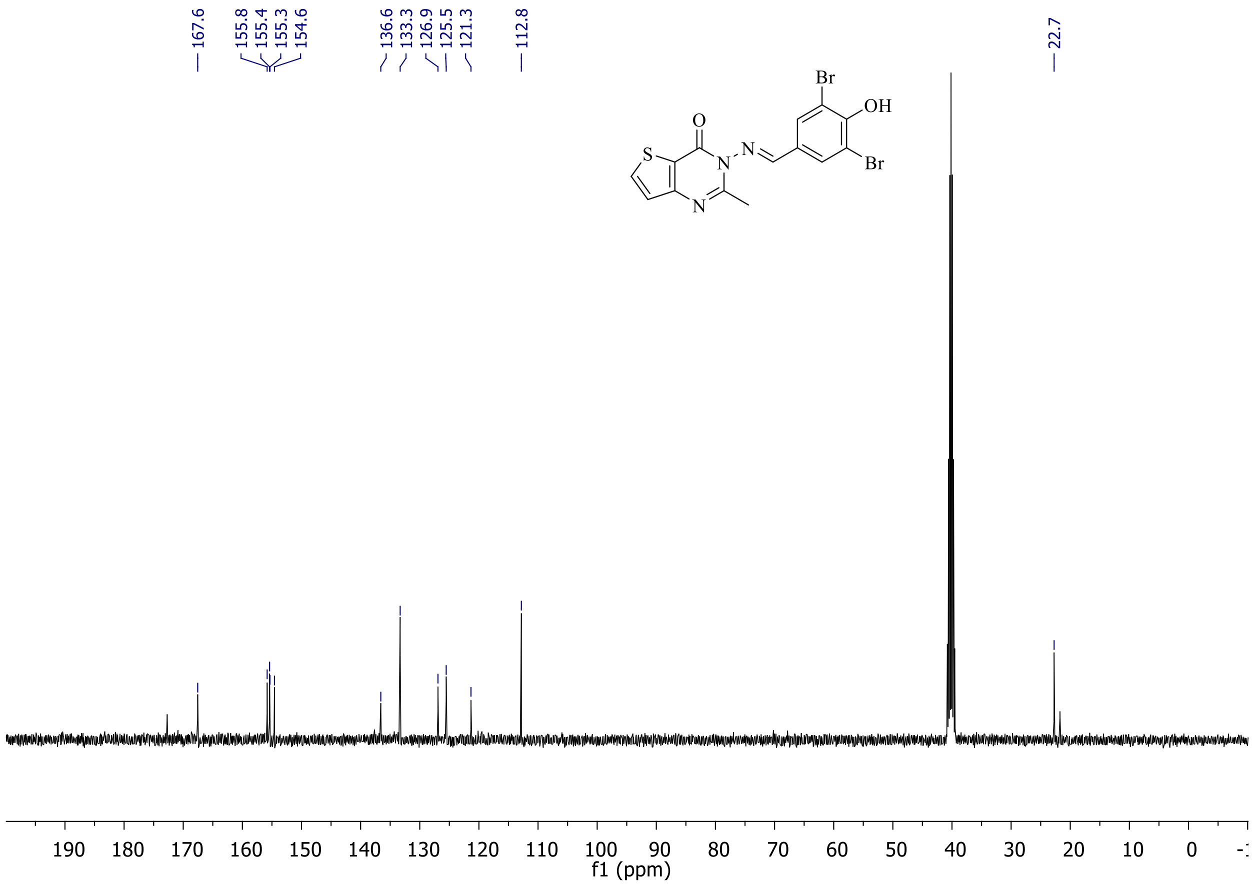

^{13}C NMR Spectrum of Compound 14

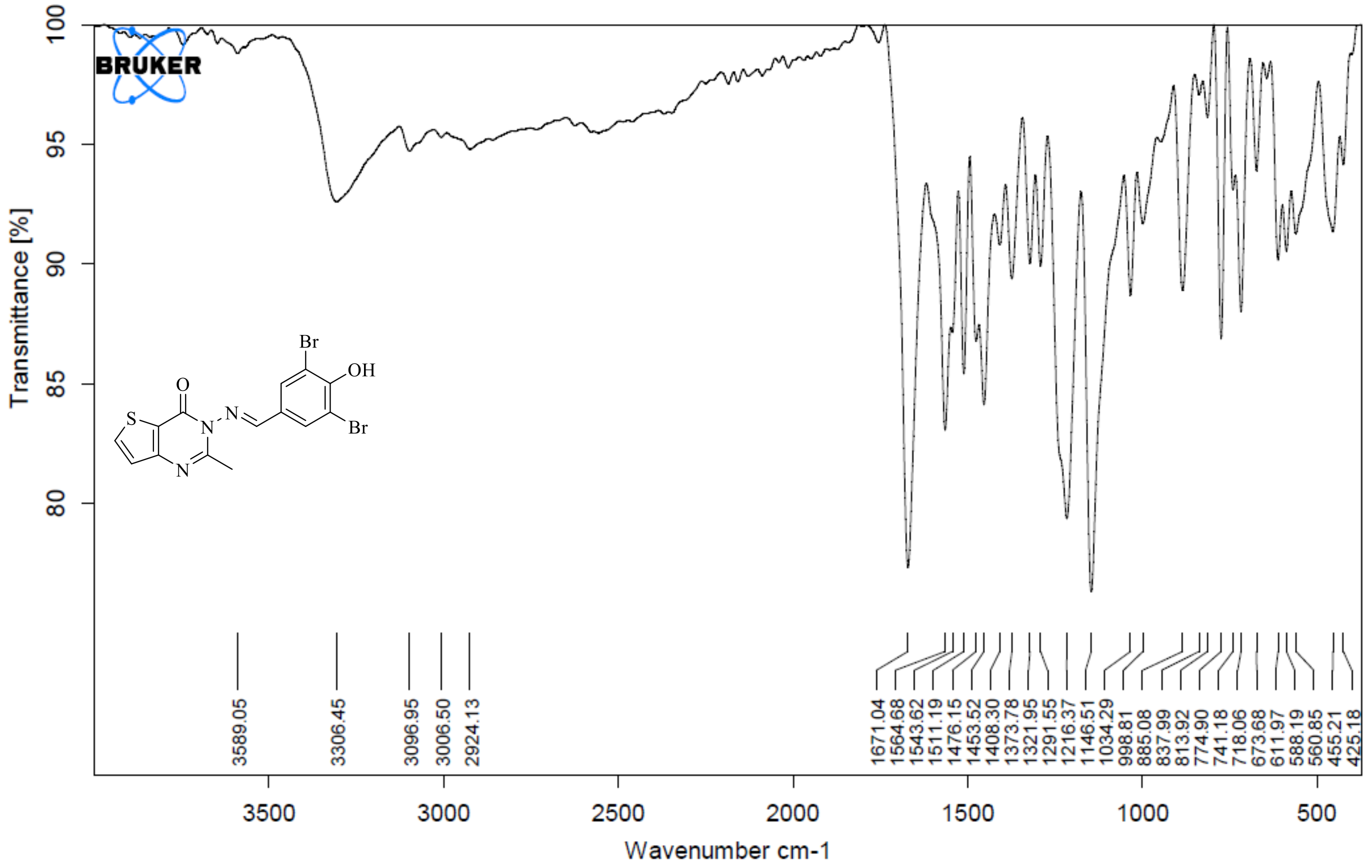

FTIR Spectrum of Compound 14

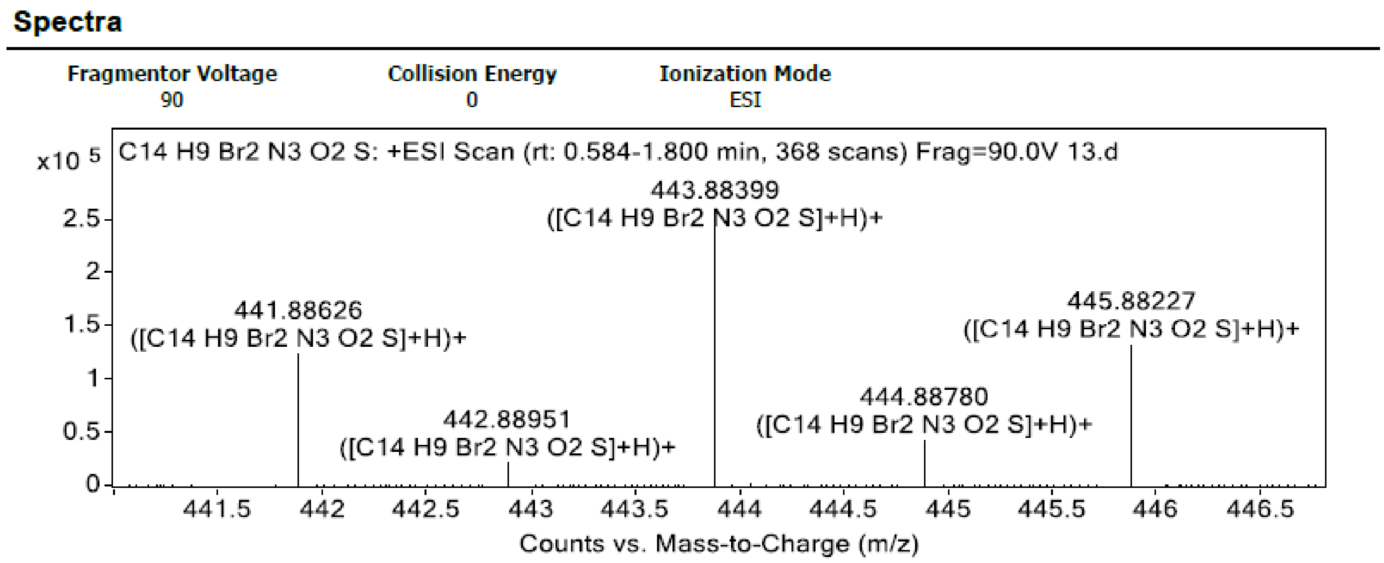

HRMS Spectrum of Compound 14

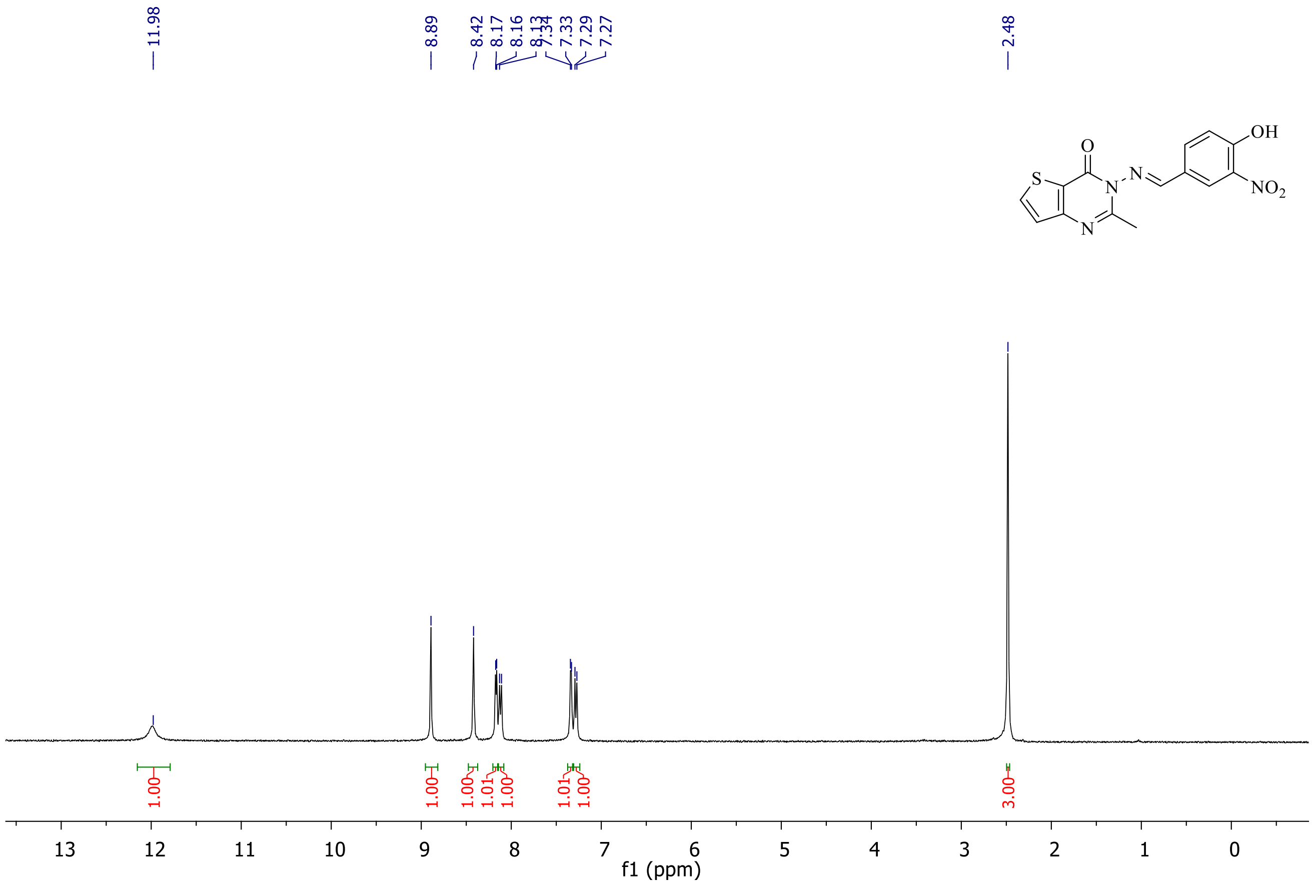

^{1}H NMR Spectrum of Compound 15

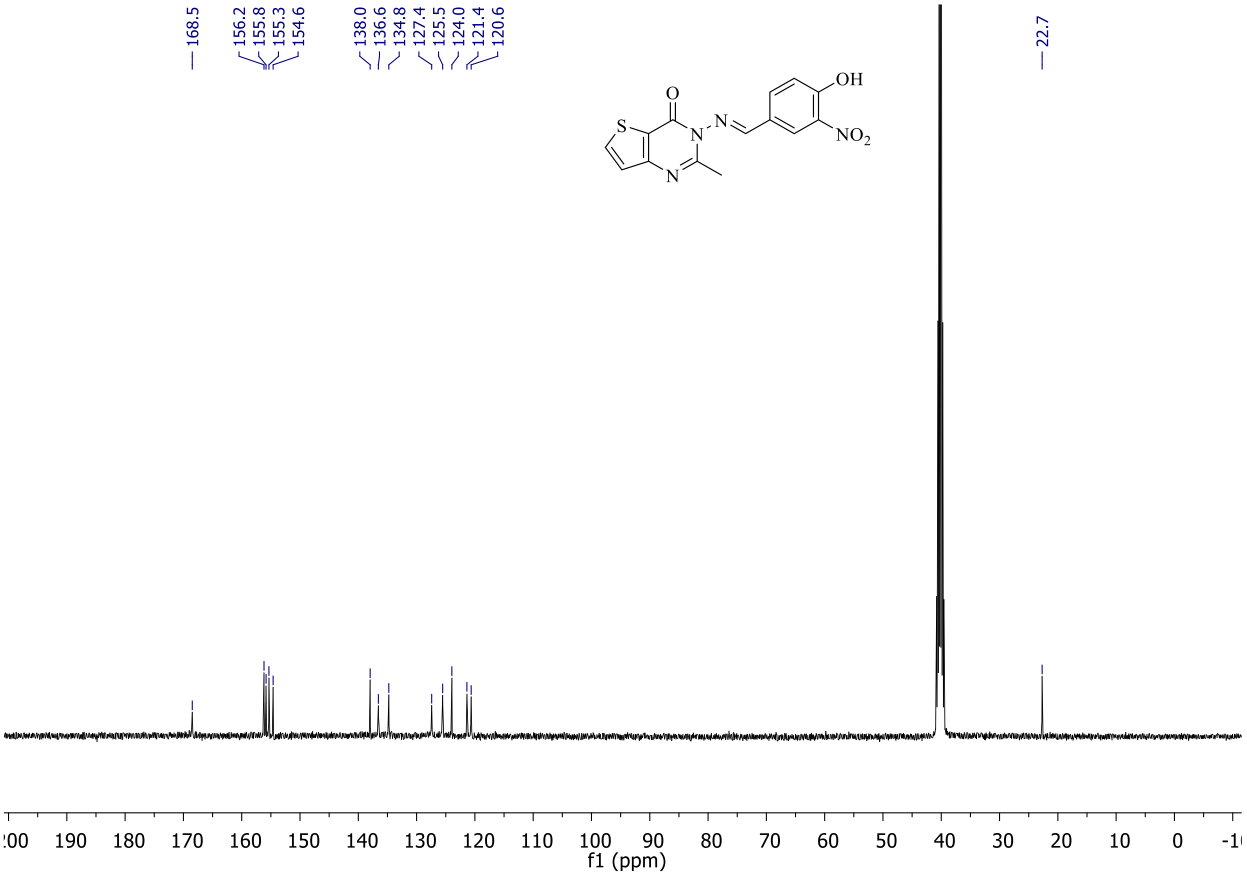

^{13}C NMR Spectrum of Compound 15

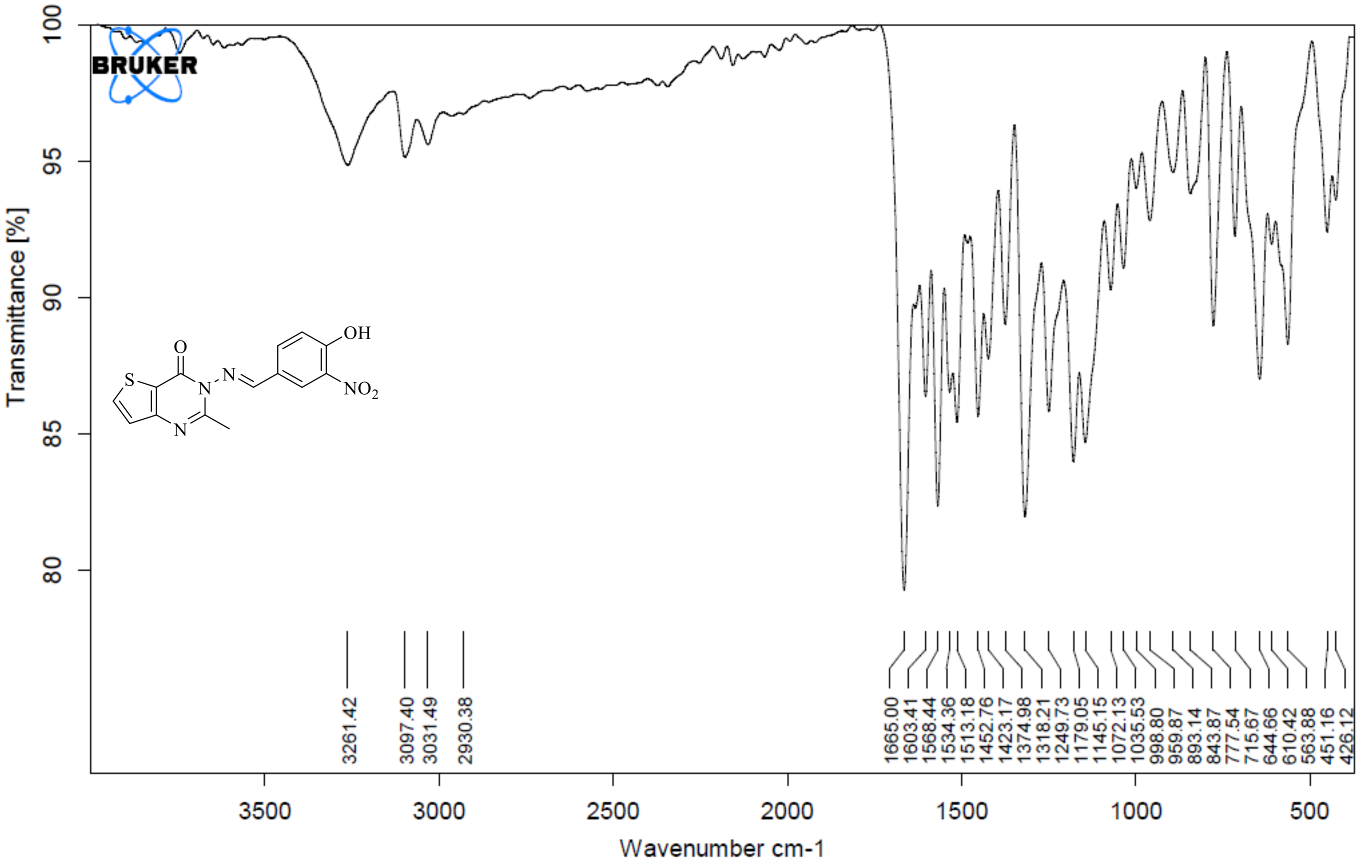

FTIR Spectrum of Compound 15

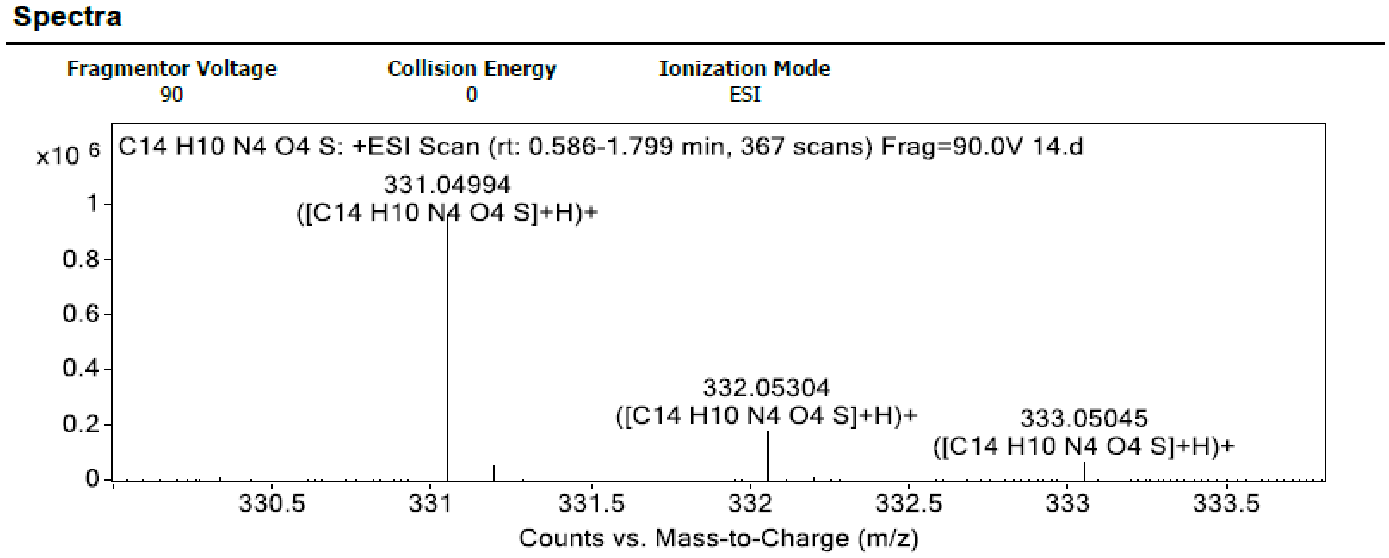

HRMS Spectrum of Compound 15

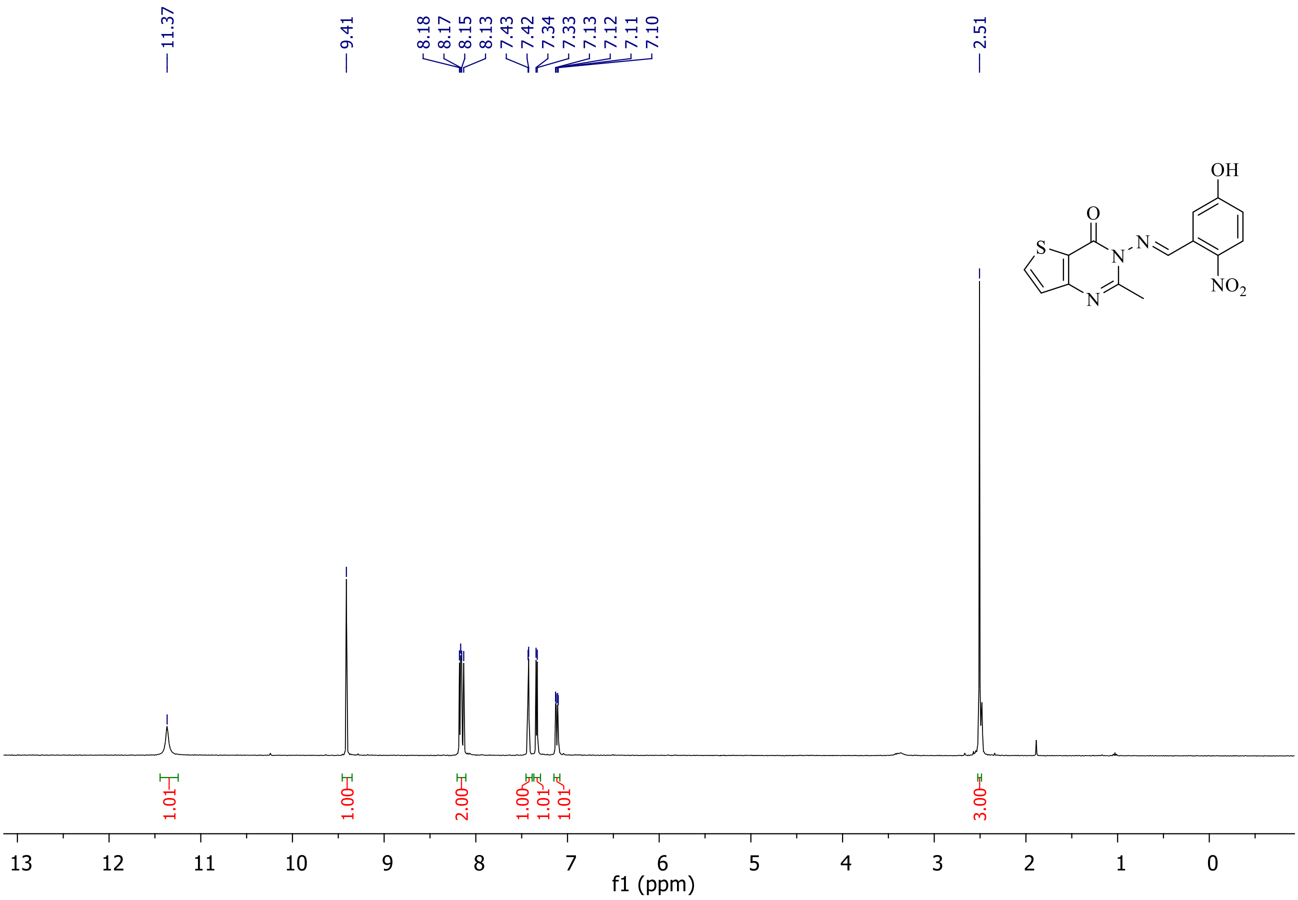

^{1}H NMR Spectrum of Compound 16

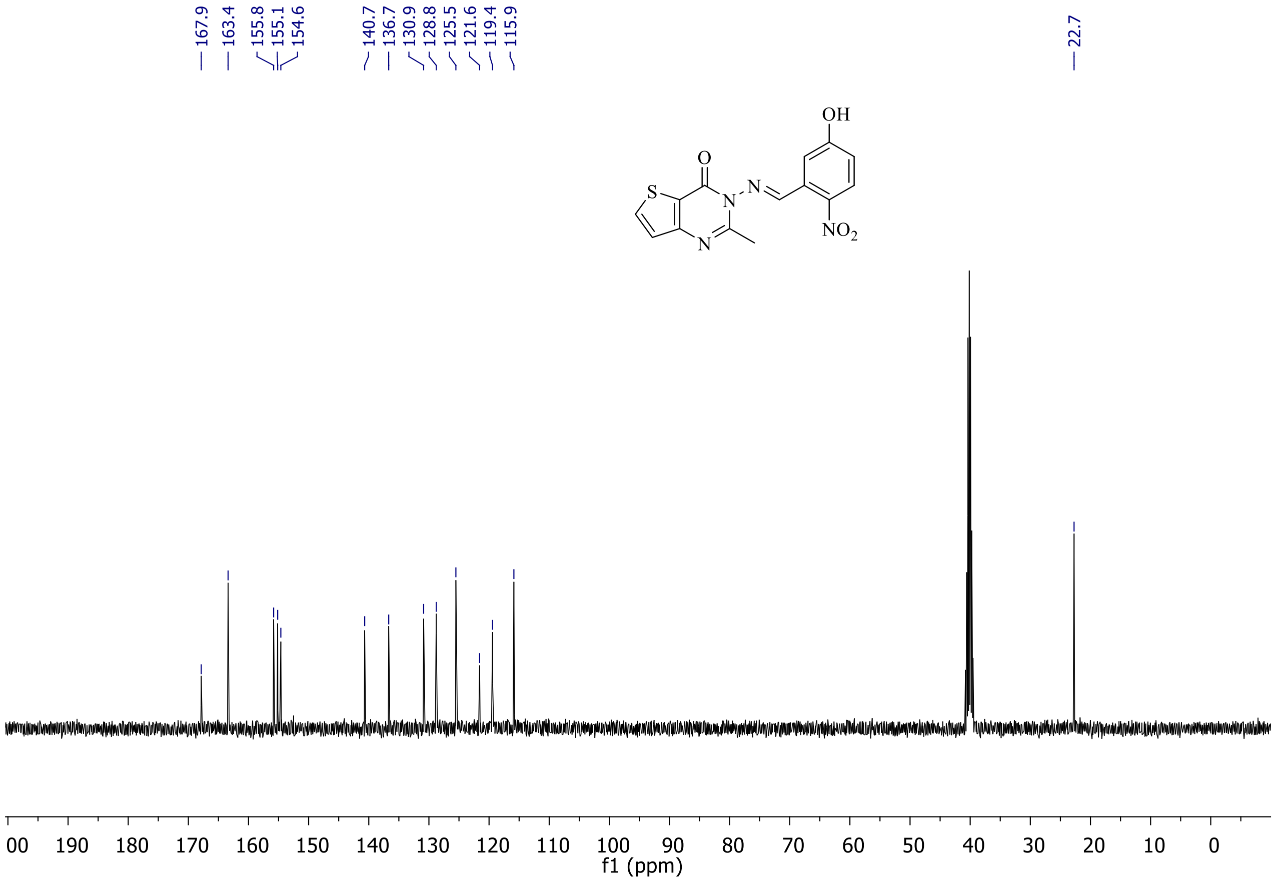

^{13}C NMR Spectrum of Compound 16

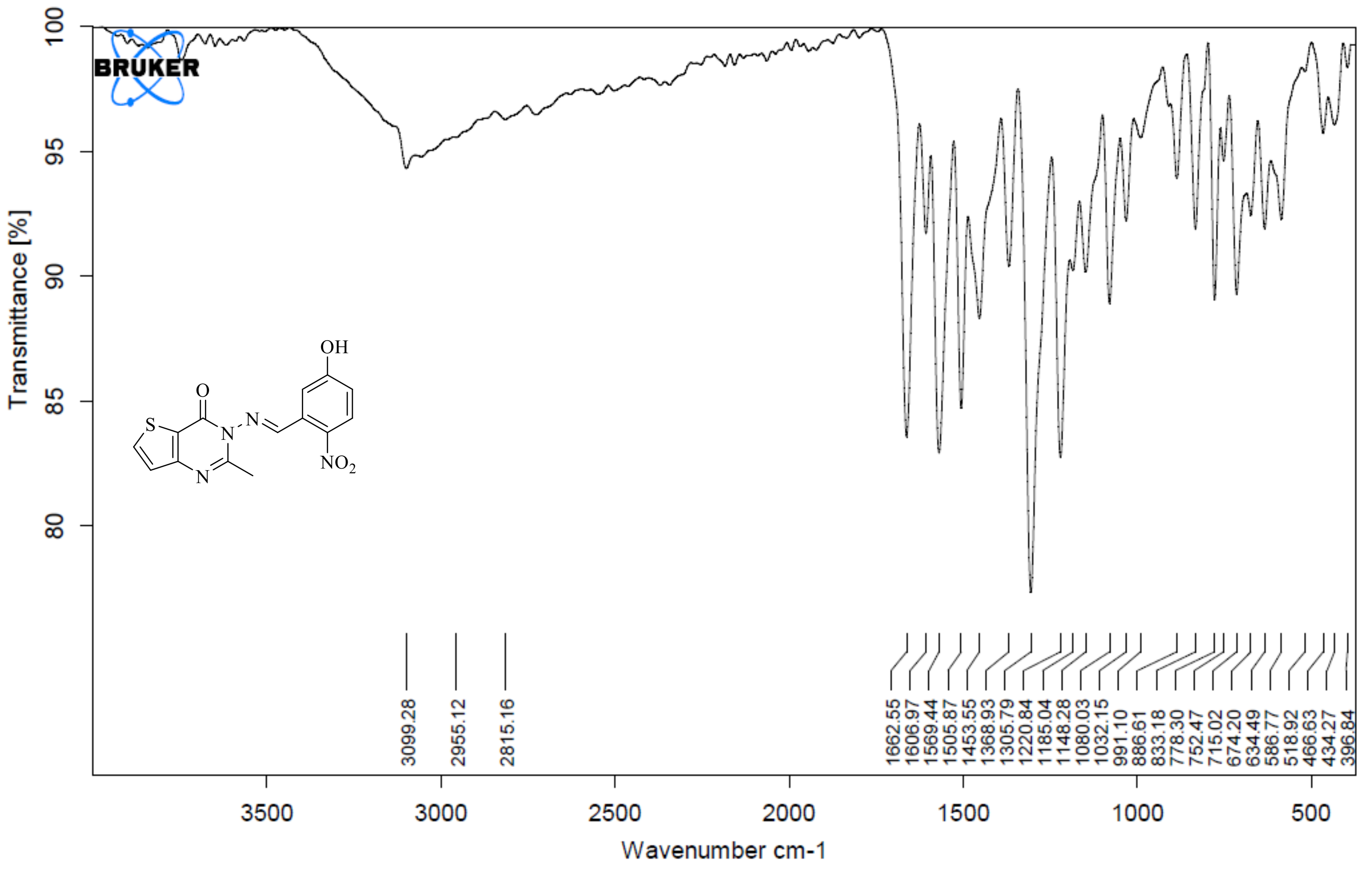

FTIR Spectrum of Compound 16

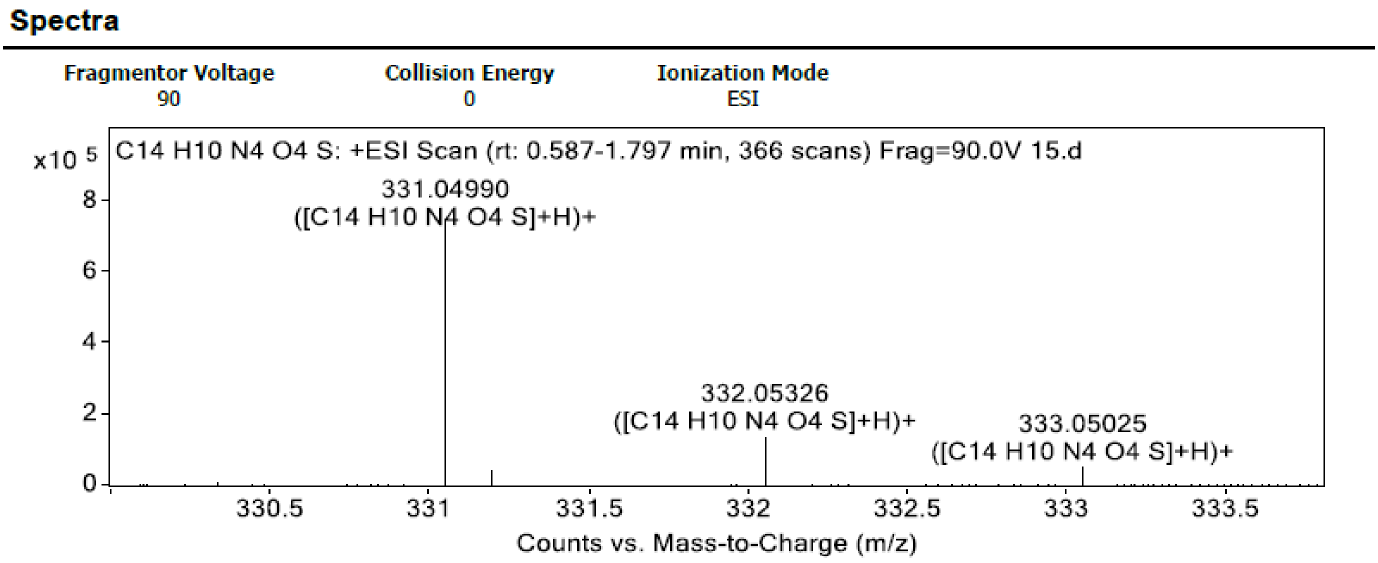

HRMS Spectrum of Compound 16

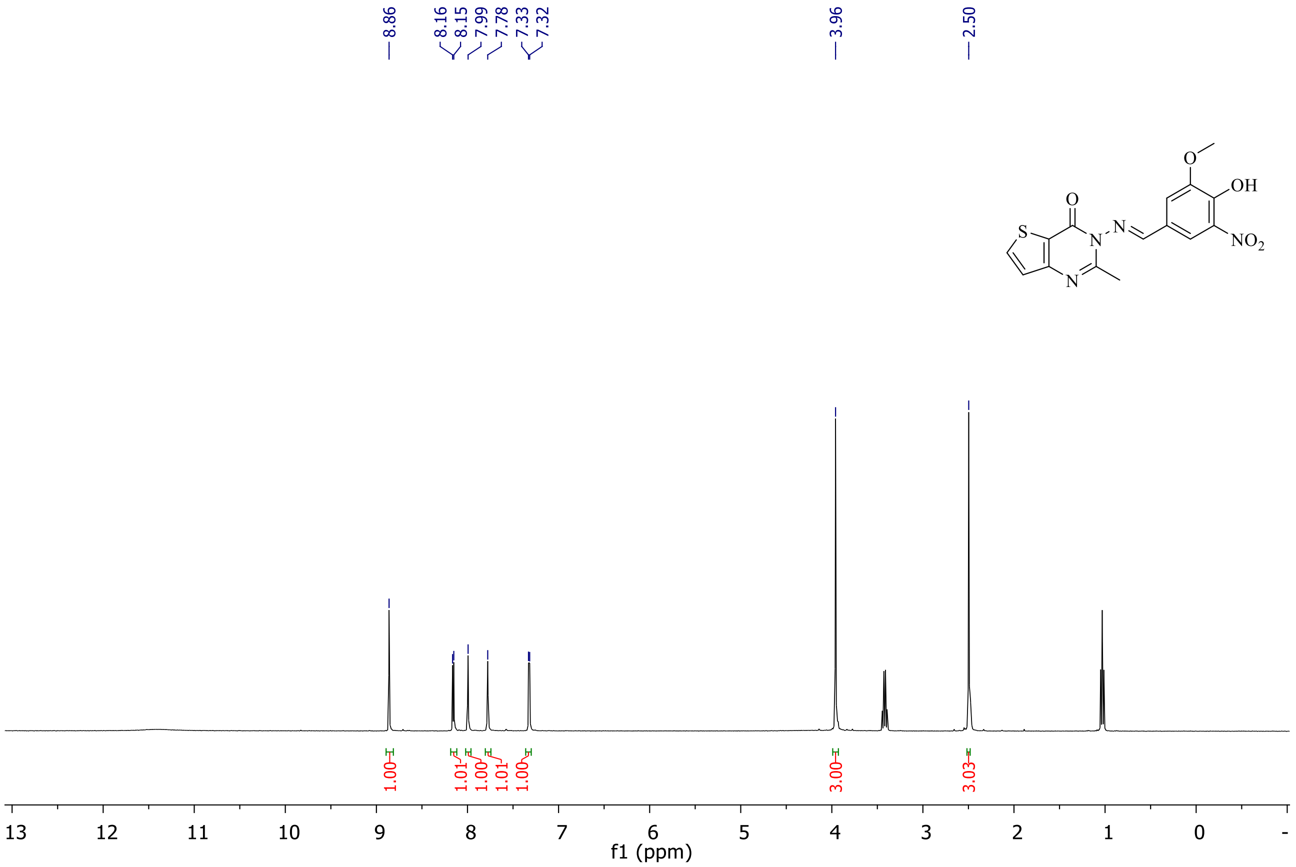

^{1}H NMR Spectrum of Compound 17

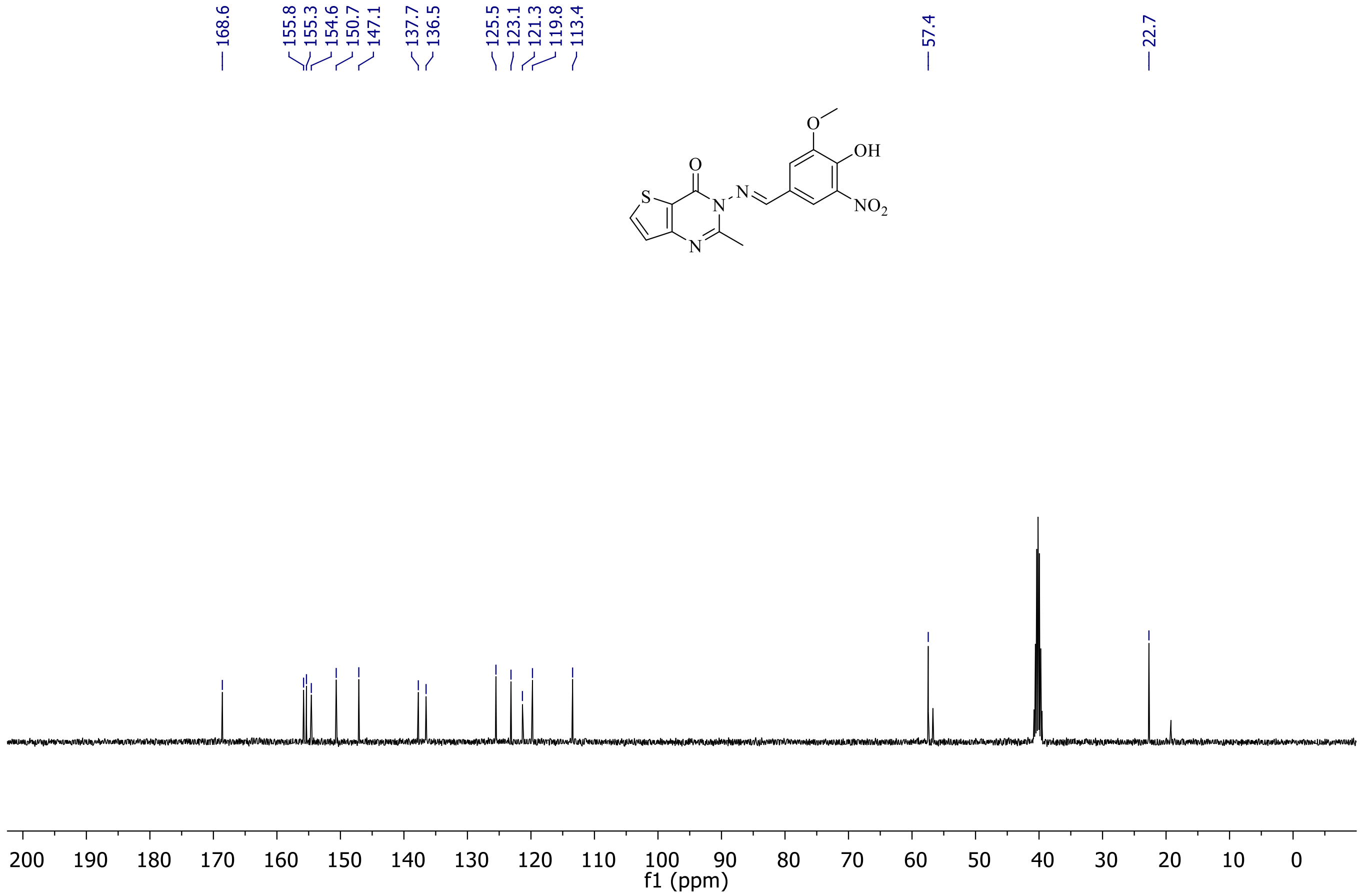

^{13}C NMR Spectrum of Compound 17

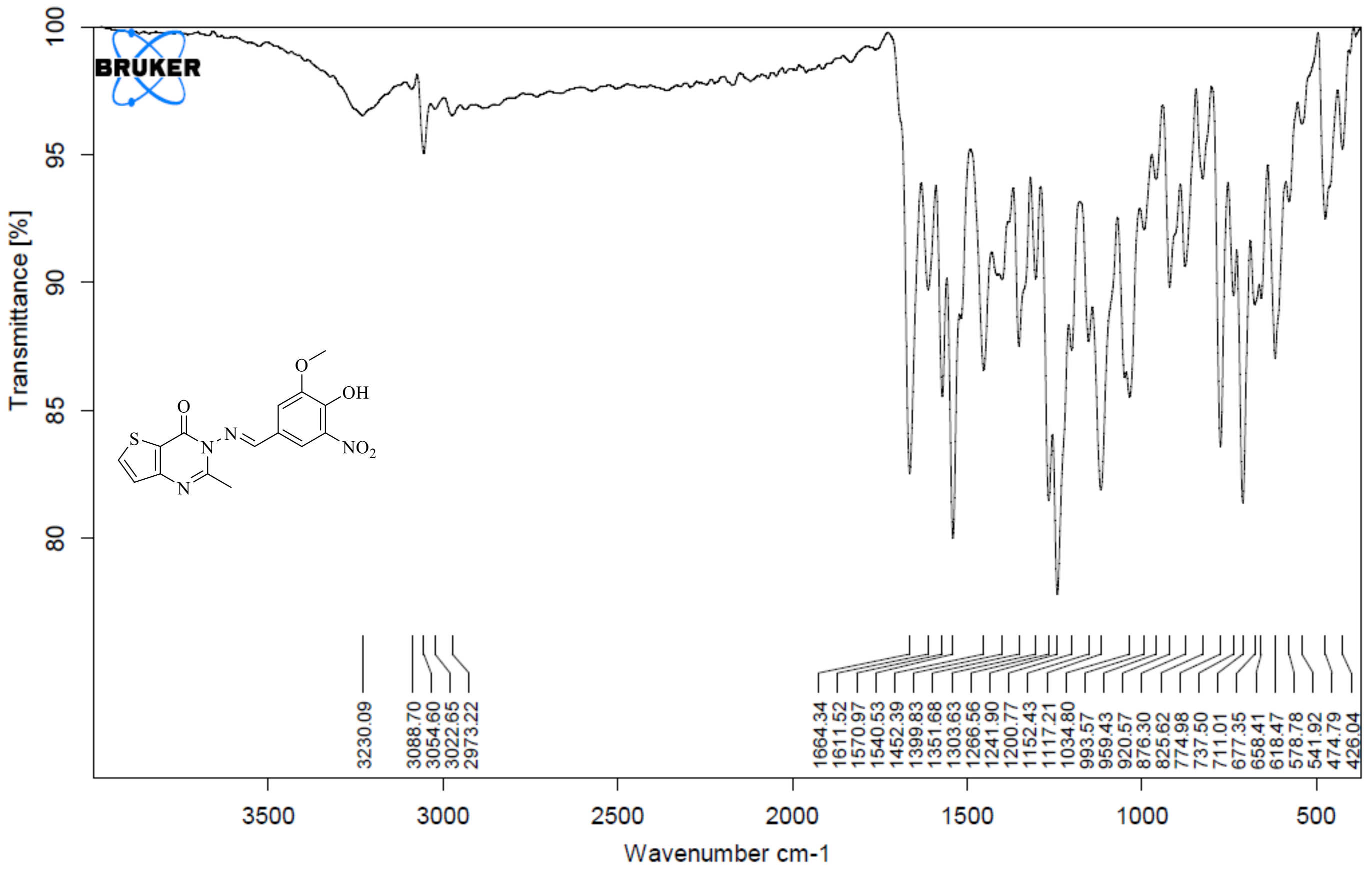

FTIR Spectrum of Compound 17

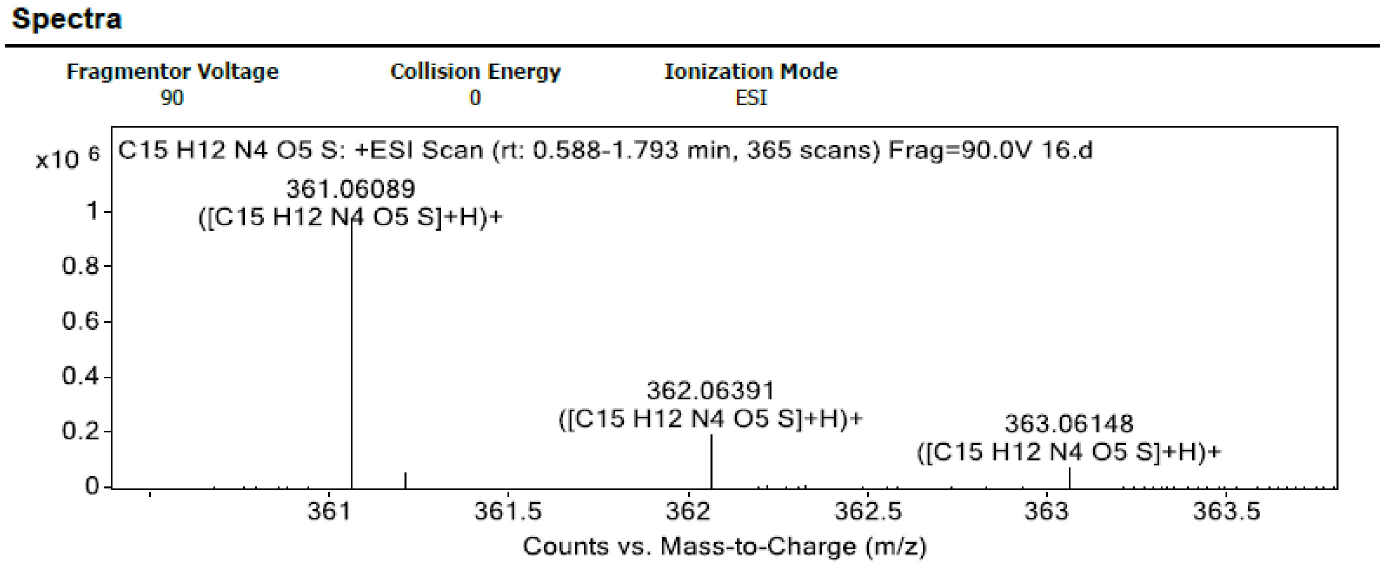

HRMS Spectrum of Compound 17

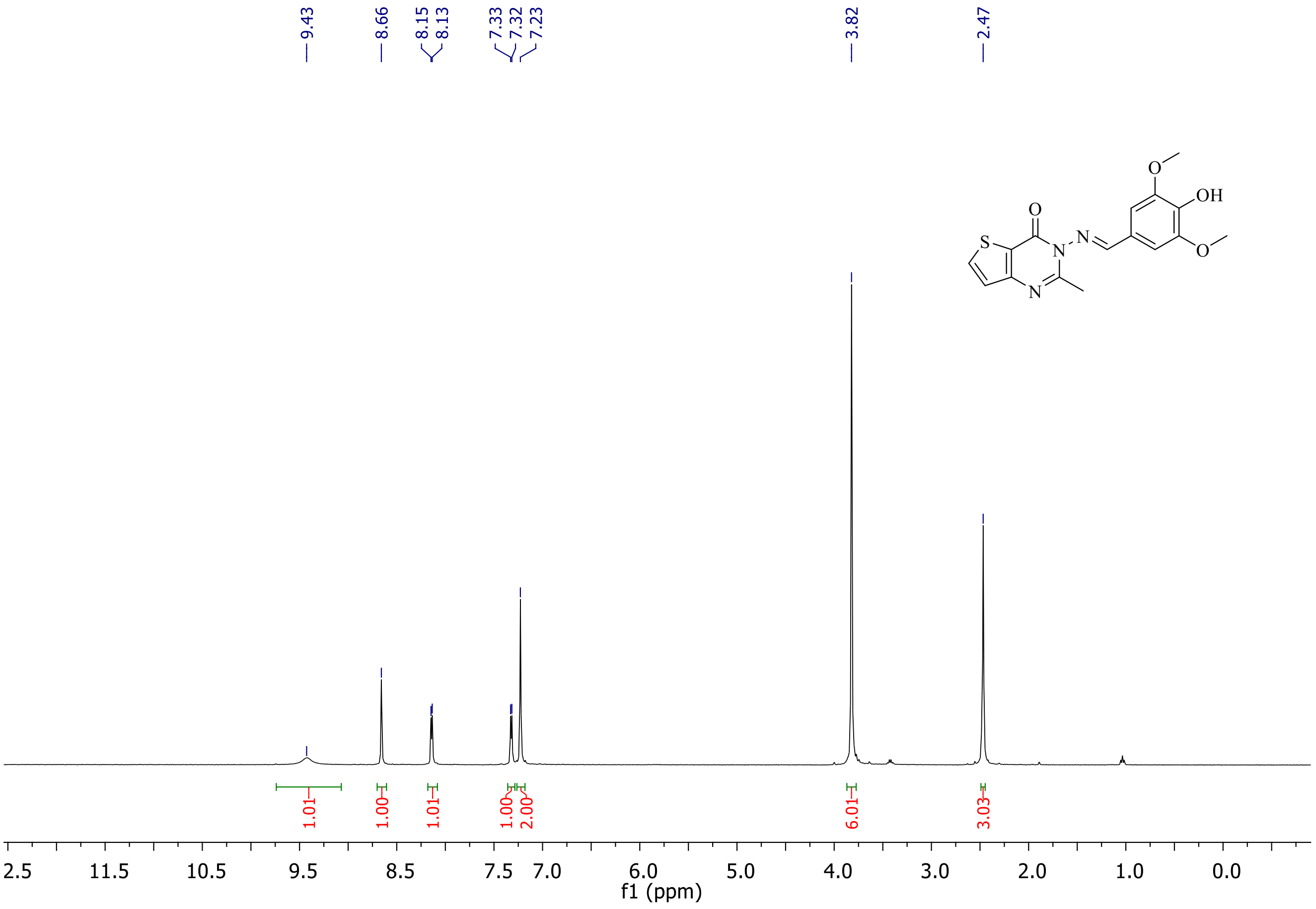

^{1}H NMR Spectrum of Compound 18

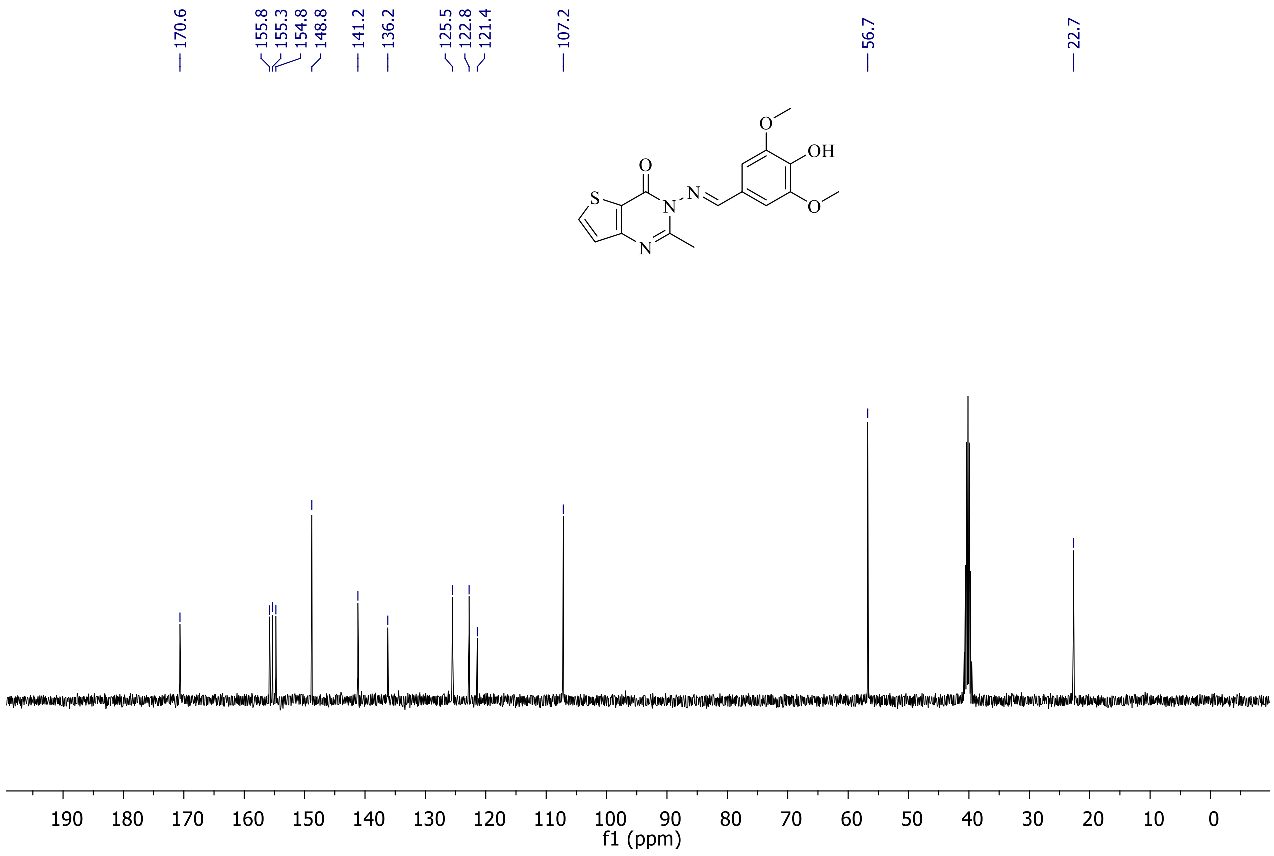

^{13}C NMR Spectrum of Compound 18

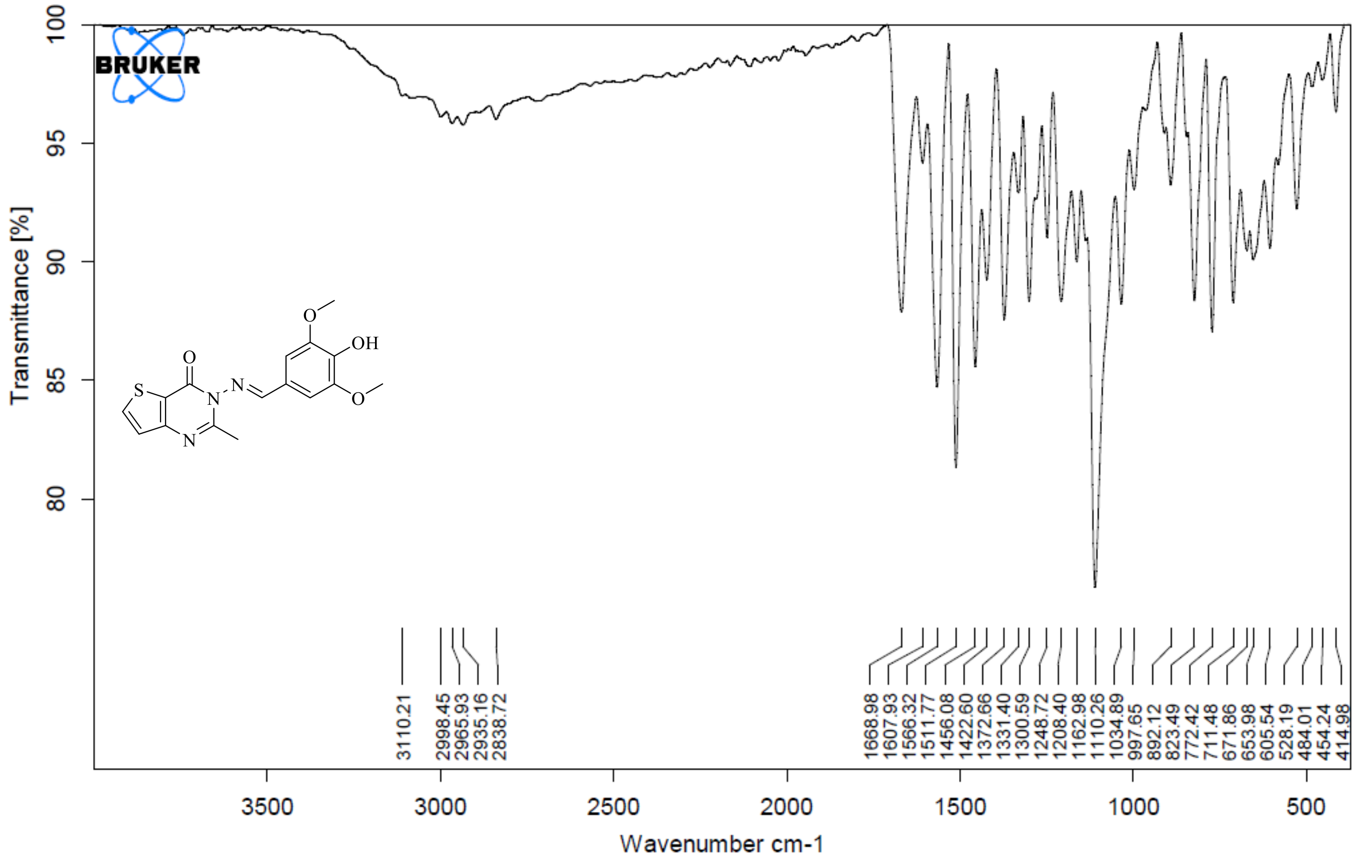

FTIR Spectrum of Compound 18

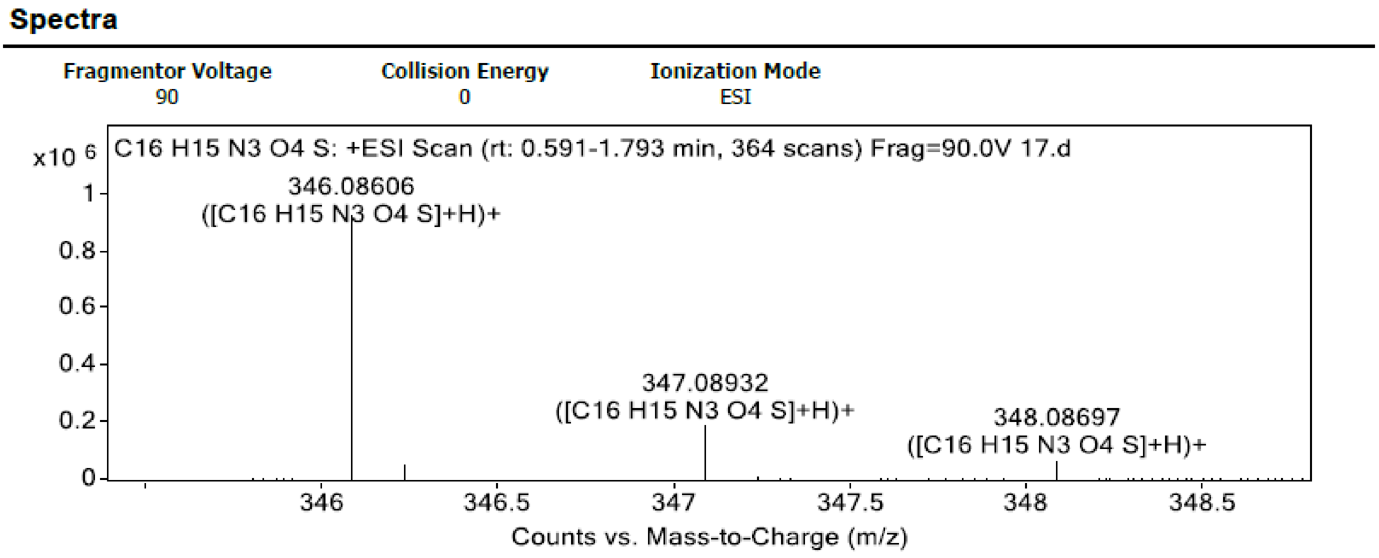

HRMS Spectrum of Compound 18

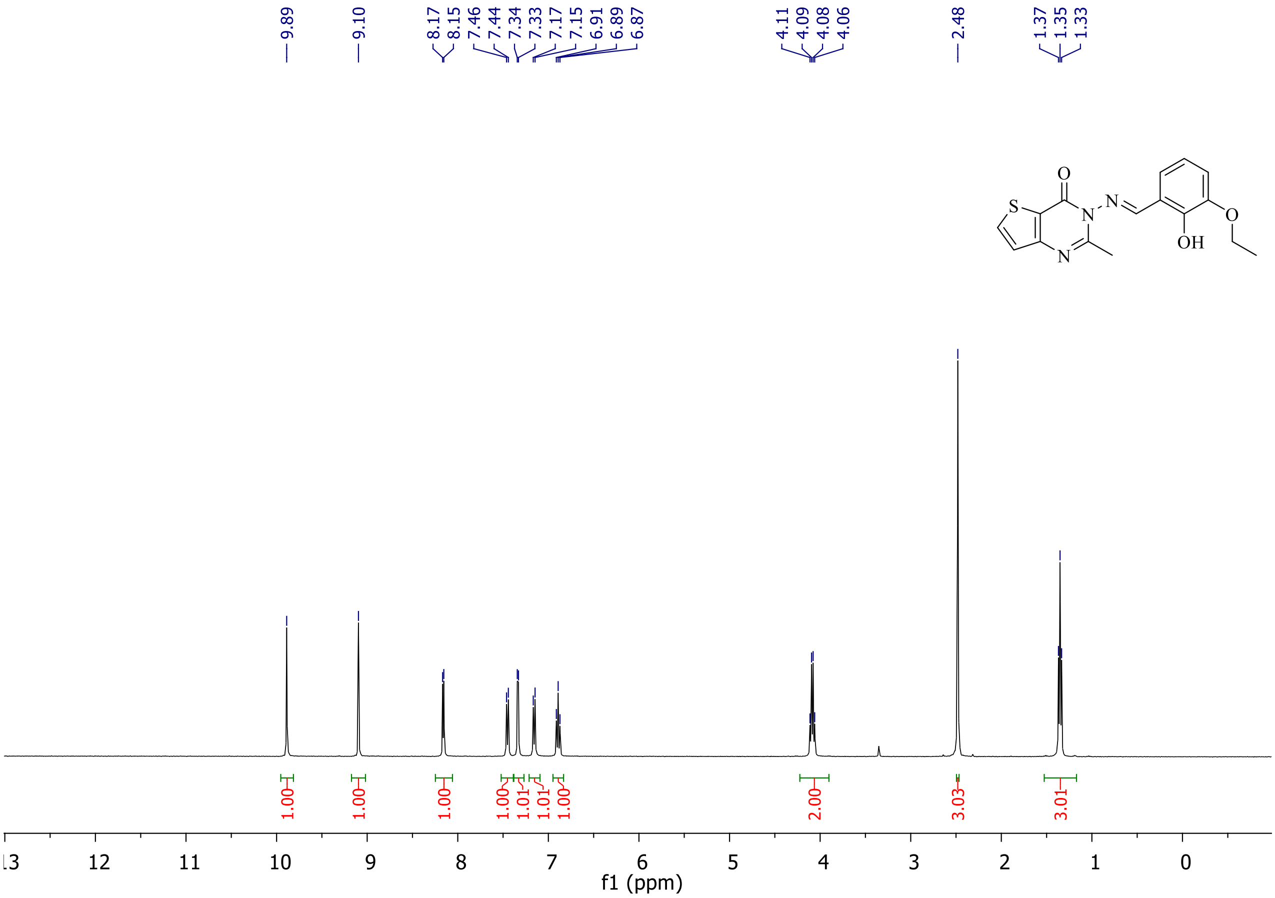

^{1}H NMR Spectrum of Compound 19

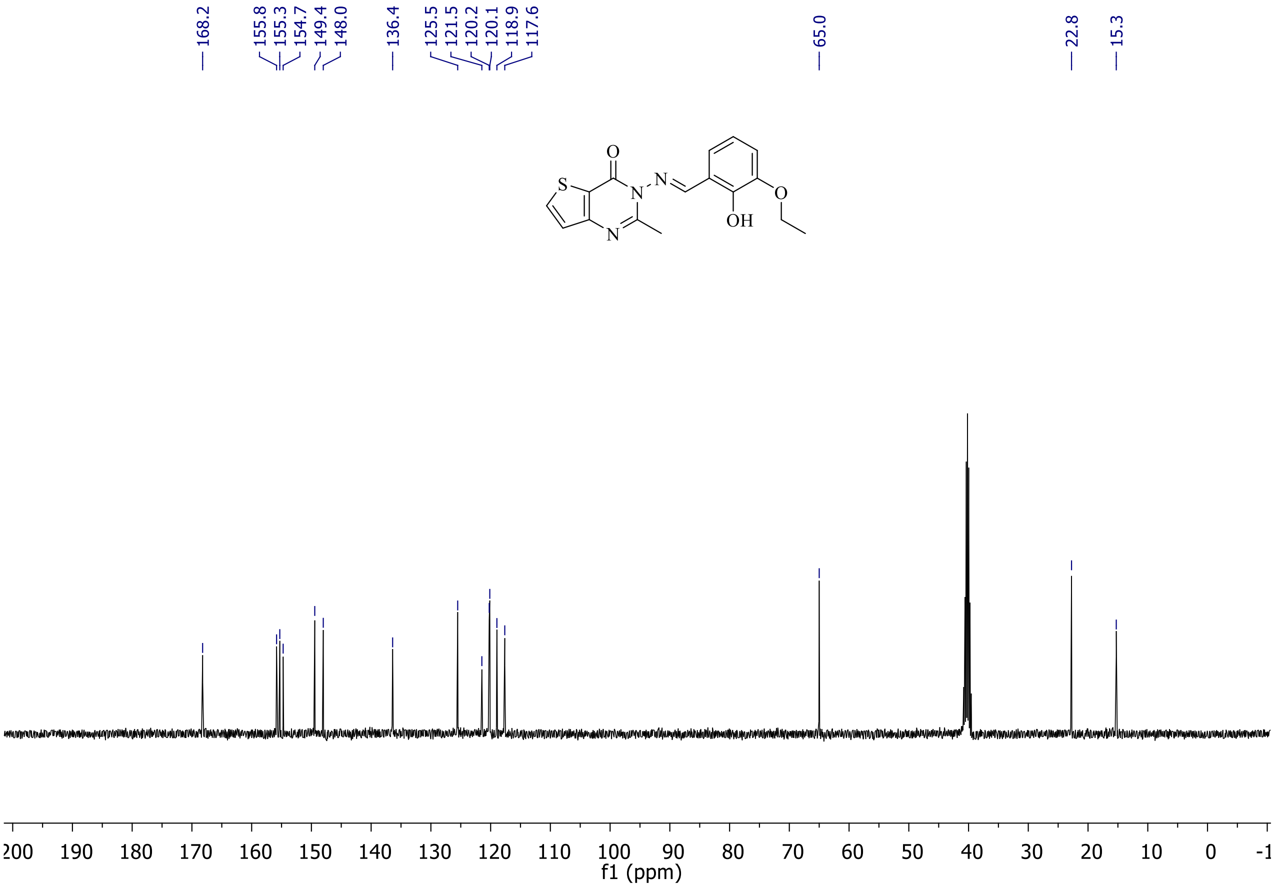

^{13}C NMR Spectrum of Compound 19

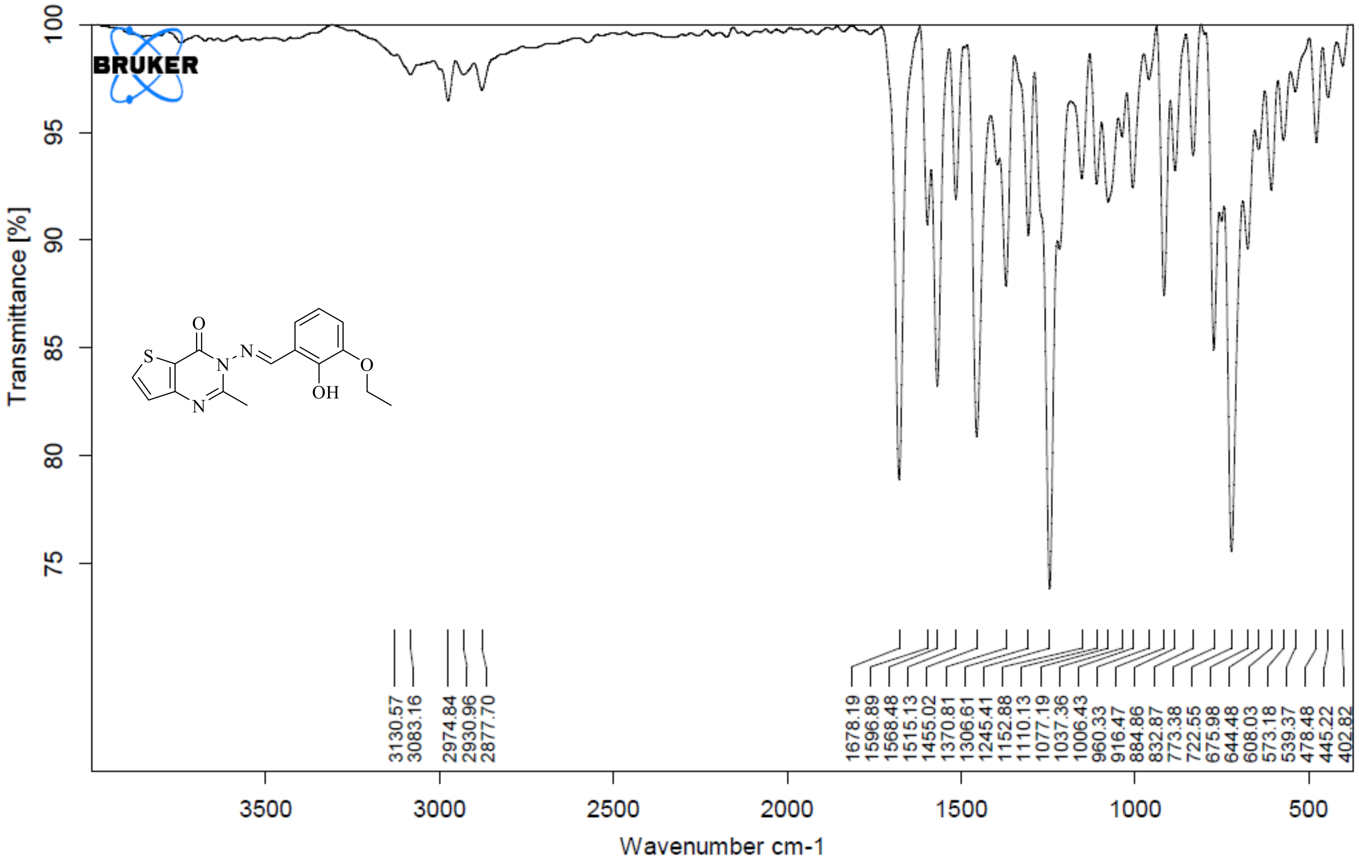

FTIR Spectrum of Compound 19

Spectra

Fragmentor Voltage	Collision Energy	Ionization Mode
90	0	ESI

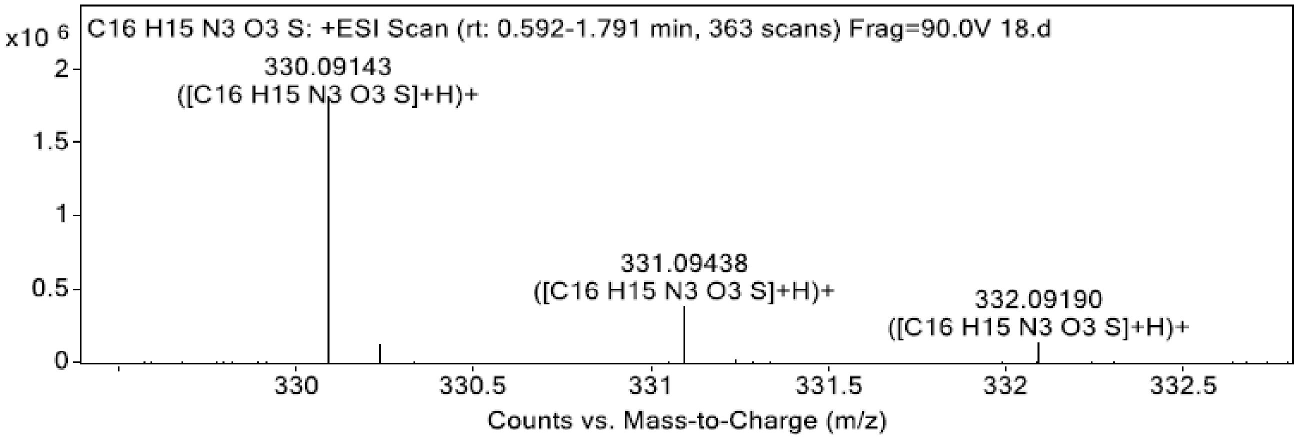

HRMS Spectrum of Compound 19

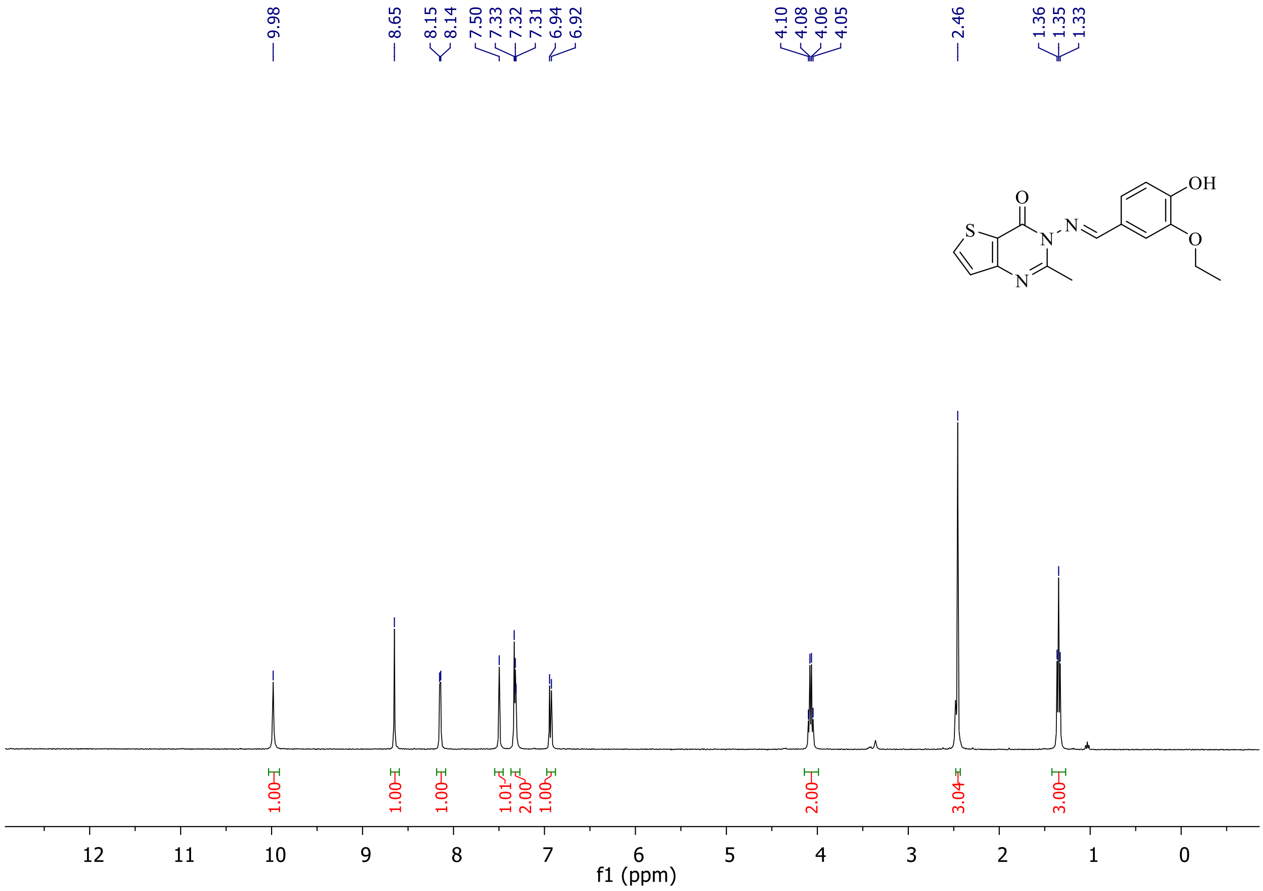

^{1}H NMR Spectrum of Compound 20

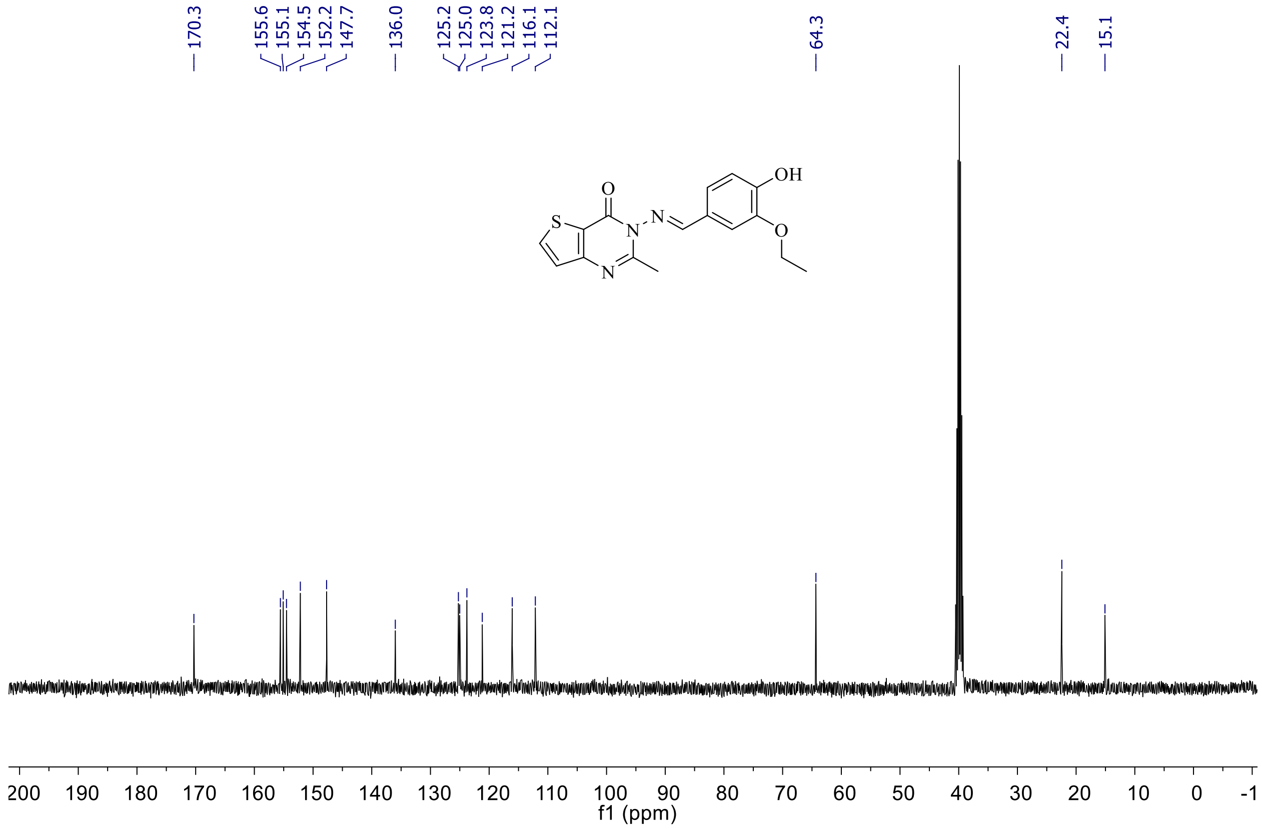

^{13}C NMR Spectrum of Compound 20

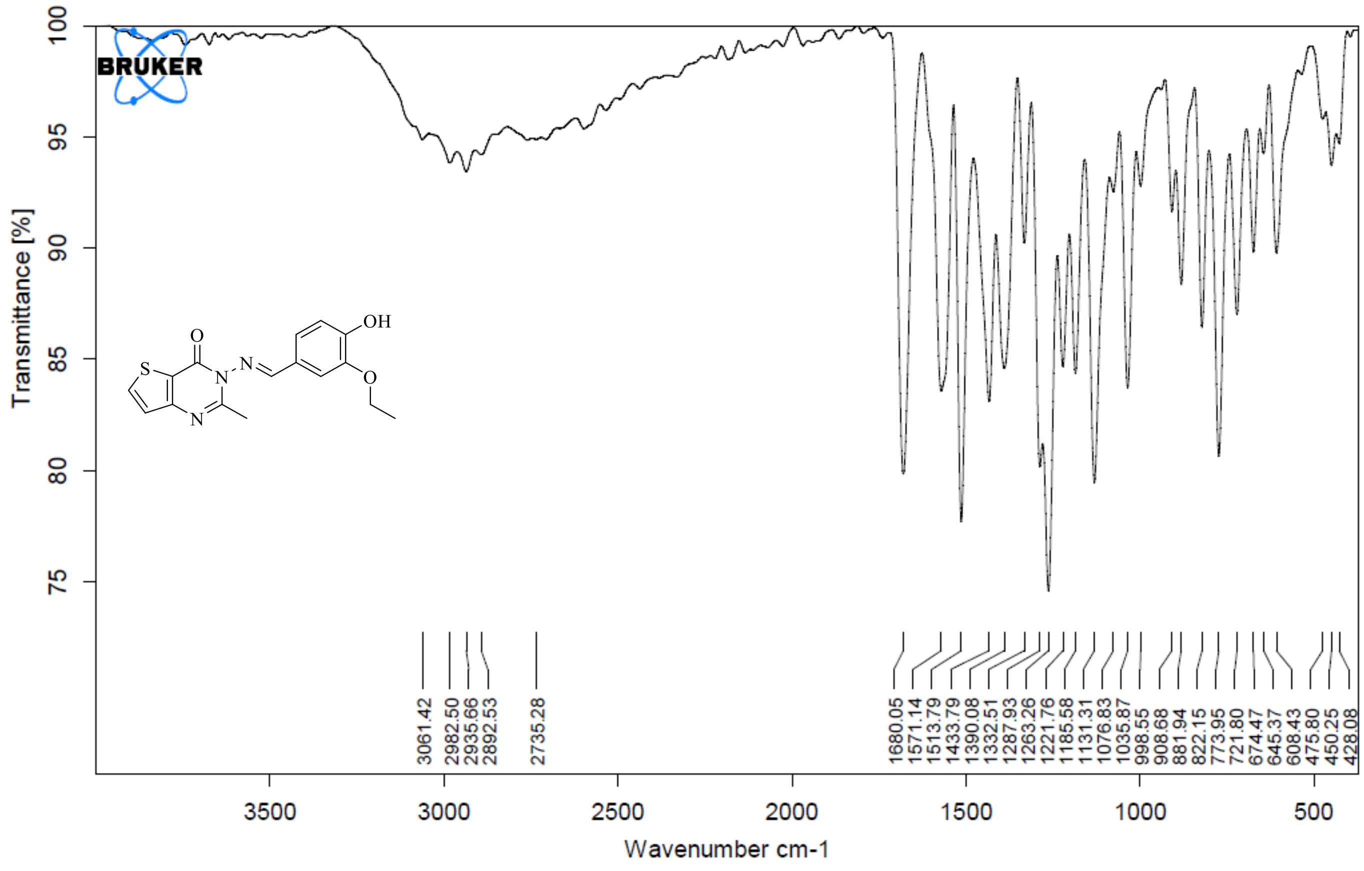

FTIR Spectrum of Compound 20

Spectra

Fragmentor Voltage	Collision Energy	Ionization Mode
90	0	ESI

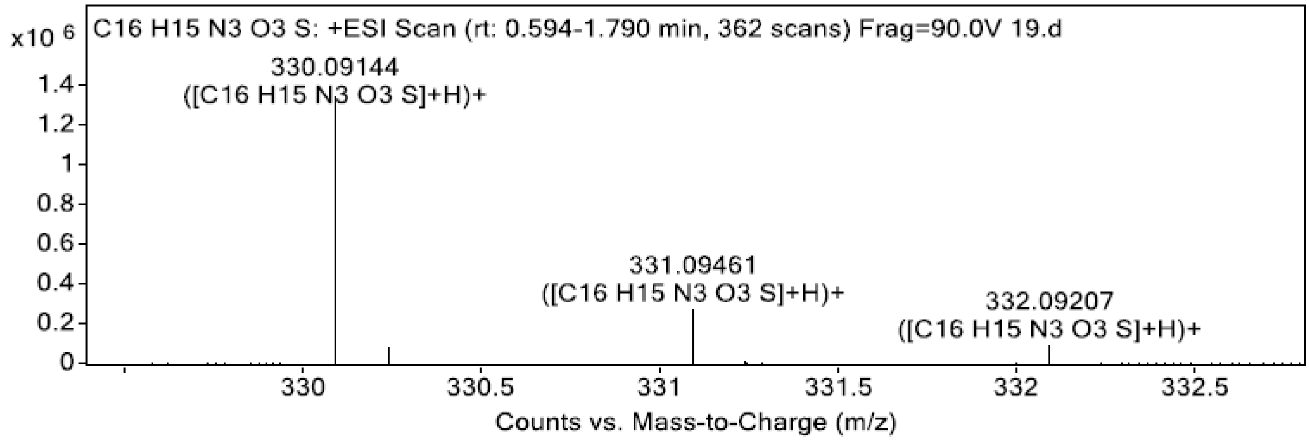

HRMS Spectrum of Compound 20

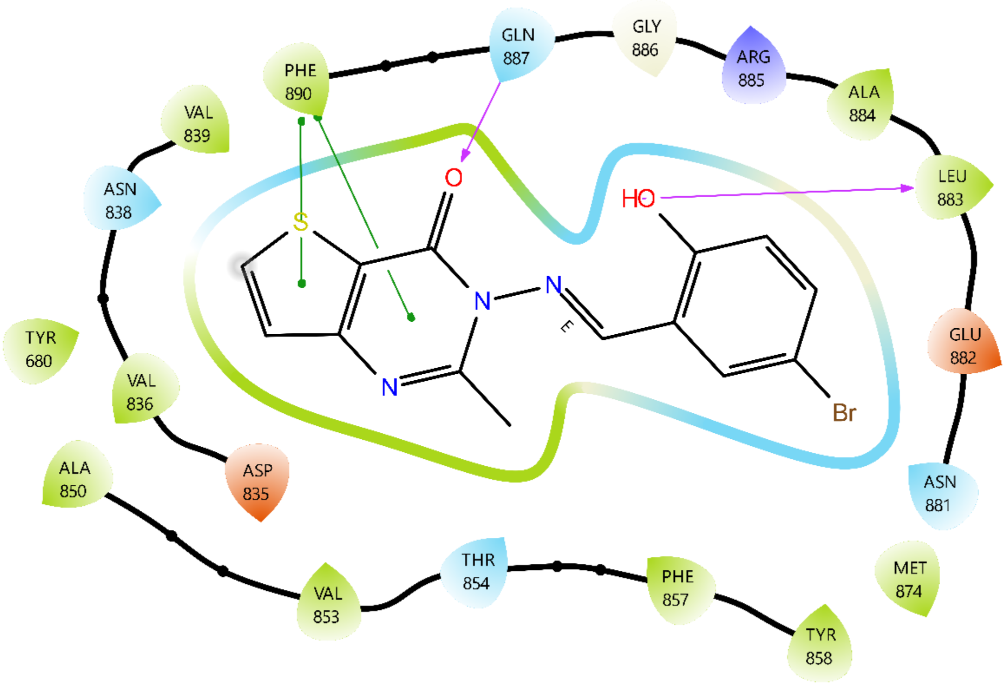

Molecular docking 2D ligand-protein interactions of **12-PDEB1** complex

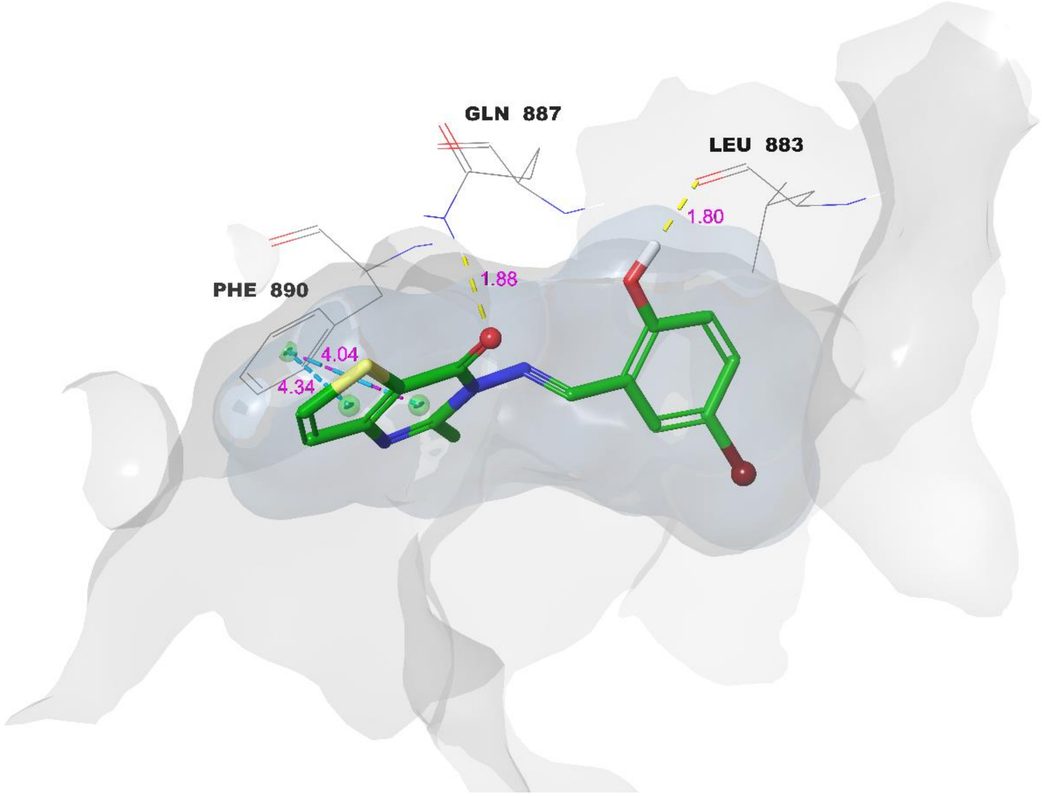

Molecular docking 3D ligand-protein interactions of **12-PDEB1** complex
